# Identification and Confirmation of Loci Associated With Canopy Wilting in Soybean Using Genome-Wide Association Mapping

**DOI:** 10.3389/fpls.2021.698116

**Published:** 2021-07-14

**Authors:** Siva K. Chamarthi, Avjinder S. Kaler, Hussein Abdel-Haleem, Felix B. Fritschi, Jason D. Gillman, Jeffery D. Ray, James R. Smith, Arun P. Dhanapal, Charles A. King, Larry C. Purcell

**Affiliations:** ^1^Department of Crop, Soil, and Environmental Sciences, University of Arkansas, Fayetteville, AR, United States; ^2^USDA-ARS, U.S. Arid Land Agricultural Research Center, Maricopa, AZ, United States; ^3^Division of Plant Sciences, University of Missouri, Columbia, MO, United States; ^4^Plant Genetic Research Unit, USDA-ARS, University of Missouri, Columbia, MO, United States; ^5^Crop Genetics Research Unit, USDA-ARS, Stoneville, MS, United States

**Keywords:** GWAS, drought, genomic selection, quantitative trait loci, soybean, canopy wilting

## Abstract

Drought causes significant soybean [*Glycine max* (L.) Merr.] yield losses each year in rain-fed production systems of many regions. Genetic improvement of soybean for drought tolerance is a cost-effective approach to stabilize yield under rain-fed management. The objectives of this study were to confirm previously reported soybean loci and to identify novel loci associated with canopy wilting (CW) using a panel of 200 diverse maturity group (MG) IV accessions. These 200 accessions along with six checks were planted at six site-years using an augmented incomplete block design with three replications under irrigated and rain-fed treatments. Association mapping, using 34,680 single nucleotide polymorphisms (SNPs), identified 188 significant SNPs associated with CW that likely tagged 152 loci. This includes 87 SNPs coincident with previous studies that likely tagged 68 loci and 101 novel SNPs that likely tagged 84 loci. We also determined the ability of genomic estimated breeding values (GEBVs) from previous research studies to predict CW in different genotypes and environments. A positive relationship (*P* ≤ 0.05;0.37 ≤ r ≤ 0.5) was found between observed CW and GEBVs. In the vicinity of 188 significant SNPs, 183 candidate genes were identified for both coincident SNPs and novel SNPs. Among these 183 candidate genes, 57 SNPs were present within genes coding for proteins with biological functions involved in plant stress responses. These genes may be directly or indirectly associated with transpiration or water conservation. The confirmed genomic regions may be an important resource for pyramiding favorable alleles and, as candidates for genomic selection, enhancing soybean drought tolerance.

## Introduction

Among the various abiotic stresses to which soybean [*Glycine max* (L.) Merr.] is exposed, drought causes the most severe yield losses and greatest year to year variation for rain-fed production systems throughout soybean-growing regions (Oya et al., 2004). Between 1986 and 2020, the soybean production area in the United States impacted by drought ranged between 3 and 59% (https://www.ncdc.noaa.gov/monitoring-content/societal-impacts/cmsi/562.tot.out), and there were 11 years in which the proportion of the soybean production area impacted by drought exceeded 20%. Total estimated economic losses due to drought during this same time period (adjusted to the consumer price index) were $217 billion in the United States (https://www.ncdc.noaa.gov/billions/events/US/1980-2020). It is likely that climate change will exacerbate the unpredictability of rainfall and will lead to an increased frequency of drought and flooding in the future (Douglas et al., 2008). Genetic improvement of soybean for drought tolerance is a cost-effective approach to stabilize yield under rain-fed production.

Past efforts to improve soybean drought tolerance through breeding have not taken full advantage of the potential genetic diversity available in germplasm collection (Frankel, 1984; Upadhyaya and Ortiz, 2001) nor have they taken direct advantage of the current understanding of physiological traits associated with drought tolerance (Sinclair et al., 2004; Sinclair and Purcell, 2005). Often, soybean breeders have focused on elite germplasm and restricted crosses to only include high-yielding elite lines, essentially “reshuffling” the same genes (Carter et al., 2004). As a result, less agronomically favorable genotypes with potential tolerance to drought have not been included, and potential progress has been inherently limited because of a lack of genetic diversity. Breeding efforts that target specific physiological traits that have agronomic advantages at the field level offer an alternative approach that draws upon previously under-utilized, diverse genetic resources (Sinclair et al., 2004; Tuberosa and Salvi, 2006).

Slow canopy wilting (CW) in soybean is a promising trait for crop improvement. Carter et al. (1999, 2006) screened exotic germplasm for drought tolerance in North Carolina and identified multiple slow-wilting genotypes, namely, PI 416937 and PI 471938. “USDA-N8002” is a soybean cultivar derived from PI 471938 (25% pedigree) and PI 416937 (12.5% pedigree), which is slow wilting and had yields averaging 7% greater than the cultivar check across 74 environments in the southern United States (Carter et al., 2016). More recently, several new genotypes that wilt more slowly than previously discovered genotypes have been identified (Kaler et al., 2017a; Steketee et al., 2020).

Slow wilting is associated with the conservation of soil moisture when soil moisture is plentiful, which can then be used when soil moisture in fast-wilting genotypes has been depleted (King et al., 2009; Ries et al., 2012). The conservation of soil water for slow wilting in several genotypes appears to be associated with decreased hydraulic conductance under high vapor pressure deficit, resulting in decreased transpiration and improved water-use efficiency relative to fast-wilting genotypes (Fletcher et al., 2007; Sinclair et al., 2008; Sadok and Sinclair, 2009; Devi and Sinclair, 2013).

Canopy wilting is a complex quantitative trait controlled by many genetic loci (Charlson et al., 2009; Du et al., 2009; Abdel Haleem et al., 2012; Hwang et al., 2015, 2016; Kaler et al., 2017a; Steketee et al., 2020). Hwang et al. (2015) used the results from five biparental mapping populations to identify clusters of eight quantitative trait loci (QTLs) for CW that were present in at least two populations, and a meta-analysis of these eight clusters identified nine meta-QTLs in eight chromosomal regions (Hwang et al., 2016). Association mapping of soybean CW identified 61 SNPs in a panel of 373 maturity group (MG) IV accessions (Kaler et al., 2017a) and 45 SNPs in a panel of 162 MG VI–VIII accessions (Steketee et al., 2020). Between the results of these two association-mapping studies for CW, similar genetic loci regions were identified on Gm01, Gm04, Gm06, Gm09, Gm12, Gm15, Gm18, Gm19, and Gm20. These two association mapping studies identified loci on Gm02 that were coincident with a meta-QTL identified previously (Hwang et al., 2016).

The objectives of this study were to confirm the slow-wilting loci identified previously by association mapping (Kaler et al., 2017a; Steketee et al., 2020) and linkage mapping (Charlson et al., 2009; Abdel Haleem et al., 2012; Hwang et al., 2016) and to identify additional novel loci associated with CW using a new panel of 200 diverse soybean accessions. We also considered the association of slow-wilting loci with loci associated with other drought-tolerant traits, such as carbon isotope (C13) ratio (as a measure of water use efficiency) (Kaler et al., 2017b; Bazzer et al., 2020a,b), oxygen isotope (O18) ratio (as a measure of transpiration) (Kaler et al., 2017b), and canopy temperature (Kaler et al., 2018; Bazzer and Purcell, 2020). An additional objective was to determine the ability of genomic estimated breeding values (GEBVs) from previous research studies to identify new slow-wilting genotypes from the United States Department of Agriculture (USDA) germplasm collection.

## Materials and Methods

### Field Experiments

In 2018, 200 MG IV accessions from the United States Department of Agriculture-Germplasm Resources Information Network (USDA-GRIN) germplasm collection (https://npgsweb.ars-grin.gov/) were selected for phenotypic evaluation of CW. Of the 200 accessions in this new panel, 100 represented the most genetically diverse genotypes (based on molecular marker data) from the original 373-accession panel used by Kaler et al. (2017a). Additionally, 100 new diverse accessions were selected from the USDA-GRIN collection based on extreme breeding values (BVs) calculated from previous association mapping results (Dhanapal et al., 2015a; Kaler et al., 2017a,b, 2018). Allelic effects from these previous studies were then used to calculate BVs for each of the MG IV accessions in the USDA germplasm collection. We selected from the germplasm collection 10 accessions with the highest and 10 accessions with the lowest BVs for CW (Kaler et al., 2017a), canopy temperature (Kaler et al., 2018), C13 ratio (Kaler et al., 2017b), and a fraction of nitrogen derived from N_2_ fixation (Dhanapal et al., 2015a). Additional 10 accessions were selected that had either high or low BVs for the combination of all four traits.

The 200 MG IV accessions along with six checks were planted in an augmented incomplete block experimental design (Federer and Crossa, 2012) with three replications under irrigated (IR) and drought (DR) treatments. Four checks, namely, PI416937 (slow wilting), PI471938 (slow wilting), A5959 (fast wilting, Monsanto Corporation, St. Louis, MO), and 08705_16 (fast wilting breeding line, Hwang et al., 2015), were planted per replication, and two checks, namely, LG11-8169-007F (MG IV elite breeding line, Gillen and Shelton, 2018) and Lee non-nod (non-nodulating check, PI 573285, Hartwig, 1994), were planted in each of 12 incomplete blocks under IR and DR treatments.

The experiment was planted at four locations in 2018 and 2019: (1) the Pine Tree Research Station (PT), AR (35.1167N, −90.9167) on a Calloway silt loam (fine-silty, mixed, active, and thermic Aquic Fraglossudalf); (2) the Rohwer Research Station (RH), AR (33.8N, −91.2833) on a Sharkey silty clay (very-fine, smectitic, and thermic Chromic Epiaquert); (3) the Bradford Research Center in Columbia (CO), MO (38.8833N, −92.2) on a Mexico silt loam (fine, smectitic, and mesic Vertic Epiaqualf); and (4) the Maricopa Agricultural Center, University of Arizona at Maricopa (MC), AZ (33.0833N, −112.0833) on a Casa Grande series (fine-loamy, mixed, and hyperthermic Typic Natrargids) soil. However, in this study, we did not include the 2019 cropping season data from RH and CO, because timely rainfall throughout the season eliminated drought.

Soil test analyses were conducted, and P and K were applied as recommended at all site-years. In the PT and RH locations, 9 row plots were sown with a drill having 19 cm between rows and with a plot length of 4.57 m. The plots consisted of four rows at CO with rows 3.96 m in length and with 0.15 m between rows. At MC, there were three-row plots with rows 4.87 m in length and with 0.19 m row spacing. Herbicides and insecticides were applied as recommended to control weeds and insects at all site-years. For the IR treatment, drip irrigation was used at CO, flood irrigation was used at PT, and furrow irrigation was used at RH and MC. At PT and RH, irrigation was applied to IR and DR treatments before the V6 stage when the estimated soil moisture deficit exceeded 50 mm (Purcell et al., 2007). After V6, no further irrigation was applied to the DR treatment.

### Phenotypic Evaluations and Statistical Analysis

Canopy wilting was rated based on a visual scoring scale where 0 represented no wilting, 20 represented slight wilting and leaf rolling at the top of the canopy, 40 represented severe leaf rolling at the top of the canopy and moderate leaf wilting throughout the canopy and loss of petiole turgidity, 60 represented severe wilting throughout the canopy and loss of petiole turgidity, 80 represented severe petiole wilting and dead leaves scattered throughout the canopy, and 100 represented plant death (King et al., 2009; Kaler et al., 2017a). After removing the RH19 and CO19 data, there were six site-years and two treatments. We subsequently refer to the combination of site-year and treatment as an environment. These 12 environments were designated for 2018 as follows: IR (PT18IR) and DR (PT18DR) at Pine Tree, AR; IR (RH18IR) and DR (RH18DR) at Rohwer, AR; IR (CO18IR) and DR (CO18DR) at Columbia, MO; and IR (MC18IR) and DR (MC18DR) at Maricopa, AZ. For 2019, there was a similar naming convention for IR (PT19IR) and DR (PT19DR) at Pine Tree, AR; and IR (MC19IR) and DR (MC19DR) at Maricopa, AZ.

Canopy wilting was rated four times at CO18 and MC19, two times at MC18, and one time at RH18, PT18, and PT19. For all the environments, measurements were performed within 2 h of solar noon under a clear sky. For all rating dates, plant development ranged from late vegetative stages to R4 (Fehr and Caviness, 1977). Between emergence and the last rating date for the DR treatment, irrigation was applied three times at RH18, one time at PT18, zero time at PT19, four times at CO18, and three times at MC18 and MC19 before rating wilting. At RH18, PT18, PT19, and CO18, there was minimal stress on rating dates. The soil had been replenished with rainfall 3 days prior to rating wilting at RH18, 6 days at PT18, 6 days at PT19, and from 2 to 7 days for the four CO18 wilting ratings. At MC18, there was no rainfall during the measurement period, and CW scores were recorded 1 day after the irrigation in the IR treatment. After the initial rating at MC18, the DR treatment was irrigated, and CW scores were recorded after 14 and 21 days. At MC19, the DR treatment was irrigated and wilting was rated after 18, 21, 27, and 31 days, while the IR treatment was rated 1 day after irrigation (Supplementary Table 1). Cumulative potential evapotranspiration rate was calculated to quantify soil moisture deficit for the DR treatment for each site-year (Purcell et al., 2007). At the time of rating for all the environments, there was visual evidence of wilting among some genotypes in the IR treatment, and therefore, both the IR and DR treatments were scored.

Canopy wilting was measured multiple times at CO18, MC18, and MC19 (Supplementary Table 1), and the average values from individual rating dates were used for genome-wide association (GWAS) analysis. With the exception of MC19IR, individual ratings within a site-year and treatment were significantly correlated (*P* ≤ 0.001) with correlation coefficients ranging between 0.43 and 0.77 (data not shown). Individual rating values also agreed closely (0.72 ≤ r ≤ 0.91) with the average rating for a given site-year-treatment combination. Previous reports of CW have also found similar ranking among genotypes and high correlations between individual rating dates (King et al., 2009; Steketee et al., 2020), and previous mapping studies of CW have used both individual rating dates or average values from multiple rating dates (Charlson et al., 2009; Abdel Haleem et al., 2012; Hwang et al., 2015; Kaler et al., 2017a). Descriptive statistics and Pearson's correlation coefficients were computed using the PROC UNIVARIATE and PROC CORR procedures (α = 0.05) of SAS version 9.4 (SAS, Institute 2013), respectively.

For ANOVA, the PROC MIXED procedure (α = 0.05) of SAS 9.4 was used with a model: Y_ijklm_ = μ + G_i_ + S_j_ + T_k_ + GS_ij_ + GT_ik_ + ST_jk_ + GST_ijk_ + R_l(j)_ + B_m(l)_ + GR_il(j)_ + GB_im(l)_ + TR_kl(j)_ + TB_km(l)_ + GTR_ikl(j)_ + (residual error_ε*ijklm*_]. In this model, fixed effects were G_i_ = effect of the ith genotype, S_j_ = effect of the jth site year, and T_k_ = effect of the kth treatment, plus all of the fixed effect two-way interactions (GS_ij_, GT_ik_, and ST_jk_), and the three-way interaction GST_ijk_. The random effects included the following: R_l(j)_ = effect of the lth replicate nested in site-year j, B_m(l)_ = effect of the mth incomplete block nested in rep l, GR_il(j)_ = effect of the interaction of the ith genotype with the lth replicate, GB_im(l)_ = effect of the interaction of the ith genotype with the mth incomplete block, TR_kl(j)_ = effect of the interaction of the kth treatment with the lth replicate, TB_km(l)_ = effect of the interaction of the kth treatment with the mth incomplete block, GTR_ikl(j)_ = effect of the interaction of the ith genotype, kth treatment, and lth replicate, and the residual error consists of the interaction of the ith genotype, the kth treatment, and the mth incomplete block.

PROC VARCOMP of SAS 9.4 with the restricted maximum likelihood (REML) method was used to estimate the variance components for the calculation of broad-sense heritability on an entry-mean basis. The best linear unbiased prediction (BLUP) values for each independent environment, as well as across environments, were estimated using META-R, and these values were then used for association analysis. Association analysis was conducted in three ways: (1) for each of the 12 environments, (2) averaged over site-years for IR (Ave_IR) and DR treatments (Ave_DR), and (3) averaged across all environments (AAE). CW BLUP values for the 12 environments, Ave_IR, Ave_DR, and AAE are provided in Supplementary Table 2.

### Genotyping and Linkage Disequilibrium

Marker data, consisting of 42,450 SNPs for all 200 accessions, were obtained from Soybase (Glyma.w82.a1,www.soybase.org) (Song et al., 2013, 2015). The Glyma.w82a1genome assembly was used, because these same markers were used in the previous association mapping of CW (Kaler et al., 2017a), and one objective was to confirm the previously identified markers. Marker data of 34,680 SNPs were filtered for quality control, which included removing monomorphic markers, heterozygous markers, markers with minor allele frequency (MAF) ≤ 5%, and markers with a missing rate higher than 10%. The remaining missing markers (those with ≤ 10%) were imputed using an LD-kNNi method, which is based on a k-nearest neighbor genotype (Money et al., 2015). These markers were used to measure pairwise linkage disequilibrium (LD) separately for euchromatic and heterochromatic regions based on squared correlation coefficients (*r*^2^) of alleles in the TASSEL 5.0 software (Hill and Weir, 1988; Bradbury et al., 2007). The results indicated that at *r*^2^ = 0.25, an average LD across all chromosomes decayed at an average of 175 kb in the euchromatic region and at an average of 5,100 kb in the heterochromatic region. These results on LD in soybean are consistent with previous studies (Schmutz et al., 2010; Hwang et al., 2014; Dhanapal et al., 2015b; Kaler et al., 2017a).

### Genome-Wide Association Analysis

The FarmCPU model was chosen as the most appropriate model to control false positives and false negatives (Liu et al., 2016; Kaler et al., 2017a, 2020b). A significant threshold value (–Log10 *P* ≥ 3.5), which is equivalent to *P* ≤ 0.0003, was used to identify SNPs. This threshold *P-value* was chosen based on a formula that uses marker-based heritability (Kaler and Purcell, 2019) and is similar to threshold values used previously (Kaler et al., 2017a, 2020a,b; Steketee et al., 2020; Kaler and Purcell, 2021). To identify the common significant SNP present in more than one environment, a threshold value of *P* ≤ 0.05 was used but only if the representative SNP had an association of *P* ≤ 0.0003 in a second environment (Kaler et al., 2017a,b, 2018, 2020a). The allelic effect of a significant SNP was calculated by taking the difference in mean CW between genotypes with the major allele and those with minor allele. Alleles from either the major or minor class were considered as favorable if they were associated with reduced CW. A negative sign in the allelic effect indicated that the major allele was favorable for CW, and a positive sign in the allelic effect indicated that the minor allele was favorable for CW.

To find coincident SNPs or overlap of loci identified in this research study with those loci reported in previous studies (Charlson et al., 2009; Abdel Haleem et al., 2012; Hwang et al., 2016; Kaler et al., 2017a,b, 2018; Bazzer and Purcell, 2020; Bazzer et al., 2020a,b; Steketee et al., 2020), we used Bedtools Intersect Intervals Tool (Quinlan and Hall, 2010) in Galaxy with an overlapping window of ± 175 kb. This window size was chosen because the average LD across all chromosomes decayed at an average of 175 kb in the euchromatic region. SNPs that were not coincident with previous studies were considered as novel loci.

### Genomic Estimated Breeding Values (GEBVs) and Prediction Accuracy

We evaluated the accuracy of predicting CW by correlating (PROC CORR, SAS v. 9.4, SAS Institute, 2013) observed wilting scores using three different datasets with GEBVs (Meuwissen et al., 2001) from the BayesB genomic prediction model (Pérez et al., 2010). In the first scenario, the averaged CW scores of 373 (Kaler et al., 2017a) and 153 (Steketee et al., 2020) accessions were used as the training set for the genomic prediction of the 100 new accessions used in this study. In the second scenario, the averaged CW scores of the 100 new accessions used in this study and the 153 accessions reported by Steketee et al. (2020) were used as the training set for the genomic prediction of the 373 accessions reported by Kaler et al. (2017a). In the third scenario, the averaged CW scores of the 100 new accessions used in this study plus the 373 accessions reported by Kaler et al. (2017a) were used as the training set for the genomic prediction of the 153 accessions reported by Steketee et al. (2020).

Marker data consisted of 34,652 filtered SNPs for all accessions that were obtained from Soybase (Glyma.w82.a1,www.soybase.org) (Song et al., 2013, 2015). For the 162 accessions reported by Steketee et al. (2020), genotyping data from Soybase were available for only 153 accessions. Imputation and filtration were accomplished using TASSEL as described in the genotyping and LD sections.

### Predicting Canopy Wilting for Soybean Germplasm Using GEBVs

The 19,648 accessions in the USDA soybean collection (https://www.ars-grin.gov/), consisting of MGs from MG000 to MGX, were used as a testing population. The 373 accessions from Kaler et al. (2017a), 153 accessions from Steketee et al. (2020), and 100 new lines from this study were used as a training population to predict the CW from the soybean germplasm using the BayesB genomic prediction model (Pérez et al., 2010). The 10 slowest and 10 fastest wilting genotypes from each MG were identified based on GEBVs from the testing population. Genotyping data for all germplasm accessions were obtained from Soybase (Glyma.w82.a1, www.soybase.org) (Song et al., 2013, 2015).

### Candidate Gene Identification

Significant SNPs were used to identify candidate genes for CW using the *G. max* genome assembly version Glyma.Wm82.a1.v1.1 (www.soybase.org) (Schmutz et al., 2010). Genes located near SNPs associated with CW were considered as potential candidates if they were within ± 10 kb or ± 100 kb of a significant SNP in euchromatic and in heterochromatic regions, respectively. These distances were chosen to reflect the average distance between SNPs in these regions. Candidate genes were grouped into three gene ontology (GO) categories, namely, biological process, cellular component, and molecular function. Further, based on biological functions, genes were identified and categorized if they had any association with drought tolerance-related responses, such as abscisic acid, water transport, root development, leaf senescence, jasmonic acid, heat acclimation, stomata, and salicylic acid (Schulze, 1986; Jackson et al., 2000; Schmutz et al., 2010; Jarzyniak and Jasinski, 2014; Khan et al., 2015; Sah et al., 2016).

## Results

### Phenotype Descriptions

There were large differences among site-years between emergence and the last rating date in average maximum and minimum temperatures and total precipitation (Supplementary Table 1). Average maximum temperature was highest for MC18 (40°C) and lowest for CO18 (30°C), whereas average minimum temperature was highest for RH18 (23°C) and lowest for CO18 (18°C). Total precipitation between emergence and the last rating date was highest for PT19 (376 mm) and lowest for MC18 (5 mm). To quantify drought for each environment, we estimated the cumulative potential evapotranspiration rate (Purcell et al., 2007), which was highest in MC18 (318 mm) and MC19 (385 mm) (Supplementary Table 1).

There was a broad range of CW observed within a single environment, when averaged across IR or DR treatments, and when averaged across all the 12 environments (Table 1). Within the IR treatment, CW scores had ranges of 35 (CO18IR), 17.5 (MC18IR), 3.3 (MC19IR), 40 (PT18IR), 35 (PT19IR), 30 (RH18IR), and 40 (Ave_IR) (Table 1). Averaged over DR treatments, the range of wilting values was greater for the IR treatments by 22.5. For PT18, the average wilting score for the IR treatment was numerically greater (20.4) than that of the DR treatment (18.8), but the median values were the same (20), indicating that wilting scores between treatments were essentially the same. Although soil moisture was plentiful at RH, PT, and CO for both IR and DR treatments on measurement dates in 2018 and 2019, the IR treatment received irrigation earlier in the season but the DR treatment did not, which may have resulted in differential responses.

**Table 1:** Descriptive statistics and broad-sense heritability (*H*^2^) of canopy wilting (CW) scores for 12 environments, Columbia (CO18), Maricopa (MC18 and 19), Pine Tree (PT18 and 19), Rohwer (RH18) under irrigated (IR) and drought (DR) treatments; averaged across irrigated (Ave_IR) and drought (Ave_DR) treatments; and averaged across all environments (AAE).

	**Mean**	**Median**	**Standard Deviation**	**Range**	**Minimum**	**Maximum**	***H^**2**^(%)***
CO18IR	9.1	7.5	5.9	35.0	5.0	40.0	76
MC18IR	21.9	20.0	3.3	17.5	17.5	35.0	39
MC19IR	20.1	20.0	0.4	3.3	20.0	23.3	0
PT18IR	20.4	20.0	7.6	40.0	5.0	45.0	67
PT19IR	17.7	17.0	6.1	35.0	5.0	40.0	72
RH18IR	14.4	15.0	4.7	30.0	5.0	35.0	73
CO18DR	16.1	15.0	9.8	62.5	5.0	67.5	78
MC18DR	28.8	27.5	6.9	32.5	20.0	52.5	75
MC19DR	27.6	26.3	6.8	28.8	20.0	48.8	84
PT18DR	18.8	20.0	7.7	40.0	5.0	45.0	62
PT19DR	23.0	20.0	8.1	50.0	5.0	55.0	74
RH18DR	20.6	20.0	6.6	35.0	5.0	40.0	81
Ave_IR	17.2	20.0	6.8	40.0	5.0	45.0	71
Ave_DR	22.5	21.3	8.9	62.5	5.0	67.5	79
AAE	19.9	20.0	8.4	62.5	5.0	67.5	86

**Figure 1:**
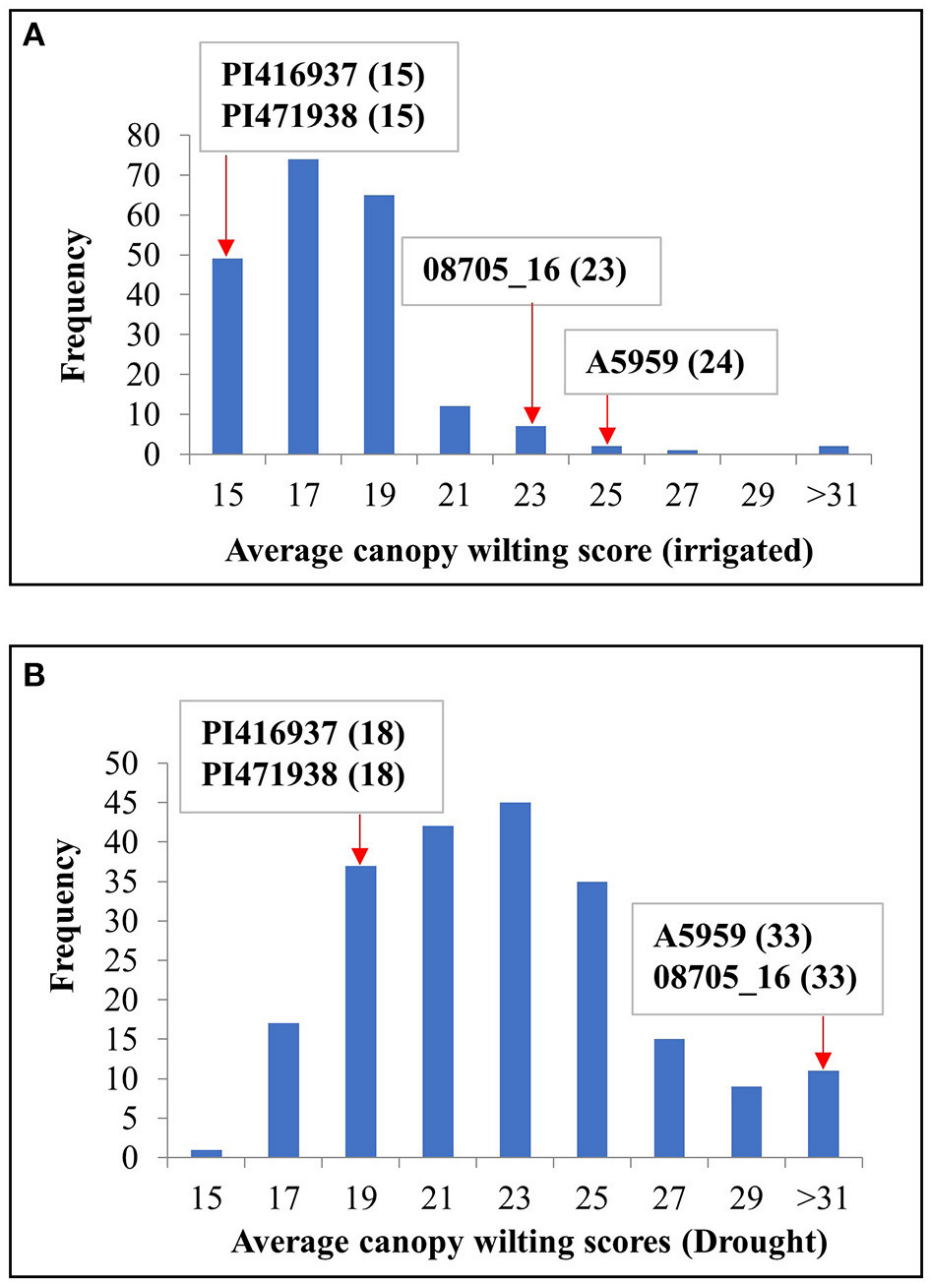
Frequency distribution of maturity group (MG) IV genotypes for canopy wilting (CW) scores averaged across all environments for both **(A)** irrigated (IR) and **(B)** drought (DR) treatments. PI 416937 and PI 471938 were slow-wilting controls, whereas genotypes A5959 and 08705_16 were fast-wilting controls. Numbers in parentheses indicate the wilting scores for the checks.

Figure 1 shows the frequency distribution of the average genotypic means of CW for the IR and DR treatments, indicating that the DR treatment had a wider range of CW compared with the IR treatment. On one extreme, there were 11 genotypes for the average IR treatments and 18 genotypes for the average DR treatments that had wilting scores significantly (*P* ≤ 0.05) lower than those of the two slow-wilting checks, namely, PI416937 and PI471938. At the other extreme, there were three genotypes for the IR treatment and four genotypes for the DR treatment with wilting scores significantly (*P* ≤ 0.05) higher than those of the two fast-wilting checks, namely, A5959 and 08705_16 (Supplementary Table 3).

ANOVA of CW data indicated that there were significant effects for the fixed effect terms of genotype, site-year, treatment (IR and DR), and all two-way and three-way interactions (*P* < 0.0001, Table 2). Generally, there were significant positive correlations for CW scores under both the IR and DR treatments for all the environments, except MC19IR (Supplementary Table 4). For MC19IR, extreme heat and high evaporative demand resulted in CW scores of ~20 that were similar among genotypes with a range of only 3.3 (Table 1). The ANOVA by environment indicated that MC19IR was the only environment in which genotype was not significant (data not shown). The correlation averaged over all IR treatments with the average of all DR treatments was 0.8 (Supplementary Table 4). Excluding MC19IR, broad-sense heritability (*H*^2^) ranged from 39 to 76% for the IR treatment and from 62 to 84% for the DR treatment. Averaged across all the site-years, *H*^2^ was 71% for the IR treatment, 79% for the DR treatment, and 86% when averaged over all the environments (Table 1).

**Table 2:** Analysis of variance (ANOVA) for CW.

**Effect**	**Degree of Freedom**	**F-statistic**	***P*-value**
Genotype (G)	205	17.0	<0.0001
Site-year (S)	5	27.1	<0.0001
Treatment (T)	1	403.6	<0.0001
G × S	1,025	3.1	<0.0001
G × T	205	3.6	<0.0001
S × T	5	28.7	<0.0001
G × S × T	1,010	2.1	<0.0001

*Only fixed effects are shown*.

### Genome-Wide Association Analysis

**Figure 2:**
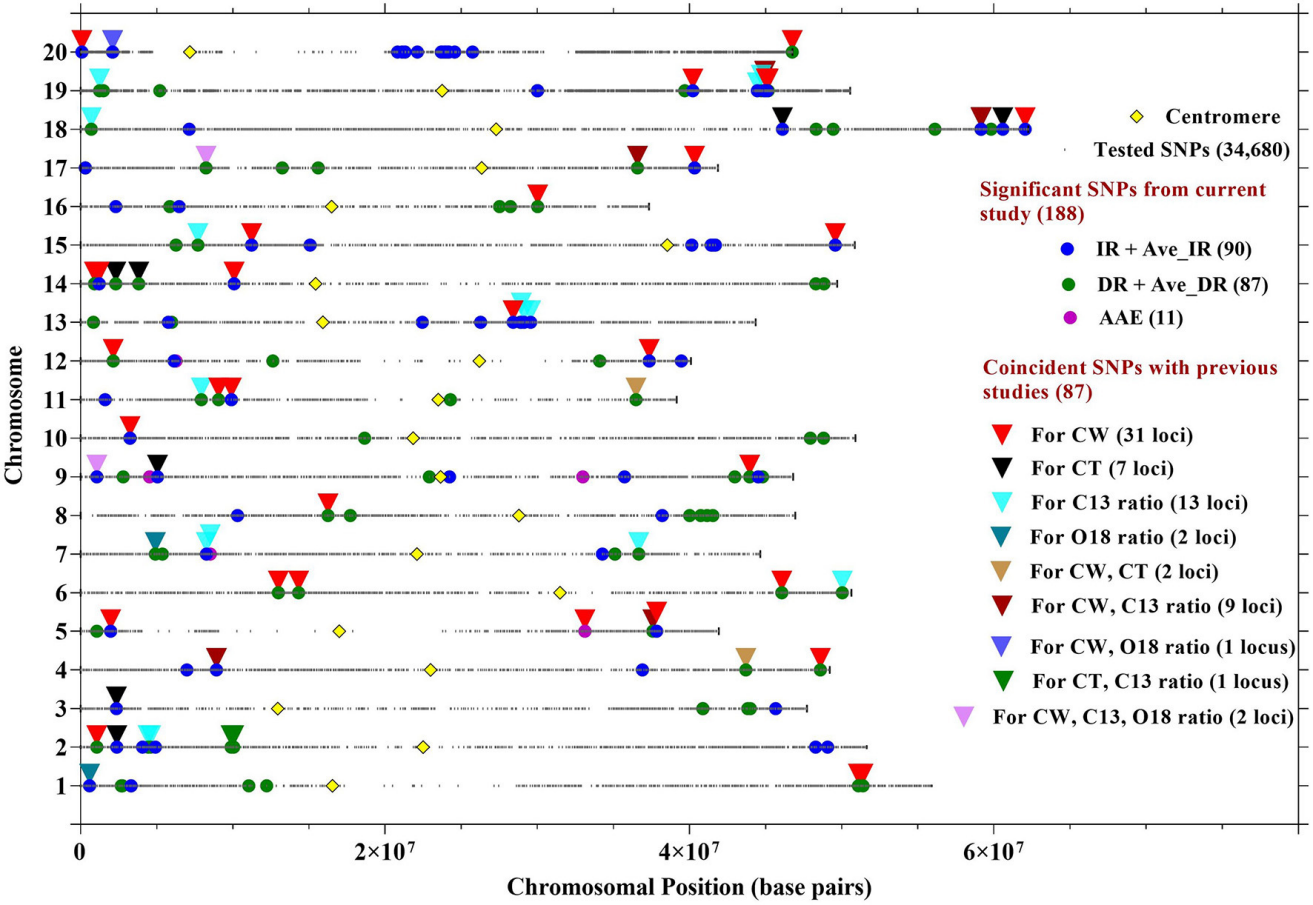
Location of SNPs significantly associated with CW in 12 environments, averaged over site-years for IR treatment (Ave_IR), averaged over site-years for drought treatment (Ave_DR), and averaged across all environments (AAE). Locations of SNPs associated with CW from the current research study were compared with SNPs previously identified with CW, canopy temperature (CT), C13, and O18 ratios. Details about the coincident SNPs are described in Table 3.

The aim of this study was to confirm the canopy-wilting loci identified previously by association mapping (Kaler et al., 2017a; Steketee et al., 2020) and to identify additional novel loci associated with CW. GWAS identified a total of 188 significant SNPs associated with CW that likely tagged 152 loci. This includes 87 significant SNPs identified as coincident SNPs for CW (Charlson et al., 2009; Abdel Haleem et al., 2012; Hwang et al., 2016; Kaler et al., 2017a; Steketee et al., 2020), canopy temperature (Kaler et al., 2018; Bazzer and Purcell, 2020), C13 ratio (Kaler et al., 2017b; Bazzer et al., 2020a,b), or O18 ratio (Kaler et al., 2017b) from previous studies that likely tagged 68 loci (Table 3), and 101 significant SNPs identified as novel SNPs that likely tagged 84 loci (Table 4). These 152 loci (68 + 84) were identified from the sum of significant loci [–Log10 (*P*) ≥ 3.5; *P* ≤ 0.0003] for individual environments, plus loci averaged over site-years by the IR and DR treatments, and plus loci averaged over all the environments (Figure 2 and Supplementary Figures 1–3).

**Table 3:** Significant coincident single-nucleotide polymorphisms (SNPs) with previous studies associated with CW with the 12 environments, Columbia (CO18), Maricopa (MC18 and 19), Pine Tree (PT18 and 19), and Rohwer (RH18) under IR and DR treatments, and for averaged across site-years for irrigated (Ave_IR) and drought (Ave_DR) treatments, and averaged across all environments (AAE) at the significance threshold of –Log10 (*P*) ≥ 3.5; *P* ≤ 0.0003.

**Locus**	**SNP_ID**	**CHR**	**Position**	**-Log10 (*P*)**	**Allele^a^**	**Allelic effect^b^**	**ENV**	**Common ENV^c^**	**Coincident genomic regions with previous studies^d^**
**Irrigated (IR)**									
1	ss715580748	1	595259	3.6	T/C	−2.7	PT18IR		GWAS-δ18O QTL^**2**^
2	ss715581576	2	2391001	5.0	T/C	2.3	PT19IR	PT18IR	CT-QTL^**7**^
3	ss715584835	3	2359805	3.6	G/A	0.9	RH18IR	MC19DR	GWAS-CT QTL^**3**^
4	ss715589557	4	8924261	3.9	T/C	−0.8	MC18IR		GWAS-δ13C QTL^**2**^, Canopy_wilt_2_2^**9**^
5	ss715590117	5	1983770	3.6	T/C	−1.3	CO18IR		mqCanopy_wilt_007 and mqCanopy_wilt_019^**8**^, Canopy_wilt_2_3^**9**^
6	ss715591559	5	37839978	4.8	C/T	1.3	PT19IR	AAE/MC19IR	GWAS-CW QTL^**1**^
7	ss715598790	7	8267191	4.4	A/C	1.4	CO18IR		δ13C-QTL^**5**^
8	ss715602950	9	1066026	6.6	T/C	−1.8	CO18IR	CO18DR	GWAS-CW QTL^**1**^, GWAS-δ13C and δ18O QTL^**2**^
9	ss715605115	9	5057308	4.0	T/C	−2.7	CO18IR		GWAS-CT QTL^**3**^
10	ss715606242	10	3253404	4.3	A/C	−0.1	RH18IR	RH18DR/PT18DR/MC19IR	GWAS-CW QTL^**4**^
11	ss715611329	11	9912187	4.2	G/A	−3.8	PT18IR	AAE/CO18DR	mqCanopy_wilt_020^**8**^
12	ss715612746	12	37356120	8.1	C/T	−2.7	RH18IR	RH18DR/PT18DR/PT18IR/MC19IR	GWAS-CW QTL^**1**^
13	ss715614917	13	28949836	5.5	A/G	1.3	CO18IR	Ave_IR/PT18IR	GWAS-δ13C QTL^**2**^
14	ss715617331	14	10092238	3.6	G/A	0.7	PT19IR	PT18IR	GWAS-CW QTL^**4**^
15	ss715620301	15	11228851	7.4	A/G	−3.2	PT19IR	PT19DR/PT18IR	GWAS-CW QTL^**1**^
16	ss715622635	15	49582581	5.8	A/G	1.2	RH18IR	PT18IR/Ave_IR/PT19IR	GWAS-CW QTL^**4**^
17	ss715627700	17	40335241	4.3	G/A	−1.1	RH18IR	PT19DR/CO18IR	mqCanopy_wilt_015^**8**^
18	ss715630654	18	46111574	6.2	A/C	−3.3	RH18IR	AAE/PT19DR/MC18DR/PT18IR	GWAS-CT QTL^**3**^
19	ss715632103	18	59162269	4.1	C/T	−5.1	PT18IR	Ave_IR/PT18DR/Ave_DR/AAE/PT19IR/MC18IR/CO18DR/PT19DR	GWAS-CW QTL^**1**^, δ13C-QTL^**6**^
20	ss715632312	18	60577930	3.8	C/T	−0.7	CO18IR		GWAS-CT QTL^**3**^, CT-QTL^**7**^
21	ss715632502	18	62056969	3.6	T/C	−0.5	CO18IR	RH18IR/Ave_IR	GWAS-CW QTL^**1**^
22	ss715634991	19	40220231	3.8	G/A	1.0	CO18IR		GWAS-CW QTL^**1**^
23	ss715635419	19	44955912	4.0	G/T	−0.5	MC18IR	PT19DR/MC18DR/AAE/PT18IR/MC19IR	GWAS-CW QTL^**4**^, δ13C-QTL^**6**^
	ss715635420	19	44964042	4.0	T/C	−0.5	MC18IR	PT19DR/MC18DR/AAE/PT18IR	GWAS-CW QTL^**4**^, δ13C-QTL^**6**^
	ss715635421	19	44969187	4.0	G/T	−0.5	MC18IR	PT19DR/MC18DR/AAE/PT18IR/MC19IR	GWAS-CW QTL^**4**^, δ13C-QTL^**6**^
	ss715635422	19	44974132	4.0	T/C	−0.5	MC18IR	PT19DR/MC18DR/AAE/PT18IR	GWAS-CW QTL^**4**^, δ13C-QTL^**6**^
	ss715635432	19	45046164	4.0	T/G	−0.5	MC18IR	PT19DR/MC18DR/AAE/PT18IR	GWAS-CW QTL^**4**^
	ss715635433	19	45062248	4.0	C/T	−0.5	MC18IR	PT19DR/MC18DR/AAE/PT18IR/MC19IR	GWAS-CW QTL^**4**^
	ss715635435	19	45064787	3.7	C/T	−0.5	MC18IR	PT19DR	GWAS-CW QTL^**4**^
	ss715635436	19	45066028	3.7	T/C	−0.5	MC18IR	PT19DR	GWAS-CW QTL^**4**^
	ss715635437	19	45067155	3.6	T/C	−0.5	MC18IR	PT19DR/AAE	GWAS-CW QTL^**4**^
	ss715635439	19	45072668	3.7	C/A	−0.5	MC18IR	PT19DR	GWAS-CW QTL^**4**^
	ss715635442	19	45080661	4.0	C/T	−0.5	MC18IR	PT19DR/MC18DR/AAE/PT18IR/MC19IR	GWAS-CW QTL^**4**^
	ss715635447	19	45099890	4.0	A/G	−0.5	MC18IR	PT19DR/MC18DR/AAE/PT18IR/MC19IR	GWAS-CW QTL^**1, 4**^
	ss715635448	19	45101232	4.0	A/G	−0.5	MC18IR	PT19DR/MC18DR/AAE/PT18IR/MC19IR	GWAS-CW QTL^**1, 4**^
24	ss715639090	20	96607	4.7	G/A	−5.1	CO18IR	CO18DR/PT19DR	GWAS-CW QTL^**4**^
25	ss715636929	20	2106131	4.0	C/T	−0.5	MC18IR	MC18DR/RH18DR	GWAS-CW QTL^**1**^, GWAS-δ18O QTL^**2**^
	ss715636930	20	2109330	4.1	T/G	−0.5	MC18IR	MC18DR/RH18DR	GWAS-CW QTL^**1**^, GWAS-δ18O QTL^**2**^
**Drought (DR)**									
1	ss715580188	1	51097931	4.1	C/A	−2.7	MC18DR		GWAS-CW QTL^**4**^
	ss715580224	1	51398886	6.6	G/A	−9.0	CO18DR	PT19DR	GWAS-CW QTL^**4**^
2	ss715582745	2	4438645	5.8	A/G	−1.1	PT18DR	Ave_IR/PT18IR/MC19IR	GWAS-δ13C QTL^**2**^
	ss715582949	2	4611848	5.2	T/C	2.1	RH18DR	AAE/Ave_IR/PT18IR	GWAS-δ13C QTL^**2**^
3	ss715584310	2	9896652	5.4	T/C	1.7	PT18DR	RH18DR/Ave_IR	GWAS-δ13C QTL^**2**^, GWAS-CT QTL^**3**^
	ss715580895	2	10064424	7.8	C/T	4.5	MC19DR	MC19IR	GWAS-δ13C QTL^**2**^, GWAS-CT QTL^**3**^
4	ss715592981	6	12999522	4.7	T/G	4.4	RH18DR	AAE	GWAS-CW QTL^**4**^
5	ss715593221	6	14321140	8.2	C/T	4.2	CO18DR	AAE/PT18IR	GWAS-CW QTL^**4**^
6	ss715594542	6	46074664	4.5	A/G	0.7	PT18DR		GWAS-CW QTL^**1**^
7	ss715595238	6	50038514	4.1	C/T	−0.2	RH18DR	Ave_DR/AAE	δ13C-QTL^**6**^
8	ss715597447	7	36678744	4.0	C/T	1.2	MC18DR		GWAS-δ13C QTL^**2**^
9	ss715606242	10	3253404	3.7	A/C	0.6	RH18DR	RH18IR/PT18DR/MC19IR	GWAS-CW QTL^**4**^
10	ss715611163	11	7934621	4.0	A/G	−5.1	CO18DR	PT19DR/PT18IR	GWAS-δ13C QTL^**2**^
11	ss715611278	11	9063628	6.1	A/G	−1.0	MC19DR	RH18IR	GWAS-CW QTL^**1**^
12	ss715610265	11	36484015	4.1	C/T	1.8	MC19DR	Ave_DR	GWAS-CT QTL^**3**^, GWAS-CW QTL^**4**^
13	ss715611782	12	2146126	8.4	C/T	2.3	CO18DR		GWAS-CW QTL^**4**^
14	ss715612746	12	37356120	9.5	C/T	−3.4	RH18DR	RH18IR/PT18DR/PT18IR/MC19IR	GWAS-CW QTL^**1**^
15	ss715620072	14	915942	5.5	T/G	−2.4	CO18DR		GWAS-CW QTL^**1**^, Canopy_wilt_1_2^**10**^
16	ss715618057	14	2311158	4.7	G/A	0.8	MC18DR		GWAS-CT QTL^**3**^
17	ss715618585	14	3819249	4.1	A/G	−2.2	RH18DR	MC19IR	GWAS-CT QTL^**3**^
18	ss715623062	15	7718600	8.7	A/G	2.1	CO18DR		GWAS-δ13C QTL^**2**^
19	ss715624261	16	30036170	4.3	A/G	3.0	MC18DR		GWAS-CW QTL^**1**^
20	ss715628231	17	8248213	4.1	G/T	−4.6	MC19DR	PT18IR	GWAS-δ18O QTL^**2**^, δ13C-QTL^**5**^, mqCanopy_wilt_021^**8**^
21	ss715626991	17	36586178	4.7	C/T	−4.4	PT18DR	PT19DR/PT18IR	GWAS-δ13C QTL^**2**^, GWAS-CW QTL^**4**^
22	ss715632594	18	702847	3.8	T/C	0.0	MC19DR	Ave_DR	GWAS-δ13C QTL^**2**^
23	ss715632103	18	59162269	6.0	C/T	−5.4	PT18DR	Ave_IR/Ave_DR/PT18IR/AAE/PT19IR/MC18IR/CO18DR/PT19DR	GWAS-CW QTL^**1**^, δ13C-QTL^**6**^
24	ss715633103	19	1252510	4.0	T/C	1.6	MC18DR	AAE/Ave_DR/PT19DR	GWAS-δ13C QTL^**2**^
25	ss715635396	19	44705794	6.5	T/C	0.7	CO18DR	PT18IR	δ13C-QTL^**6**^
	ss715635416	19	44938831	6.2	G/A	−4.0	RH18DR	PT19DR/MC18IR/PT18IR/AAE	GWAS-CW QTL^**4**^, δ13C-QTL^**6**^
	ss715635458	19	45178132	10.6	T/C	−3.5	CO18DR	PT18IR/PT19DR/AAE	GWAS-CW QTL^**1, 4**^
26	ss715638951	20	46752502	5.3	G/A	4.6	MC19DR	MC18DR/PT19DR	GWAS-CW QTL^**1**^
**Average irrigated (Ave_IR)**									
1	ss715614827	13	28425391	4.3	T/C	−0.1	Ave_IR	MC18IR	GWAS-CW QTL^**4**^
2	ss715614963	13	29130244	3.7	C/T	1.0	Ave_IR		GWAS-δ13C QTL^**2**^
	ss715615031	13	29565886	3.7	G/A	1.1	Ave_IR	CO18IR/PT18IR	GWAS-δ13C QTL^**2**^
3	ss715617562	14	1219464	5.3	A/G	−1.3	Ave_IR	PT18IR/MC19IR	GWAS-CW QTL^**1**^
4	ss715632103	18	59162269	8.5	C/T	−4.0	Ave_IR	PT18DR/Ave_DR/PT18IR/AAE/PT19IR/MC18IR/CO18DR/PT19DR	GWAS-CW QTL^**1**^, δ13C-QTL^**6**^
5	ss715635361	19	44462578	9.0	A/G	−1.3	Ave_IR	PT18IR/MC18IR	δ13C-QTL^**6**^
**Average drought (Ave_DR)**									
1	ss715580947	2	1073084	5.7	C/T	−1.4	Ave_DR	MC18DR/AAE/PT19DR	GWAS-CW QTL^**1**^
2	ss715588382	4	43699749	3.7	A/G	−2.3	Ave_DR	PT19DR	GWAS-CW QTL^**1**^, GWAS-CT QTL^**3**^
3	ss715588984	4	48593967	7.3	T/C	0.9	Ave_DR	AAE/RH18DR/CO18IR/CO18DR/PT18IR	GWAS-CW QTL^**1**^
4	ss715591524	5	37592814	7.5	C/A	1.7	Ave_DR	RH18DR/PT19DR/MC18IR	GWAS-CW QTL^**1**^, δ13C-QTL^**6**^
5	ss715598277	7	4921108	3.6	A/G	−0.8	Ave_DR	AAE/PT19DR	GWAS-δ18O QTL^**2**^
6	ss715599784	8	16250528	6.2	T/G	−2.4	Ave_DR	MC18IR/PT19DR/PT18IR	GWAS-CW QTL^**1**^
7	ss715604779	9	43958999	6.0	A/G	1.6	Ave_DR	RH18DR	GWAS-CW QTL^**1**^
8	ss715632103	18	59162269	4.8	C/T	−4.7	Ave_DR	PT18DR/Ave_IR/PT18IR/AAE/PT19IR/MC18IR/CO18DR/PT19DR	GWAS-CW QTL^**1**^, δ13C-QTL^**6**^
**Average across environments (AAE)**									
1	ss715590862	5	33130272	3.8	G/A	0.9	AAE	PT19IR	GWAS-CW QTL^**1**^
2	ss715598845	7	8518123	5.5	G/T	1.1	AAE	PT19DR/CO18IR	δ13C-QTL^**5**^
3	ss715611329	11	9912187	4.5	G/A	−3.1	AAE	PT18IR/CO18DR	mqCanopy_wilt_020^**8**^
4	ss715632103	18	59162269	3.8	C/T	−5.0	AAE	Ave_IR/PT18DR/Ave_DR/PT18IR/PT19IR/MC18IR/CO18DR/PT19DR	GWAS-CW QTL^**1**^, δ13C-QTL^**6**^

*For each SNP, the following information is reported: locus number, chromosomal position (CHR) in base pairs, –log10(P), allele and allelic effect, environment (ENV) where the SNP was significant, common environments (common ENV) where the SNP was also significant, and quantitative trait loci (QTL) that was coincident from previous studies. Green highlighted areas represent genomic regions in this study that were exactly coincident with Kaler et al. (2017a) genomic regions for CW*.

a *Major/minor alleles of SNP*.

b *Allelic effect: Difference in mean CW between genotypes with major allele and minor allele. Negative sign indicates that major allele is favorable for CW. Positive sign indicates that minor allele is favorable for CW*.

c *Common Env: Indicates that SNP was significant (P ≤ 0.05) in additional environments*.

d *Coincident genomic regions with previous studies: (1) Kaler et al. (2017a) (CW); (2) Kaler et al. (2017b) (C13 and O18); (3) Kaler et al. (2018) (CT); (4) Steketee et al. (2020) (CW); (5) Bazzer et al. (2020a) (C13); (6) Bazzer et al. (2020b) (C13); (7) Bazzer and Purcell (2020) (CT); (8) Hwang et al. (2016) (CW); (9) Abdel Haleem et al. (2012) (CW); (10) Charlson et al. (2009) (CW). CW, canopy wilting; CT, canopy temperature; C13, carbon isotope ratio; O18, oxygen isotope ratio. For QTL naming: genome-wide association (GWAS) followed by trait name means its association mapping QTL (for example, GWAS-O18 QTL). If a GWAS prefix was not included, the QTL was identified from biparental mapping*.

**Table 4:** Significant novel SNPs identified from this study associated with CW for the 12 environments, Columbia (CO18), Maricopa (MC18 and 19), Pine Tree (PT18 and 19), and Rohwer (RH18) under IR and DR treatments, and for CW scores averaged across site-years for irrigated (Ave_IR) and drought (Ave_DR) treatments, and averaged across all environments (AAE) at the significant threshold *P*-value [–Log10 (*P*) ≥ 3.5; *P* ≤ 0.0003].

**Locus**	**SNP_ID**	**CHR**	**Position**	**–Log10 (*P*)**	**Allele^a^**	**Allelic effect^b^**	**Env**	**Common ENV^c^**
**Irrigated (IR)**								
1	ss715579037	1	3318347	9.5	G/A	−5.0	RH18IR	AAE/Ave_IR/PT19DR/Ave_DR/PT18IR/CO18DR
2	ss715582188	2	4076323	4.3	T/C	−3.6	CO18IR	PT19DR/AAE
3	ss715583267	2	4909353	5.2	T/C	0.3	CO18IR	
4	ss715583177	2	48286454	4.3	C/T	−2.4	PT19IR	
5	ss715589211	4	6979721	4.3	A/G	−0.2	RH18IR	AAE
6	ss715599166	8	10308372	3.9	A/G	0.4	PT19IR	
7	ss715601604	8	38203165	9.2	C/T	−3.5	CO18IR	PT19DR
8	ss715603362	9	24238724	3.9	T/C	−0.8	MC18IR	
9	ss715603732	9	35730340	4.7	C/T	−1.2	PT19IR	
10	ss715604821	9	44523441	3.5	G/A	<0.1	CO18IR	
11	ss715609311	11	1617883	3.7	C/T	−0.6	MC18IR	
12	ss715613244	12	6151953	4.5	C/T	1.2	PT19IR	AAE/RH18IR/RH18DR
13	ss715612970	12	39465759	4.9	C/T	0.7	RH18IR	
14	ss715616824	13	5766019	4.1	G/T	1.3	RH18IR	PT18IR
15	ss715614097	13	22446130	7.9	T/C	−1.5	PT19IR	
16	ss715620897	15	15081653	6.0	T/C	−2.2	CO18IR	AAE
17	ss715621830	15	40160334	4.1	G/A	−0.8	MC18IR	
18	ss715621869	15	41427016	4.0	A/G	−0.9	MC18IR	
19	ss715621873	15	41570902	4.0	T/C	−0.8	MC18IR	
20	ss715621877	15	41711821	4.0	A/G	−0.8	MC18IR	
21	ss715623751	16	2320279	5.0	C/T	−0.9	MC18IR	
22	ss715626698	17	311571	8.8	C/A	−6.5	CO18IR	Ave_IR/PT19IR/CO18DR/PT19DR/AAE/PT18IR
23	ss715632608	18	7138173	3.9	C/T	−4.1	PT18IR	RH18DR/PT19DR
24	ss715633673	19	30020611	5.4	C/A	−7.7	CO18IR	CO18DR/AAE/Ave_IR/PT19DR/PT18IR/Ave_DR/MC18IR
25	ss715636922	20	20831971	4.2	A/C	−0.9	MC18IR	PT18DR
	ss715636931	20	21123471	4.2	G/A	−0.9	MC18IR	PT18DR
	ss715636938	20	21317230	4.1	T/C	−0.8	MC18IR	
26	ss715636958	20	22132466	4.1	C/T	−0.8	MC18IR	PT19IR
27	ss715637018	20	23693436	3.5	C/A	−0.8	MC18IR	
	ss715637021	20	23754062	3.8	A/G	−0.9	MC18IR	PT18DR
	ss715637031	20	23941995	3.8	G/A	−0.9	MC18IR	PT18DR
	ss715637033	20	23971966	3.8	A/C	−0.9	MC18IR	PT18DR
	ss715637037	20	23996839	3.8	T/C	−0.9	MC18IR	PT18DR
	ss715637047	20	24131410	3.8	C/T	−0.9	MC18IR	PT18DR
	ss715637052	20	24202089	3.8	A/G	−0.9	MC18IR	PT18DR
	ss715637062	20	24571057	3.8	C/T	−0.9	MC18IR	PT18DR
28	ss715637093	20	25746345	3.8	C/T	−0.9	MC18IR	PT18DR
**Drought (DR)**								
1	ss715578838	1	2675653	4.5	C/T	−3.1	RH18DR	MC18IR/RH18IR/PT18IR
	ss715578860	1	2747136	5.0	A/C	−2.2	MC19DR	PT19DR
2	ss715578432	1	11047155	4.8	T/C	−4.1	MC18DR	MC18IR/PT18IR/PT19DR
3	ss715578452	1	12231584	4.4	G/A	−7.0	PT19DR	PT18IR/MC18IR
4	ss715586000	3	40950687	5.3	G/T	<0.1	CO18DR	
5	ss715586264	3	43838447	4.1	C/T	−6.2	PT19DR	CO18DR/PT18IR
	ss715586270	3	43992877	3.9	A/C	−5.8	PT19DR	PT18IR
	ss715586272	3	44011106	3.7	G/T	−5.5	PT19DR	CO18DR/PT18IR
6	ss715589774	5	1068534	4.1	C/T	−8.8	CO18DR	PT19DR/PT18IR
7	ss715598333	7	5382683	4.1	T/C	1.1	RH18DR	
8	ss715599966	8	17718826	7.9	A/G	1.3	CO18DR	MC19IR
9	ss715601844	8	40735987	3.6	T/C	−1.1	CO18DR	CO18IR/RH18IR
	ss715601899	8	41163228	4.1	C/A	−5.0	CO18DR	Ave_DR/CO18IR
	ss715601932	8	41533120	3.9	A/G	−4.4	CO18DR	AAE/Ave_DR
10	ss715603337	9	22917889	6.4	G/A	−5.4	CO18DR	MC18IR/PT18DR/AAE
11	ss715604653	9	42974503	4.1	G/T	−1.6	RH18DR	Ave_DR
12	ss715604845	9	44799336	5.9	T/C	4.2	MC19DR	CO18IR
13	ss715605772	10	18658872	10.2	A/G	1.2	PT18DR	MC19DR/PT18IR/MC19IR/Ave_IR
14	ss715607829	10	47946637	4.4	C/A	0.2	MC18DR	
15	ss715607984	10	48811357	5.1	G/A	−1.1	PT18DR	
16	ss715609728	11	24299777	4.7	A/G	−3.1	PT18DR	PT18IR
17	ss715611451	12	12635492	5.4	A/G	−3.2	MC18DR	
18	ss715612366	12	34102452	4.2	A/G	0.5	PT18DR	PT18IR
19	ss715617261	13	852961	4.7	T/C	−2.9	MC19DR	
20	ss715616860	13	5993892	4.1	T/C	−0.6	PT18DR	CO18IR/MC19DR/AAE
	ss715616861	13	5994977	4.1	A/G	−0.6	PT18DR	CO18IR/MC19DR/AAE
21	ss715614911	13	28880849	4.6	C/T	2.2	CO18DR	MC18R/CO18IR/PT18IR/RH18IR
22	ss715619422	14	48312276	5.0	T/G	−0.9	PT18DR	MC19DR
	ss715619486	14	48836596	3.9	G/A	−2.4	PT18DR	PT19DR/PT18IR
23	ss715622900	15	6272006	3.7	T/C	0.1	RH18DR	Ave_DR/CO18DR
24	ss715625245	16	5866453	5.7	A/G	2.2	MC19DR	Ave_IR/PT19DR
25	ss715623895	16	27521057	5.1	T/G	−0.1	MC18DR	
26	ss715623938	16	28238331	4.4	G/A	2.7	MC19DR	PT18DR/CO18DR
27	ss715626698	17	311571	3.6	C/A	−9.1	CO18DR	Ave_IR/PT19IR/CO18IR/PT19DR/AAE/PT18IR
28	ss715626021	17	13252195	3.8	G/A	1.1	MC18DR	
29	ss715626252	17	15616539	4.6	C/T	3.7	MC18DR	MC19DR
30	ss715630859	18	48314296	5.2	C/T	−3.4	PT18DR	PT18IR/PT19DR
31	ss715631739	18	56124763	5.5	T/C	3.3	MC18DR	PT19DR/Ave_DR
32	ss715632215	18	59814051	4.9	T/C	4.3	RH18DR	MC18DR/PT19DR
	ss715633191	19	1502707	4.1	C/T	3.1	PT19DR	
33	ss715636101	19	5205785	4.9	T/C	0.1	PT18DR	MC18IR/CO18IR
34	ss715633673	19	30020611	5.3	C/A	−12.6	CO18DR	CO18IR/AAE/Ave_IR/PT19DR/PT18IR/Ave_DR/MC18IR
35	ss715634898	19	39686084	6.1	C/T	−0.4	MC18DR	
**Average irrigated (Ave_IR)**								
1	ss715579037	1	3318347	4.3	G/A	−3.3	Ave_IR	RH18IR/AAE/PT19DR/Ave_DR/PT18IR/CO18DR
2	ss715583262	2	49079505	6.7	T/C	1.2	Ave_IR	PT18IR
3	ss715586436	3	45668212	5.4	G/A	0.9	Ave_IR	CO18IR/CO18DR/AAE
4	ss715587848	4	36908862	3.6	T/C	0.3	Ave_IR	
5	ss715597242	7	34279212	4.6	C/T	−1.1	Ave_IR	PT18IR/Ave_DR
6	ss715614457	13	26279705	4.0	G/A	0.1	Ave_IR	PT18DR
7	ss715625320	16	6483232	3.8	C/T	0.0	Ave_IR	PT19IR
8	ss715626698	17	311571	6.4	C/A	−3.8	Ave_IR	CO18IR/PT19IR/CO18DR/PT19DR/AAE/PT18IR
9	ss715633673	19	30020611	3.6	C/A	−4.5	Ave_IR	CO18IR/CO18DR/AAE/PT19DR/PT18IR/Ave_DR/MC18IR
**Average drought (Ave_DR)**								
1	ss715585976	3	40874888	8.0	C/T	−1.8	Ave_DR	AAE/PT19DR/Ave_IR/RH18IR
2	ss715597294	7	35103803	4.1	G/A	1.7	Ave_DR	AAE
3	ss715601736	8	40012659	8.6	C/T	−2.2	Ave_DR	PT19DR
4	ss715603468	9	2799750	3.7	T/G	0.9	Ave_DR	
5	ss715631039	18	49432890	3.5	C/T	−4.8	Ave_DR	PT19DR/RH18IR/MC18DR/CO18IR
**Average across environments (AAE)**								
1	ss715579037	1	3318347	6.9	G/A	−5.0	AAE	RH18IR/Ave_IR/PT19DR/Ave_DR/PT18IR/CO18DR
2	ss715585976	3	40874888	6.1	C/T	−1.6	AAE	Ave_DR/PT19DR/Ave_IR/RH18IR
3	ss715597294	7	35103803	4.2	G/A	1.2	AAE	Ave_DR
4	ss715604906	9	4542201	4.3	T/G	1.6	AAE	Ave_DR/PT19IR
5	ss715603638	9	32987730	4.9	G/A	1.7	AAE	Ave_IR/Ave_DR/MC19DR/MC18DR
6	ss715613260	12	6268063	6.2	A/G	−0.6	AAE	PT19DR
7	ss715633673	19	30020611	5.2	C/A	−5.9	AAE	CO18IR/CO18DR/Ave_IR/PT19DR/PT18IR/Ave_DR/MC18IR

*For each SNP, the following information is reported: locus number, CHR in base pairs, –log10(P), allele and allelic effect, ENV where the SNP was significant, and common ENV where the SNP was also significant*.

a *Major/minor alleles of SNP*.

b *Allelic effect: Difference in mean CW between genotypes with major allele and minor allele. Negative sign indicates that major allele is favorable for CW. Positive sign indicates that minor allele is favorable for CW*.

c *Common Env: Indicates that SNP was significant (P ≤ 0.05) in additional environments*.

Out of 87 significant coincident SNPs that we confirmed from previous studies (Table 3), 38 SNPs that likely tagged 25 loci were from the IR treatment in individual environments, 31 SNPs that likely tagged 26 loci were from the DR treatment in individual environments, six SNPs that likely tagged five loci were based on the IR treatment averaged over site-years, eight SNPs that likely tagged eight loci were based on the DR treatment averaged over site-years, and four SNPs that likely tagged four loci were from values averaged across all the environments. Of the 38 significant SNPs from the IR treatment, 31 were present in at least two environments. Of the 31 significant SNPs from the DR treatment, 23 were present in at least two environments.

Four significant SNPs (ss715606242, ss715611329, ss715612746, and ss715632103) on chromosomes Gm10, Gm11, Gm12, and Gm18, respectively, were common between IR and DR treatments, averaged IR and DR treatments, and averaged across all environments (Table 3). Two genomic regions had the exact same markers and positions that were identified by Kaler et al. (2017a). These two SNPs were found on Gm08 (ss715599784) and Gm18 (ss715632103) and had large allelic effects between −2.4 and −5.4 (highlighted area in Table 3). The allelic effect ranged from −5.1 to 2.3 for the 38 SNPs identified for the IR treatment among environments, −9 to 4.6 for the 31 SNPs identified for the DR treatment among environments, −4 to 1.1 for the six SNPs found when averaged over irrigated site-years, −4.7 to 1.7 for the eight SNPs found when averaged over DR site-years, and −5 to 1.1 for the four SNPs found when averaged across all environments.

The SNPs that were not coincident with previous studies were considered novel loci (Table 4). Of 101 novel SNPs, 37 SNPs likely tagged 28 loci from the IR treatment in individual environments, 43 SNPs likely tagged 35 loci from the DR treatment in individual environments, 9 SNPs likely tagged 9 loci when averaged over the IR treatment, 5 SNPs likely tagged 5 loci when averaged over the DR treatment, and 7 SNPs likely tagged 7 loci when averaged across all environments. Of the 37 significant SNPs under the IR treatment, 21 SNPs were present in at least two environments. Out of the 43 significant SNPs from the DR treatment, 33 SNPs were present in at least two environments. Five significant SNPs (ss715579037, ss715585976, ss715597294, ss715626698, and ss715633673) on Gm01, Gm03, Gm07, Gm17, and Gm19, respectively, were common for the IR and DR treatments, averaged values by site-year, and averaged values across all environments.

### Genomic Estimated Breeding Values (GEBVs) and Prediction Accuracy

Averaged CW scores of the 373 accessions from Kaler et al. (2017a) combined with the 153 accessions from Steketee et al. (2020) were used as a training set for genomic prediction of the 100 new accessions used in this study. For three of the six IR site-years, there was a significant positive correlation (*P* ≤ 0.05) between GEBVs and observed CW that ranged from *r* = 0.26 (PT18IR) to *r* = 0.49 (PT19IR) (Table 5). For the DR site-years, four of the six DR site-years had positive significant correlations between GEBVs and observed wilting that ranged from *r* = 0.2 (RH18DR) to *r* = 0.5 (MC18DR). GEBVs averaged across the IR (*r* = *0.4*5) and DR treatments (*r* = 0.43) and averaged across all environments (*r*= 0.45) also showed significant positive correlations.

**Table 5:** Prediction accuracy (correlation) of genomic estimated breeding values (GEBVs) with observed CW scores for the 12 environments for the 100 new accessions used in this study.

**Site year**	**Treatment**	**Correlation**
Columbia/2018	Irrigated	0.09
Maricopa/2018	Irrigated	0.10
Maricopa/2019	Irrigated	0.18
Pine Tree/2018	Irrigated	0.26*
Pine Tree/2019	Irrigated	0.49*
Rohwer/2018	Irrigated	0.46*
Columbia/2018	Drought	0.19
Maricopa/2018	Drought	0.50*
Maricopa/2019	Drought	0.49*
Pine Tree/2018	Drought	0.18
Pine Tree/2019	Drought	0.26*
Rohwer 2018	Drought	0.20*
Averaged	Irrigated	0.45*
Averaged	Drought	0.43*
Averaged	averaged	0.45*

*The training set for the genomic prediction of the 100 new accessions used in this study was determined from the averaged CW of the 373 and the 153 accessions reported by Kaler et al. (2017a) and Steketee et al. (2020), respectively*.

*Significant at P = <0.05.

In a second scenario, averaged CW scores of the 100 new accessions from this study combined with the 153 accessions from Steketee et al. (2020) were used as a training set for genomic prediction of the 373 accessions reported by Kaler et al. (2017a). There were significant positive correlations (*P* ≤ 0.05) between observed CW and GEBVs for individual environments that ranged from *r* = 0.2 (Pine Tree 16) to *r* = 0.39 (Salina 16), and when averaged across environments (*r* = 0.37) (Table 6).

**Table 6:** Prediction accuracy (correlation) of GEBVs with observed CW scores for the four environments reported by Kaler et al. (2017a).

**Location/Year**	**Correlation**
Pine Tree/2016	0.20*
Rohwer/2016	0.27*
Salina/2015	0.26*
Salina/2016	0.39*
Averaged across environments	0.37*

*The training set for the genomic prediction of the 373 accessions reported by Kaler et al. (2017a) was determined from the averaged CW scores of the 100 new accessions of the current study and of the 153 accessions reported by Steketee et al. (2020)*.

*Significant at P = <0.05.

In a third scenario, averaged CW scores of the 100 new accessions from this study combined with the 373 accessions reported by Kaler et al. (2017a) were used as a training set for genomic prediction of the 153 accessions reported by Steketee et al. (2020). There were significant positive correlations (*P* ≤ 0.05) between observed CW and GEBVs for individual environments that ranged from *r* = 0.35 (Salina 15) to *r* = 0.46 (Salina 16), and when averaged across environments (*r* = 0.5) (Table 7).

**Table 7:** Prediction accuracy (correlation) of GEBVs with observed CW scores in four environments reported by Steketee et al. (2020).

**Location/Year**	**Correlation**
Athens/2015	0.38*
Athens/2016	0.45*
Salina/2015	0.35*
Salina/2016	0.46*
Averaged across environments	0.50*

*The training set for the prediction of the 153 accessions reported by Steketee et al. (2020) was determined from the averaged CW scores of the 100 new accessions of the current study and of the 373 accessions reported by Kaler et al. (2017a)*.

*Significant at the P = <0.05.

### Predicting Canopy Wilting for Soybean Germplasm Using GEBVs

Averaged CW scores of the 100 new accessions from this study combined with the 373 accessions reported by Kaler et al. (2017a) and the 153 accessions reported by Steketee et al. (2020) were used as a training set for the genomic prediction of CW for the 19,648 soybean accessions reported by Song et al. (2015). A wide range of predicted CW scores from <15 to more than 31 was observed among the accessions (Supplementary Figure 4). For each MG, the 10 genotypes with the lowest predicted scores and the 10 genotypes with the highest predicted scores are presented in Supplementary Table 5. GEBVs for the slowest wilting genotypes among MGs ranged from 9 to 14, and GEBVs for the fastest wilting genotypes among MGs ranged from 16 to 33. The MG with the greatest range in GEBVs for wilting was MG VI (9 to 33), and the MG with the least range in GEBVs for wilting was MG X (9 to 22) (data not shown).

### Candidate Gene Identification

Of the 87 coincident SNPs and the 101 novel SNPs associated with CW in this study, 87 genes from the coincident SNPs and 96 genes from the novel SNPs were identified within ±10 kb in the euchromatic region and ±100 kb in the heterochromatic region using the *G. max* genome assembly version Glyma.Wm82.a1.v1.1 in SoyBase (www.soybase.org) (Schmutz et al., 2010). The annotations of the biological processes, molecular functions, and cellular components of these genes are reported in Supplementary Table 6 for coincident SNPs and Supplementary Table 7 for novel SNPs. Based on biological functions, several genes were associated with drought-related responses, such as abscisic acid, water, root, leaf senescence, jasmonic acid, heat acclimation, stomata, and salicylic acid (Schulze, 1986; Jackson et al., 2000; Schmutz et al., 2010; Jarzyniak and Jasinski, 2014; Khan et al., 2015; Sah et al., 2016).

## Discussion

This study was conducted to confirm loci previously reported and identify novel loci associated with CW by association mapping. There was wide phenotypic variation in CW, which is important for dissecting complex traits through association mapping (McCarthy et al., 2008). In comparison with slow-wilting checks, on one extreme, PI407927B had significantly lower (*P* <0.05) CW scores under both IR and DR treatments. At the other extreme, PI507407 and PI507408 had wilting scores significantly (*P* <0.05) greater than those of fast-wilting checks under both the IR and DR treatments (Supplementary Table 3). We also predicted slower and faster wilting accessions from the USDA Soybean Germplasm Collection (Supplementary Table 5) using GEBVs. These slow-wilting genotypes represent new genetic resources for providing breeders with favorable slow-wilting alleles.

This study showed significant (*P* <0.001) positive correlations (*r* = 0.8) for CW between the IR and DR treatments, and moderate to high heritability (39% ≤ *H*^2^ ≤ 84%), indicating that CW was relatively stable across the environments. Similar results of correlations and heritability were reported in previous mapping studies for CW (Charlson et al., 2009; Abdel Haleem et al., 2012; Hwang et al., 2015; Kaler et al., 2017a; Steketee et al., 2020).

Research studies over the past 12 years have identified numerous QTLs from the association and linkage mapping studies that were associated with CW (Charlson et al., 2009; Abdel Haleem et al., 2012; Hwang et al., 2015, 2016; Kaler et al., 2017a; Steketee et al., 2020), canopy temperature (Kaler et al., 2018; Bazzer and Purcell, 2020), and C13 and O18 isotope ratios (Kaler et al., 2017b; Bazzer et al., 2020a,b). This study confirmed 87 SNPs that likely tagged 68 loci as coincident genomic regions from previous studies on CW (Charlson et al., 2009; Abdel Haleem et al., 2012; Hwang et al., 2015, 2016; Kaler et al., 2017a; Steketee et al., 2020), canopy temperature (Kaler et al., 2018; Bazzer and Purcell, 2020), and C13 and O18 isotope ratios (Kaler et al., 2017b; Bazzer et al., 2020a,b).

It is counter-intuitive that wilting was rated under water-replete conditions in the IR treatment. Except for MC19, however, there were highly significant (*P* ≤ 0.001) correlations between the IR and DR treatments within a site-year and between the Ave_IR and Ave_DR ratings (*r* = 0.8, Supplementary Table 3). Of the 75 SNPs identified in individual IR environments (Tables 3, 4), 42 of these same SNPs were also found in individual DR environments and 33 were unique to the IR environments. The discovery of wilting QTLs specific for the IR environments may reflect genomic regions that are responsive to the early stages of drought.

Out of the 87 coincident SNPs found in this study, 42 likely tagged 31 loci previously associated with only CW (Charlson et al., 2009; Abdel Haleem et al., 2012; Hwang et al., 2016; Kaler et al., 2017a; Steketee et al., 2020) and 45 likely tagged 37 loci previously identified with other drought-related traits (canopy temperature, and C13 and O18 ratios) (Table 3 and Figure 2). The genomic regions that were consistent across MGs (MGIV from this study and Kaler et al., 2017a,b, 2018; and MGVI–VIII from Steketee et al., 2020) and across biparental populations, and different environments show particular promise as selection targets for improving CW under stress. In particular, SNP_ID ss715632103 on Gm18 (59162269 bp) and SNP_ID ss715599784 on Gm08 (16250528 bp) were identical to SNPs previously associated with CW (Kaler et al., 2017a). The genomic regions found in common between this and previous mapping studies may be an important resource in genomic selection studies to improve drought tolerance in soybean. Apart from coincident SNPs, this study also identified 101 novel SNPs that tagged 84 loci associated with CW that could be additional resources for the improvement of the CW in soybean.

Genomic selection was originally proposed by Meuwissen et al. (2001), and simulations have demonstrated that it is far more effective and efficient than marker-assisted selection for polygenic traits (Bernardo and Yu, 2007; Jannink et al., 2010). Both simulation and empirical studies have repeatedly shown that genomic selection performs as well as, and frequently better than, phenotypic selection (Wong and Bernardo, 2008; Matei et al., 2018; Voss-Fels et al., 2019). The breeding community has concluded that genomic selection has the potential to decrease overall costs and potentially allow more cycles of selection per unit time, as compared with phenotypic selection (Wong and Bernardo, 2008; Matei et al., 2018; Voss-Fels et al., 2019).

We determined the ability of GEBVs using different scenarios of training and testing populations from this and previous studies (Kaler et al., 2017a; Steketee et al., 2020) to predict CW phenotypes of unknown genotypes. In general, there was significant positive prediction accuracy between observed CW and GEBVs. Although the accuracy of the predictions was somewhat low (average irrigated 0.45 and average drought 0.43; Table 5), the heritability for the traits was relatively high when the Maricopa location was excluded (ranging from 0.62 to 0.86, Table 1), and correlations between locations were relatively high (excluding Maricopa; Supplementary Table 3). Based on these results, we anticipate genomic selection will permit more rapid progress toward the release of soybean cultivars with improved tolerance to water limitation and/or higher water-use efficiency than marker-assisted selection, phenotypic selection, or the most common strategy: evaluating breeding populations only in high-yielding, often irrigated, environments.

Out of 188 significant SNPs, 183 candidate genes were identified (87 from coincident SNPs; Supplementary Table 6 and 96 from novel SNPs; Supplementary Table 7) in this study within ±10 kb or ±100kb in euchromatic and heterochromatic regions, respectively, of associated SNPs that had biological functions associated with stress responses or water transport. Among 183 candidate genes identified, 57 SNPs were present within genes that code for proteins having biological functions involved with plant stress responses. These genes may be directly or indirectly associated with transpiration or water conservation. Supplementary Tables 6, 7 provide information on these candidate genes and their associated functions in water transportation, abscisic acid stimulus, and root development (Schmutz et al., 2010).

## Conclusions

This study confirmed 31 slow-wilting loci identified previously by association mapping (Kaler et al., 2017a; Steketee et al., 2020) and linkage mapping (Charlson et al., 2009; Abdel Haleem et al., 2012; Hwang et al., 2016). Similarly, we found 37 CW loci that overlapped with loci for other drought-related traits (C13 ratio, O18 ratio, and canopy temperature). This study also identified 84 novel loci associated with CW using a panel of 200 diverse MG IV soybean accessions. There were 183 candidate genes within ±10 kb (euchromatic region) or ± 100 kb (heterochromatic region) of CW SNPs that were associated with stress responses. GEBVs from this study and previous research studies were used to identify genotypes from all the 13 MGs in the USDA Soybean Germplasm Collection that were extremes for slow or fast wilting. Favorable alleles from confirmed genomic regions and the identification of additional slow-wilting genotypes may be important new resources for improving drought tolerance in soybean.

## Data Availability Statement

The datasets presented in this study can be found in online repositories. The names of the repository/repositories and accession number(s) can be found in the article/Supplementary Material.

## Author Contributions

SC and AK wrote initial drafts of the manuscript. SC, AK, and JG collected genomic information and analyzed data. LP coordinated and supervised the project. All authors were involved in planning, executing, collecting data from field experiments, and read and approved the final manuscript.

## Conflict of Interest

The authors declare that the research was conducted in the absence of any commercial or financial relationships that could be construed as a potential conflict of interest.

## Supplementary Material

Supplementary Figure 1 Manhattan plots of –Log10 (P) vs. chromosomal position of significant SNP associations for canopy wilting for six irrigated treatments: **(a)** Columbia (CO18IR), **(b)** Maricopa (MC18IR), **(c)** Maricopa (MC19IR), **(d)** Pine Tree (PT18IR), **(e)** Pine Tree (PT19IR), **(f)** Rohwer (RH18IR), and **(g)** averaged over site-years for irrigated treatments (Ave_IR). The red dotted line in each panel represents the association threshold [–Log10 (*P*) ≥ 3.5; *P* ≤ 0.0003].

Supplementary Figure 2 Manhattan plots of –Log10 (*P*) vs. chromosomal position of significant SNP associations of canopy wilting for six drought treatments: **(a)** Columbia (CO18DR), **(b)** Maricopa (MC18DR), **(c)** Maricopa (MC19DR), **(d)** Pine Tree (PT18DR), **(e)** Pine Tree (PT19DR), **(f)** Rohwer (RH18DR), and **(g)** averaged over-site years for drought treatments (Ave_DR). The red dotted line in each panel represents the association threshold [–Log10 (*P*) ≥ 3.5; *P* ≤ 0.0003].

Supplementary Figure 3 Manhattan plot of –Log10 (*P*) vs. chromosomal position of significant SNP associations of canopy wilting when averaged across all environments (AAE). The red dotted line represents the association threshold [–Log10 (*P*) ≥ 3.5; *P* ≤ 0.0003].

Supplementary Figure 4 Frequency distribution for predicted canopy wilting scores for 19,648 soybean accessions in the USDA Soybean Germplasm Collection for maturity groups (MGs) 000 through X. Canopy wilting scores were predicted using genomic estimated breeding values that used training sets from the current research, Kaler et al. (2017a), and Steketee et al. (2020).

Supplementary Table 1 Planting dates, wilting rating dates, weather data, number of irrigations, and potential evapotranspiration rate for the drought treatment at six site years. ^a^Cumulative potential evapotranspiration between emergence and last rating date.

Supplementary Table 2 Canopy wilting BLUP values used for GWAS analysis for twelve environments, Columbia (CO18), Maricopa (MC18 and 19), Pine Tree (PT18 and 19), Rohwer (RH18) under irrigated (IR) and drought (DR) treatments, and averaged across irrigated (Ave_IR) and drought (Ave_DR) treatments, and averaged across all environments (AAE).

Supplementary Table 3 Accessions identified as wilting slower or faster than slow-wilting (PI416937 and PI471938) and fast-wilting (A5959 and 08705_16) checks when averaged over irrigated (Ave_IR) and drought (Ave_DR) treatments. ^a^Standard deviation values from analysis of variance for Ave_IR and Ave_DR were 6.8 and 8.9, respectively.

Supplementary Table 4 Correlations for canopy wilting (*n* = 206) for Columbia (CO18), Maricopa (MC18 and 19), Pine Tree (PT18 and 19), Rohwer (RH18) irrigated (IR) and drought (DR) treatments, averaged over irrigated (Ave_IR) and averaged drought (Ave_DR) treatments, and averaged across all environments (AAE).

Supplementary Table 5 Predicted slowest and fastest wilting accessions of maturity group (MG) 000 through X using genomic estimated breeding values (GEBVs) from the USDA Soybean Germplasm Collection (Song et al., 2015). The training set for the prediction of 19,648 accessions was determined using the averaged canopy wilting (CW) scores of 100 new accessions in the present study, 373 accessions reported by Kaler et al. (2017a), and of the 153 accessions reported by the Steketee et al. (2020).

Supplementary Table 6 List of significant coincident SNPs associated with canopy wilting and their potential candidate genes based on 87 identified SNPs from twelve environments for the irrigated and drought treatments, averaged over irrigated treatments (Ave_IR), average over drought treatments (Ave_DR), and averaged across all environments (AAE).

Supplementary Table 7 Significant novel SNPs associated with canopy wilting and their potential candidate genes based on 101 identified SNPs from twelve environments under irrigated and drought treatments, averaged across irrigated treatments (Ave_IR), averaged across drought treatments (Ave_DR), and averaged across all environments (AAE).
