# Supplementary figures and images for: Identification and Confirmation of Loci Associated With Canopy Wilting in Soybean Using Genome-Wide Association Mapping
Source: Front Plant Sci. 2021 Jul 14;12:698116. doi: 10.3389/fpls.2021.698116 (PMC8317169; doi:10.3389/fpls.2021.698116)

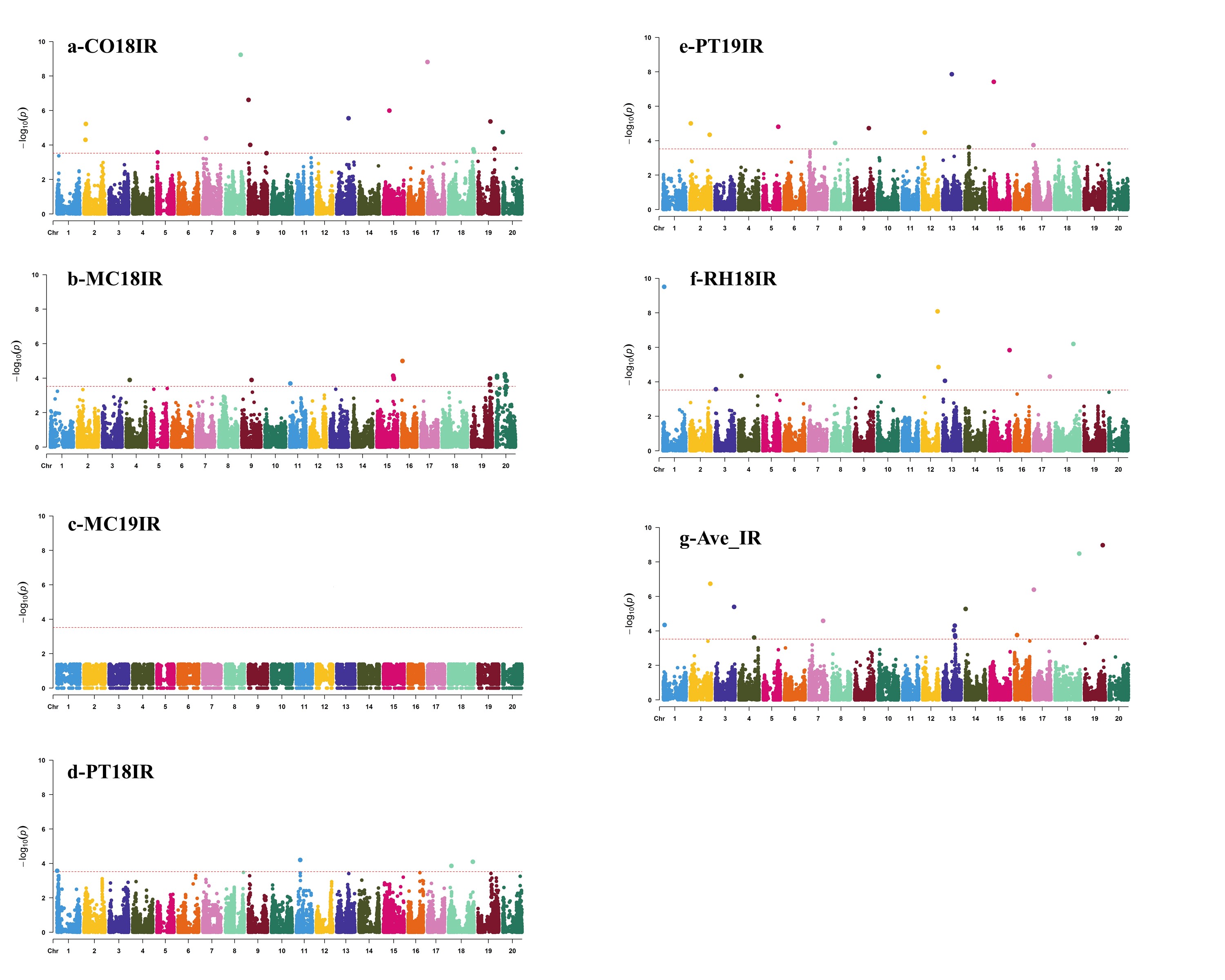

Supplement: Supplementary Figure 1 — Manhattan plots of –Log10 (P) vs. chromosomal position of significant SNP associations for canopy wilting for six irrigated treatments: (a) Columbia (CO18IR), (b) Maricopa (MC18IR), (c) Maricopa (MC19IR), (d) Pine Tree (PT18IR), (e) Pine Tree (PT19IR), (f) Rohwer (RH18IR), and (g) averaged over site-years for irrigated treatments (Ave_IR). The red dotted line in each panel represents the association threshold [–Log10 (P) ≥ 3.5; P ≤ 0.0003]. [file Image_1.JPEG]

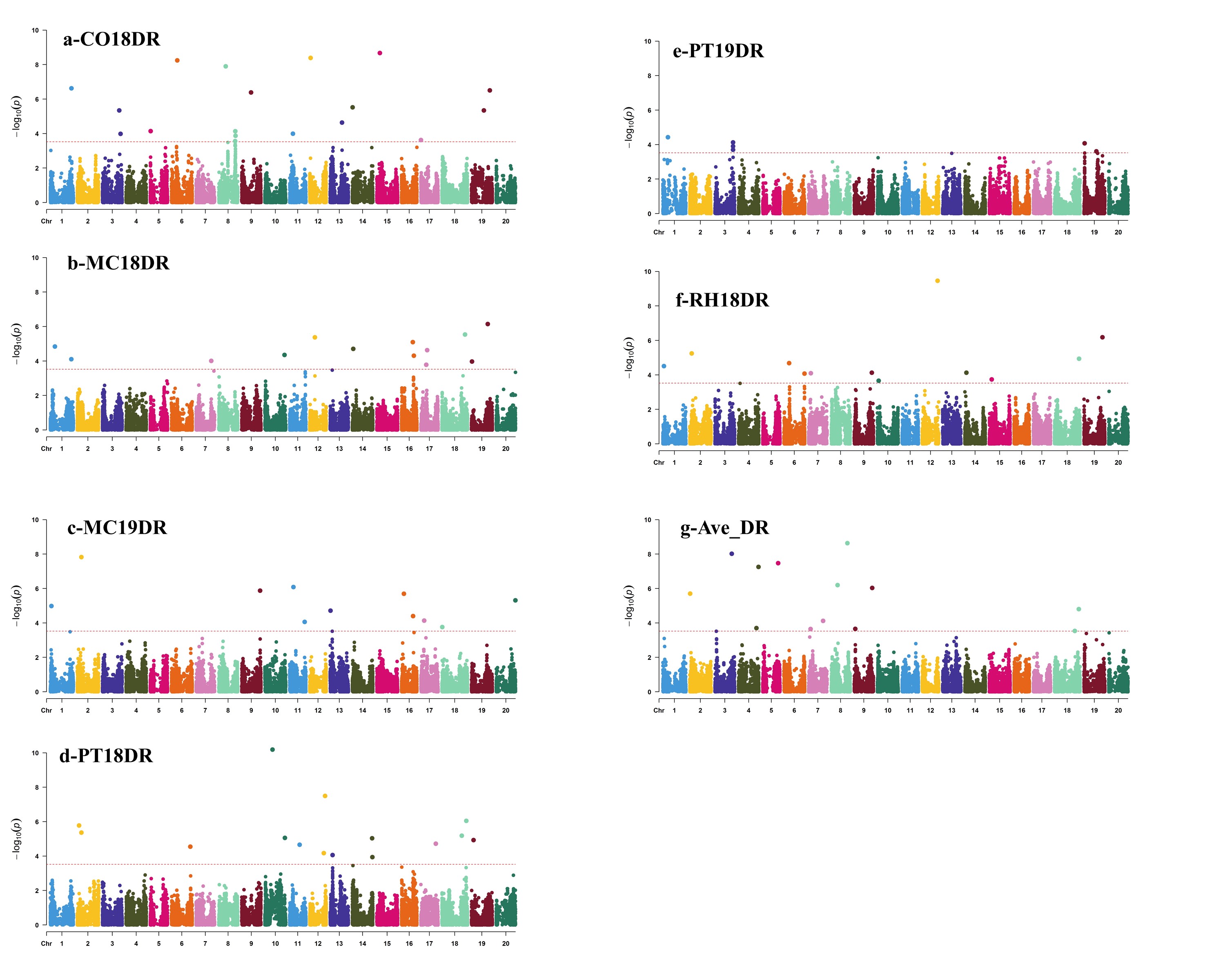

Supplement: Supplementary Figure 2 — Manhattan plots of –Log10 (P) vs. chromosomal position of significant SNP associations of canopy wilting for six drought treatments: (a) Columbia (CO18DR), (b) Maricopa (MC18DR), (c) Maricopa (MC19DR), (d) Pine Tree (PT18DR), (e) Pine Tree (PT19DR), (f) Rohwer (RH18DR), and (g) averaged over-site years for drought treatments (Ave_DR). The red dotted line in each panel represents the association threshold [–Log10 (P) ≥ 3.5; P ≤ 0.0003]. [file Image_2.JPEG]

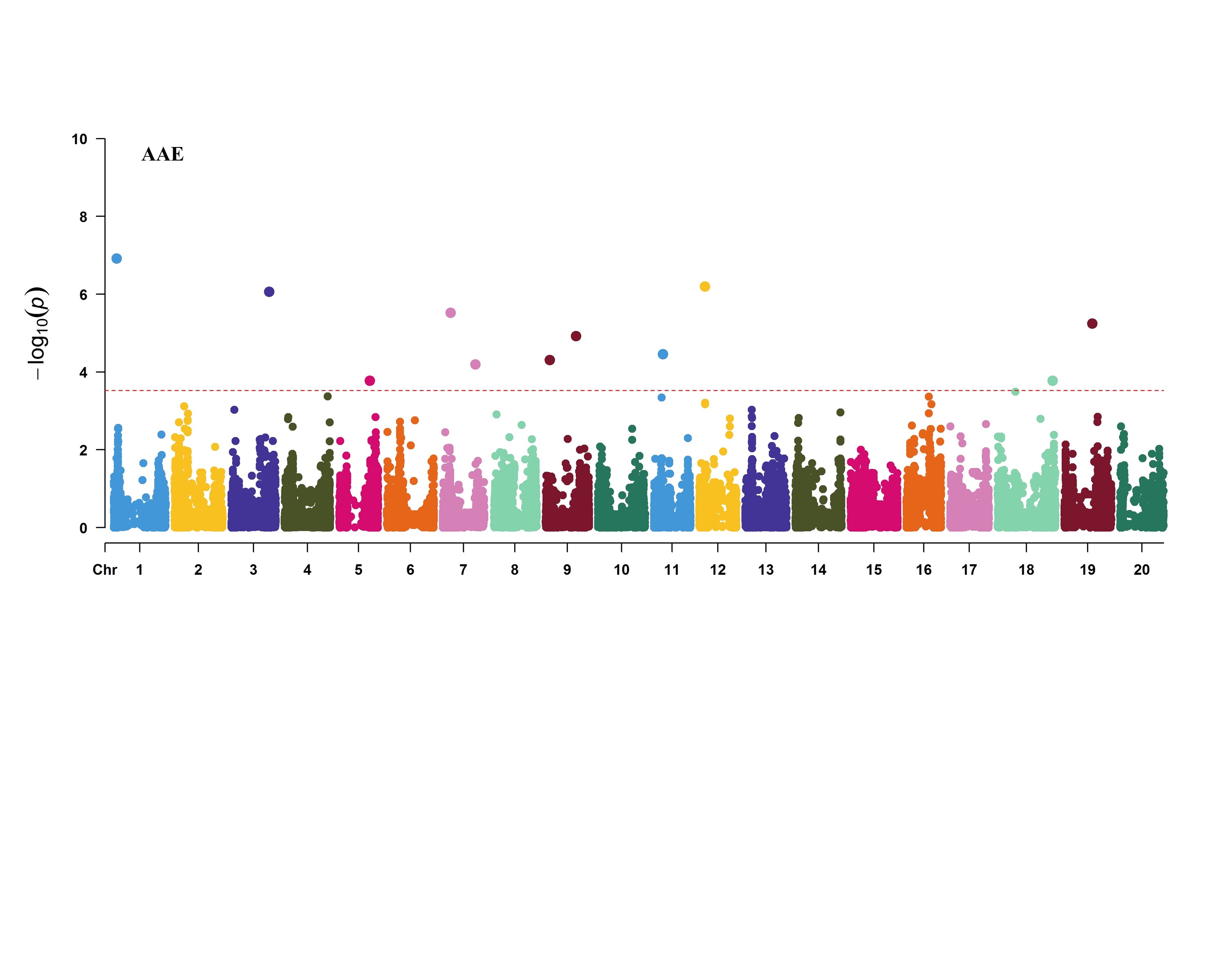

Supplement: Supplementary Figure 3 — Manhattan plot of –Log10 (P) vs. chromosomal position of significant SNP associations of canopy wilting when averaged across all environments (AAE). The red dotted line represents the association threshold [–Log10 (P) ≥ 3.5; P ≤ 0.0003]. [file Image_3.JPEG]

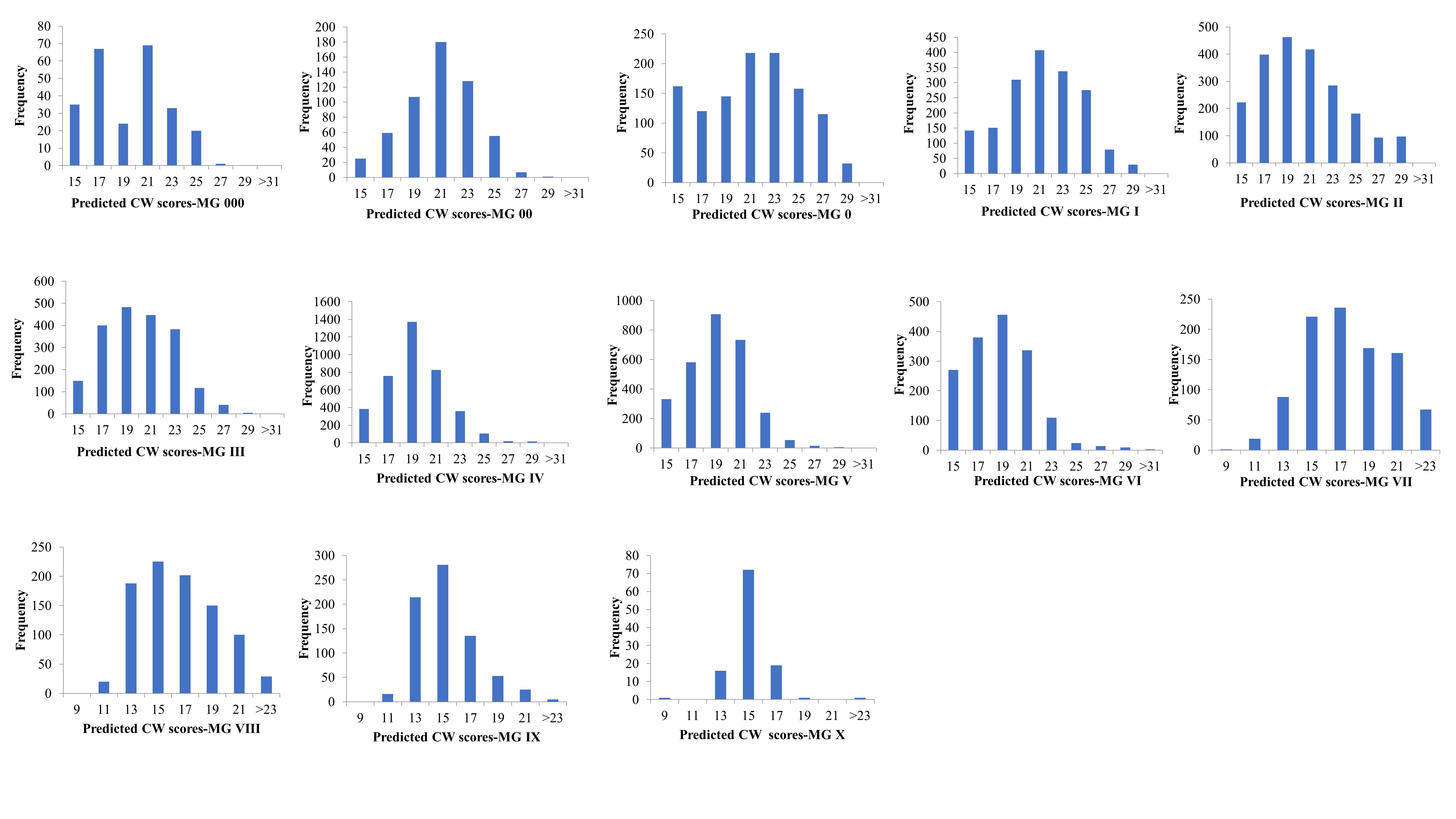

Supplement: Supplementary Figure 4 — Frequency distribution for predicted canopy wilting scores for 19,648 soybean accessions in the USDA Soybean Germplasm Collection for maturity groups (MGs) 000 through X. Canopy wilting scores were predicted using genomic estimated breeding values that used training sets from the current research, Kaler et al. (2017a), and Steketee et al. (2020). [file Image_4.JPEG]
