# Supplementary material for: Identification and Confirmation of Loci Associated With Canopy Wilting in Soybean Using Genome-Wide Association Mapping
Source: Front Plant Sci. 2021 Jul 14;12:698116. doi: 10.3389/fpls.2021.698116 (PMC8317169; doi:10.3389/fpls.2021.698116)
Supplement: Supplementary Table 1 — Planting dates, wilting rating dates, weather data, number of irrigations, and potential evapotranspiration rate for the drought treatment at six site years. aCumulative potential evapotranspiration between emergence and last rating date. [file Data_Sheet_1.docx]

|  | Rohwer  2018 | Pine Tree  2018 | Pine Tree  2019 | Columbia  2018 | Maricopa  2018 | Maricopa  2019 |
| --- | --- | --- | --- | --- | --- | --- |
| Planting date | 31-May | 7-Jun | 30-May | 7-May | 17-May | 17-May |
| Wilting rating date | 24-Jul | 6-Aug | 20-Aug | 1, 3, 6, 13-Aug | 17, 24-Jul | 23, 26-July; 1, 5-Aug |
| Average maximum temperature (°C) | 32 | 33 | 32 | 30 | 40 | 39 |
| Average minimum temperature (°C) | 23 | 22 | 22 | 18 | 22 | 21 |
| Total Precipitation between planting and rating (mm) | 101 | 193 | 376 | 234 | 5 | 33 |
| Days between rain/irrigation and rating | 3 | 6 | 6 | 3, 2, 3, 7 | 14, 21 | 18, 21, 27 and 31 |
| Number of irrigations | 3 | 1 | 0 | 4 | 3 | 3 |
| Cumulative potential evapotranspiration (mm)^a^ | 151 | 187 | 249 | 309 | 318 | 385 |

**Table S1.** Planting dates, wilting rating dates, weather data, number of irrigations, and potential evapotranspiration rate for the drought
treatment at six site years.

^a^Cumulative potential evapotranspiration between emergence and last rating date.

| **Table S2.** Canopy wilting BLUP values used for GWAS analysis for twelve environments, Columbia (CO18), Maricopa (MC18 &19), Pine Tree (PT18 & 19), Rohwer (RH18) under irrigated (IR) and drought (DR) treatments, and averaged across irrigated (Ave_IR) and drought (Ave_DR) treatments, and averaged across all environments (AAE). | | | | | | | | | | | | | | | |
| --- | --- | --- | --- | --- | --- | --- | --- | --- | --- | --- | --- | --- | --- | --- | --- |
| Accession | CO18IR | MC18IR | MC19IR | PT18IR | PT19IR | RH18IR | CO18NI | MC18NI | MC19NI | PT18NI | PT19NI | RH18NI | Average_IR | Average_NI | AAE |
| FC31630 | 12.6 | 21.8 | 20.1 | 21.3 | 19.8 | 14.8 | 17.2 | 24.0 | 21.2 | 19.6 | 26.9 | 22.0 | 18.1 | 21.9 | 20.2 |
| PI157396 | 7.1 | 21.3 | 20.1 | 18.8 | 14.6 | 11.4 | NA | 26.5 | 31.4 | 19.8 | 22.0 | 17.3 | 14.3 | 22.2 | 18.3 |
| PI157452 | 7.8 | 23.8 | 20.1 | 20.1 | 14.6 | 14.1 | 11.3 | 36.5 | 27.5 | 16.5 | 22.0 | 18.0 | 16.4 | 21.9 | 19.4 |
| PI157471 | 10.4 | 21.4 | 20.1 | 19.0 | 16.9 | 12.9 | 16.2 | 32.7 | 33.5 | 18.1 | 23.2 | 20.9 | 16.0 | 24.1 | 20.4 |
| PI171433 | 11.0 | 21.1 | 20.1 | 17.9 | 15.8 | 9.9 | 23.7 | 29.6 | 22.6 | 15.4 | 25.7 | 16.1 | 14.9 | 22.2 | 18.7 |
| PI226591 | 7.2 | 21.8 | 20.1 | 17.9 | 22.9 | 16.1 | 14.9 | 25.9 | 30.3 | 19.6 | 23.2 | 20.1 | 17.2 | 22.3 | 20.0 |
| PI229343 | 5.9 | 21.8 | 20.1 | 22.4 | 20.5 | 17.3 | 13.9 | 26.5 | 24.7 | 17.5 | 19.5 | 25.5 | 17.7 | 21.2 | 19.6 |
| PI235335 | 5.9 | 21.1 | 20.1 | 21.3 | 22.2 | 16.1 | 10.0 | 33.4 | 27.2 | 17.5 | 23.2 | 23.4 | 17.2 | 22.4 | 20.1 |
| PI243521 | 8.5 | 21.5 | 20.1 | 20.1 | 13.4 | 14.1 | 10.7 | 24.6 | 22.6 | 21.6 | 20.8 | 17.4 | 15.4 | 19.8 | 17.5 |
| PI243528 | 7.8 | 21.5 | 20.1 | 28.0 | 25.3 | 14.8 | 12.6 | 30.9 | 33.2 | 22.6 | 28.2 | 26.9 | 19.6 | 25.9 | 23.5 |
| PI243545 | 9.1 | 21.5 | 20.1 | 20.1 | 14.6 | 14.7 | 9.7 | 25.4 | 22.2 | 16.5 | 18.3 | 20.1 | 15.9 | 18.4 | 17.0 |
| PI253651A | 8.0 | 21.1 | 20.1 | 20.1 | 12.2 | 12.4 | 16.9 | 29.0 | 22.2 | 22.6 | 26.9 | 15.5 | 14.6 | 22.5 | 18.8 |
| PI273483C | 7.2 | 22.9 | 20.1 | 23.5 | 19.0 | 17.8 | 10.7 | 29.0 | 28.6 | 24.7 | 25.7 | 27.4 | 18.5 | 24.6 | 22.1 |
| PI274421 | 7.8 | 22.4 | 20.1 | 21.3 | 17.4 | 19.0 | 11.3 | 34.0 | 28.9 | 19.6 | 18.3 | 25.5 | 17.7 | 22.9 | 20.7 |
| PI360846 | 8.5 | 21.8 | 20.1 | 23.5 | 21.7 | 17.3 | 15.2 | 34.0 | 29.3 | 25.7 | 22.0 | 25.5 | 18.6 | 25.6 | 22.8 |
| PI361103 | 9.1 | 22.8 | 20.1 | 20.1 | 20.5 | 14.8 | 15.2 | 33.4 | 26.5 | 22.6 | 20.8 | 18.2 | 17.7 | 23.0 | 20.7 |
| PI377574 | 12.9 | 22.1 | 20.1 | 19.0 | 16.9 | 13.6 | 13.3 | 40.2 | 29.3 | 20.6 | 28.2 | 22.8 | 16.9 | 26.0 | 22.0 |
| PI398198 | 5.9 | 21.3 | 20.1 | 15.9 | 16.9 | 13.1 | 11.0 | 26.4 | 26.8 | 16.0 | 22.0 | 12.8 | 14.3 | 18.9 | 16.4 |
| PI398202 | 7.8 | 21.5 | 20.1 | 20.1 | 17.4 | 12.4 | 15.9 | 35.9 | 32.5 | 17.5 | 20.8 | 20.1 | 15.8 | 23.7 | 20.1 |
| PI398223 | 5.9 | 21.1 | 20.1 | 15.6 | 16.9 | 11.2 | 8.0 | 28.4 | 25.4 | 15.4 | 19.5 | 14.2 | 14.0 | 18.4 | 15.9 |
| PI398228 | 6.6 | 22.8 | 20.1 | 19.0 | 21.0 | 15.3 | 14.6 | 32.1 | 39.8 | 18.5 | 25.7 | 24.2 | 17.1 | 25.7 | 22.0 |
| PI398245 | 5.9 | 22.1 | 20.1 | 21.3 | 12.2 | 11.2 | 16.5 | 33.4 | 30.7 | 17.5 | 23.2 | 14.7 | 14.6 | 22.7 | 18.8 |
| PI398249 | 8.5 | 21.5 | 20.1 | 19.0 | 13.4 | 12.4 | 19.5 | 27.8 | 26.5 | 22.6 | 24.5 | 17.4 | 14.8 | 23.3 | 19.3 |
| PI398272 | 9.1 | 23.1 | 20.1 | 14.5 | 17.5 | 12.4 | 15.2 | 33.4 | 25.4 | 15.4 | 23.2 | 17.4 | 15.4 | 21.6 | 18.6 |
| PI398298 | 5.9 | 21.8 | 20.1 | 19.0 | 14.6 | 11.2 | 12.6 | 35.9 | 25.8 | 19.6 | 28.2 | 16.1 | 14.5 | 23.2 | 19.1 |
| PI398321 | 7.2 | 21.1 | 20.1 | 20.1 | 21.0 | 14.8 | 10.7 | 28.4 | 24.7 | 20.6 | 25.7 | 16.6 | 16.8 | 21.3 | 19.2 |
| PI398396 | 5.9 | 21.5 | 20.1 | 15.6 | 14.6 | 12.4 | 12.0 | 25.9 | 27.9 | 14.4 | 20.8 | 13.4 | 13.8 | 18.8 | 16.1 |
| PI398492 | 7.8 | 21.8 | 20.1 | 16.8 | 13.4 | 12.4 | 8.0 | 30.3 | 24.4 | 17.5 | 17.1 | 17.4 | 14.3 | 19.0 | 16.5 |
| PI398601 | 5.9 | 24.3 | 20.1 | 28.9 | 21.7 | 21.8 | NA | 36.3 | 40.2 | 26.3 | 38.0 | 29.5 | 21.6 | 33.5 | 29.1 |
| PI398617 | 6.6 | 22.4 | 20.1 | 19.0 | 14.6 | 13.6 | 17.8 | 34.6 | 28.9 | 15.4 | 20.8 | 20.9 | 15.3 | 23.0 | 19.4 |
| PI398775 | 7.2 | 21.1 | 20.1 | 21.3 | 16.9 | 14.8 | 14.6 | 29.6 | 21.9 | 15.4 | 18.3 | 23.6 | 16.2 | 20.4 | 18.4 |
| PI398804 | 8.5 | 21.1 | 20.1 | 21.3 | 16.2 | 16.6 | 20.4 | 24.6 | 29.3 | 15.4 | 28.2 | 12.6 | 16.6 | 21.7 | 19.3 |
| PI398920 | 10.4 | 22.1 | 20.1 | 21.3 | 15.8 | 12.4 | 15.9 | NA | 21.5 | 18.5 | 22.0 | 17.4 | 16.4 | 20.5 | 18.6 |
| PI398965 | 7.2 | 21.5 | 20.1 | 23.5 | 15.8 | 17.3 | 15.2 | 27.1 | 21.2 | 15.4 | 20.8 | 20.1 | 17.0 | 19.9 | 18.5 |
| PI398970 | 9.1 | 21.6 | 20.1 | 21.3 | 16.9 | 12.4 | 12.9 | 22.8 | 24.4 | 18.5 | 18.3 | 18.8 | 16.2 | 19.2 | 17.6 |
| PI398982 | 6.6 | 21.3 | 20.1 | 19.0 | 20.5 | 12.4 | 12.6 | 31.5 | 40.2 | 17.5 | 25.7 | 20.1 | 15.8 | 24.5 | 20.6 |
| PI398987 | 10.4 | 23.4 | 20.1 | 21.3 | 15.8 | 14.8 | 18.8 | 26.5 | 24.4 | 20.6 | 19.5 | 18.8 | 17.4 | 21.5 | 19.7 |
| PI398997 | 8.5 | 21.1 | 20.1 | 19.0 | 21.0 | 18.5 | 10.0 | 29.0 | 30.0 | 20.6 | 23.2 | 22.3 | 17.5 | 22.6 | 20.3 |
| PI399027 | 6.6 | 21.8 | 20.1 | 22.4 | 13.4 | 13.6 | 17.8 | 24.1 | 30.3 | 19.6 | 17.1 | 20.1 | 15.5 | 21.5 | 18.6 |
| PI404164 | 9.8 | 22.4 | 20.1 | 22.4 | 21.0 | 12.4 | 23.0 | 24.6 | 25.8 | 20.6 | 25.7 | 25.5 | 17.8 | 24.3 | 21.5 |
| PI404190 | 10.4 | 21.1 | 20.1 | 24.6 | 20.6 | 13.6 | 8.0 | 23.4 | 21.2 | 20.6 | 22.0 | 22.0 | 18.0 | 19.6 | 18.8 |
| PI404191 | 10.4 | 21.5 | 20.1 | 23.5 | 13.8 | 18.5 | 18.2 | 34.6 | 30.7 | 21.6 | 26.9 | 22.0 | 17.5 | 25.9 | 22.3 |
| PI404199 | 8.5 | 21.3 | 20.1 | 16.8 | 16.0 | 11.2 | 9.3 | 24.0 | 28.9 | 11.3 | 15.9 | 16.1 | 14.4 | 17.1 | 15.3 |
| PI407734 | 8.5 | 22.1 | 20.1 | 25.8 | 13.8 | 13.6 | 14.6 | 25.9 | 21.5 | 19.6 | 24.5 | 18.5 | 16.9 | 20.9 | 19.0 |
| PI407735 | 12.3 | 21.3 | 20.1 | 14.5 | 15.8 | 13.6 | 11.3 | 25.3 | 21.5 | 16.5 | 29.4 | 13.9 | 15.2 | 19.7 | 17.4 |
| PI407821A | 6.6 | 21.1 | 20.1 | 19.0 | 14.6 | 13.6 | 11.3 | 31.5 | 25.8 | 19.6 | 22.0 | 18.0 | 14.8 | 21.4 | 18.2 |
| PI407892A | 8.5 | 21.5 | 20.1 | 21.3 | 18.1 | 14.8 | 15.9 | 24.0 | 31.4 | 16.5 | 20.8 | 22.0 | 16.8 | 21.5 | 19.3 |
| PI407927B | 6.6 | 22.3 | 20.1 | 10.0 | 12.2 | 11.2 | 9.3 | NA | 24.0 | 13.4 | 22.0 | 12.0 | 12.0 | 17.4 | 14.3 |
| PI408008 | 14.2 | 21.0 | 20.1 | 16.8 | 13.4 | 13.6 | 14.6 | 25.9 | 21.5 | 16.5 | 20.8 | 22.0 | 15.5 | 20.0 | 17.7 |
| PI408021 | 9.4 | 21.3 | 20.1 | 15.6 | 14.6 | 12.4 | 17.2 | 25.5 | 32.1 | 18.5 | 19.5 | 16.1 | 14.3 | 21.4 | 17.9 |
| PI408073 | 7.8 | 23.1 | 20.1 | 17.9 | 13.4 | 13.6 | 10.7 | 27.1 | 27.5 | 17.5 | 15.9 | 16.1 | 15.3 | 19.0 | 17.0 |
| PI408100B | 7.8 | 21.1 | 20.1 | 19.0 | 15.0 | 12.4 | 15.2 | 30.3 | 32.5 | 15.4 | 23.2 | 14.7 | 14.9 | 21.7 | 18.5 |
| PI408124A | 7.8 | 21.1 | 20.1 | 24.6 | 14.6 | 16.6 | 21.1 | 29.0 | 28.9 | 20.6 | 22.0 | 23.6 | 16.9 | 24.3 | 21.0 |
| PI408169D | 7.8 | 21.1 | 20.1 | 20.1 | 14.6 | 9.9 | 13.9 | 24.6 | 25.4 | 16.5 | 19.5 | 16.1 | 14.6 | 19.2 | 16.8 |
| PI408173 | 7.8 | 24.4 | 20.1 | 17.9 | 12.2 | 12.4 | 12.0 | 30.3 | 33.2 | 17.5 | 18.3 | 16.1 | 15.3 | 21.1 | 18.3 |
| PI408181D | 5.9 | 22.0 | 20.1 | 19.0 | 18.1 | 14.7 | NA | 28.0 | 25.1 | 17.5 | 23.2 | 20.1 | 16.0 | 21.5 | 18.9 |
| PI408189 | 5.9 | 22.4 | 20.1 | 17.9 | 13.4 | 12.4 | 16.2 | 27.8 | 22.6 | 13.4 | 18.3 | 16.1 | 14.5 | 18.8 | 16.5 |
| PI408209B | 10.2 | 21.6 | 20.1 | 21.7 | 19.8 | 11.4 | 22.5 | 25.6 | 27.2 | 19.8 | 29.4 | 19.1 | 17.0 | 24.3 | 21.1 |
| PI408212B | 7.1 | 21.3 | 20.1 | 21.7 | 18.1 | 13.1 | NA | 28.9 | 27.9 | 18.5 | 22.0 | 22.1 | 16.2 | 22.6 | 19.6 |
| PI408256 | 5.9 | 22.0 | 20.1 | 15.6 | 12.2 | 8.7 | 10.0 | 24.6 | 25.1 | 19.6 | 14.6 | 13.4 | 12.7 | 17.7 | 14.8 |
| PI408262C | 8.5 | 21.5 | 20.1 | 19.0 | 19.3 | 13.6 | 12.6 | 32.7 | 31.0 | 15.4 | 24.5 | 16.1 | 16.3 | 22.0 | 19.3 |
| PI408269B | 9.1 | 20.8 | 20.1 | 21.3 | 13.4 | 14.8 | 14.6 | 32.7 | 31.8 | 15.4 | 30.6 | 17.4 | 15.7 | 23.7 | 20.0 |
| PI408280 | 7.8 | 23.2 | 20.1 | 17.9 | 19.3 | 17.3 | 22.1 | 24.6 | 31.8 | 19.6 | 20.8 | 22.0 | 17.4 | 23.6 | 20.9 |
| PI416858 | 9.8 | 22.1 | 20.1 | 23.5 | 18.1 | 13.6 | 12.3 | 37.1 | 26.1 | 14.4 | 24.5 | 18.0 | 17.5 | 22.0 | 20.0 |
| PI416884 | 5.9 | 21.1 | 20.1 | 17.9 | 21.7 | 16.1 | 13.3 | 24.7 | 32.1 | 24.7 | 23.2 | 25.5 | 16.4 | 24.2 | 20.7 |
| PI416937 | 7.8 | 21.1 | 20.1 | 21.3 | 13.4 | 11.2 | 14.6 | 27.1 | 23.3 | 15.4 | 18.3 | 17.4 | 14.8 | 19.2 | 16.9 |
| PI416997 | 5.9 | 21.8 | 20.1 | 26.9 | 15.8 | 13.6 | 13.3 | 35.2 | 28.2 | 19.6 | 18.3 | 16.6 | 16.9 | 21.9 | 19.6 |
| PI417001 | 5.9 | 22.8 | 20.1 | 21.7 | 21.0 | 19.6 | 12.8 | 38.0 | 32.8 | 23.7 | 23.2 | 23.9 | 18.6 | 26.2 | 23.2 |
| PI417028 | 7.2 | 21.4 | 20.1 | 21.3 | 22.2 | 18.5 | 12.0 | 22.8 | 29.3 | 17.5 | 24.5 | 22.6 | 18.1 | 21.3 | 19.9 |
| PI417035 | 7.8 | 23.4 | 20.1 | 17.9 | 22.2 | 18.5 | 14.9 | 32.1 | 27.5 | 17.5 | 23.2 | 25.5 | 18.3 | 23.4 | 21.2 |
| PI417070 | 6.6 | 21.1 | 20.1 | 20.1 | 21.7 | 14.8 | 15.2 | 32.7 | 29.6 | 19.6 | 24.5 | 22.0 | 16.8 | 24.0 | 20.8 |
| PI417276 | 8.5 | 23.4 | 20.1 | 17.9 | 15.8 | 16.6 | 15.9 | 29.0 | 38.8 | 19.6 | 23.2 | 21.5 | 16.6 | 24.6 | 21.1 |
| PI417414B | 6.6 | 22.4 | 20.1 | 22.4 | 21.0 | 12.4 | 15.9 | 29.6 | 31.4 | 20.6 | 18.3 | 15.8 | 17.1 | 22.0 | 19.8 |
| PI417417 | 5.9 | 21.1 | 20.1 | 22.4 | 16.9 | 16.1 | 19.9 | 27.1 | 24.7 | 22.6 | 23.2 | 21.5 | 16.4 | 23.4 | 20.3 |
| PI417424 | 7.1 | 22.0 | 20.1 | 23.1 | 21.2 | 17.9 | NA | 28.9 | 33.5 | 21.1 | 23.2 | 23.6 | 18.7 | 24.9 | 22.5 |
| PI417432 | 7.8 | 22.4 | 20.1 | 23.5 | 19.3 | 16.6 | 13.9 | 40.8 | 39.8 | 18.5 | 29.4 | 21.5 | 18.1 | 27.4 | 23.5 |
| PI417495 | 7.8 | 21.1 | 20.1 | 16.8 | 22.2 | 17.3 | 18.2 | 41.5 | 36.0 | 17.5 | 30.6 | 22.8 | 16.9 | 27.8 | 23.2 |
| PI423741 | 5.9 | 22.1 | 20.1 | 19.0 | 15.8 | 16.8 | 11.3 | 23.4 | 26.5 | 19.6 | 22.0 | 17.4 | 16.0 | 20.0 | 18.0 |
| PI423747B | 9.8 | 21.4 | 20.1 | 19.0 | 23.4 | 14.8 | 15.5 | 34.6 | 27.9 | 17.9 | 31.9 | 20.1 | 17.6 | 24.8 | 21.7 |
| PI423748C | 8.5 | 22.1 | 20.1 | 19.0 | 13.8 | 9.8 | 20.8 | 24.6 | 24.7 | 21.6 | 19.5 | 17.4 | 14.6 | 21.6 | 18.2 |
| PI423799A | 7.8 | 21.3 | 20.1 | 17.9 | 13.4 | 12.4 | 12.0 | NA | 24.7 | 17.5 | 19.5 | 16.6 | 14.3 | 19.4 | 16.7 |
| PI423802 | 7.2 | 22.1 | 20.1 | 21.3 | 21.7 | 17.3 | 15.5 | 37.1 | 39.1 | 19.6 | 34.3 | 24.7 | 18.0 | 28.5 | 24.2 |
| PI423845A | 9.4 | 21.6 | 20.1 | 21.3 | 19.3 | 13.1 | 19.0 | NA | 40.2 | 16.5 | 28.2 | 24.7 | 16.9 | 26.7 | 22.4 |
| PI423890B | 11.0 | 21.8 | 20.1 | 30.3 | 21.0 | 19.3 | 16.5 | 29.0 | 25.1 | 26.8 | 20.8 | 24.2 | 20.9 | 24.1 | 23.0 |
| PI423890C | 11.7 | 22.1 | 20.1 | 29.1 | 21.5 | 21.0 | 19.1 | 31.5 | 33.5 | 27.8 | 31.9 | 30.1 | 21.3 | 29.4 | 26.5 |
| PI424154A | 7.2 | 21.8 | 20.1 | 20.1 | 16.9 | 12.4 | 14.6 | 32.7 | 26.5 | 20.6 | 25.7 | 23.4 | 15.7 | 24.1 | 20.3 |
| PI424231 | 7.8 | 21.1 | 20.1 | 17.9 | 18.1 | 12.4 | 12.6 | 25.9 | 24.0 | 26.8 | 20.8 | 16.1 | 15.3 | 21.4 | 18.5 |
| PI424247B | 12.9 | 21.4 | 20.1 | 23.5 | 23.7 | 17.3 | 22.7 | 29.0 | 40.6 | 19.6 | 24.5 | 18.0 | 20.0 | 25.7 | 23.5 |
| PI424355 | 5.9 | 21.3 | 20.1 | 15.6 | 19.3 | 12.4 | 13.3 | 32.7 | 29.3 | 19.6 | 18.3 | 18.0 | 14.7 | 21.9 | 18.4 |
| PI424397 | 7.8 | 24.4 | 20.1 | 21.3 | 19.3 | 14.4 | 14.6 | 34.0 | 37.0 | 17.5 | 23.2 | 24.2 | 17.9 | 25.0 | 22.0 |
| PI424399 | 5.9 | 21.1 | 20.1 | 15.9 | 15.0 | 11.4 | NA | 24.0 | 24.0 | 18.5 | 23.2 | 14.7 | 13.6 | 19.8 | 16.5 |
| PI424401 | 9.8 | 23.1 | 20.1 | 21.3 | 12.2 | 11.2 | 35.4 | 30.9 | 35.3 | 15.4 | 25.7 | 24.7 | 15.7 | 27.7 | 22.5 |
| PI424435 | 9.1 | 22.1 | 20.1 | 19.0 | 14.6 | 21.5 | 17.2 | 36.5 | 39.8 | 20.6 | 22.0 | 26.9 | 17.3 | 27.2 | 23.0 |
| PI424488B | 5.9 | 21.1 | 20.1 | 16.8 | 16.9 | 11.2 | 15.5 | 29.6 | 36.3 | 13.4 | 19.5 | 17.4 | 14.2 | 21.6 | 18.0 |
| PI424513 | 8.5 | 20.8 | 20.1 | 17.9 | 16.9 | 16.6 | 12.0 | 25.9 | 30.3 | 17.5 | 22.0 | 20.1 | 15.9 | 21.2 | 18.7 |
| PI424546A | 5.9 | 21.5 | 20.1 | 15.6 | 12.2 | 12.4 | 13.9 | 30.9 | 25.8 | 12.4 | 24.5 | 18.0 | 13.3 | 20.7 | 17.0 |
| PI424614 | 8.5 | 21.1 | 20.1 | 21.3 | 18.1 | 15.3 | 15.9 | 27.1 | 36.7 | 15.4 | 24.5 | 17.4 | 16.8 | 22.6 | 20.0 |
| PI430598A | 9.8 | 23.1 | 20.1 | 26.9 | 15.8 | 13.6 | 17.2 | 25.3 | 22.9 | 18.5 | 22.0 | 21.5 | 18.2 | 21.2 | 19.9 |
| PI432359 | 7.2 | 22.4 | 20.1 | 22.4 | 19.3 | 11.2 | 11.3 | 32.7 | 28.6 | 19.6 | 23.2 | 20.1 | 16.7 | 22.7 | 19.9 |
| PI437745 | 7.8 | 24.1 | 20.1 | 22.4 | 14.6 | 14.8 | 15.2 | 35.9 | 25.4 | 15.4 | 26.9 | 22.8 | 17.2 | 23.6 | 20.8 |
| PI438424 | 6.6 | 21.1 | 20.1 | 16.8 | 21.0 | 12.4 | 17.8 | 32.9 | 27.9 | 17.5 | 22.0 | 18.8 | 15.4 | 22.8 | 19.3 |
| PI442006 | 5.9 | 21.1 | 20.1 | 13.4 | 14.6 | 9.9 | 9.3 | 27.1 | 23.7 | 14.4 | 23.2 | 12.0 | 12.7 | 18.2 | 15.2 |
| PI442012A | 9.8 | 21.5 | 20.1 | 17.9 | 16.9 | 12.4 | 13.9 | 32.7 | 29.3 | 21.6 | 23.2 | 22.6 | 15.5 | 24.0 | 20.2 |
| PI458084 | 13.6 | 21.8 | 20.1 | 17.9 | 18.1 | 16.6 | 26.6 | 30.3 | 33.2 | 16.5 | 23.2 | 21.5 | 17.5 | 25.1 | 21.8 |
| PI458098 | 9.1 | 21.5 | 20.1 | 19.0 | 12.2 | 12.9 | 15.2 | 27.8 | 22.6 | 14.4 | 20.8 | 12.0 | 14.8 | 18.7 | 16.6 |
| PI458119 | 7.2 | 21.1 | 20.1 | 16.8 | 14.6 | 16.1 | 12.0 | 24.4 | 22.6 | 21.6 | 13.4 | 17.4 | 14.9 | 18.6 | 16.6 |
| PI458515 | 13.6 | 22.4 | 20.1 | 24.6 | 16.9 | 14.8 | 19.8 | 27.8 | 26.8 | 21.6 | 22.0 | 18.8 | 18.6 | 22.9 | 21.2 |
| PI468923 | 6.1 | 21.3 | 20.1 | 17.9 | 16.9 | 11.2 | 17.2 | 25.3 | 23.3 | 16.5 | 18.3 | 15.5 | 14.5 | 19.2 | 16.7 |
| PI471938 | 8.5 | 21.1 | 20.1 | 16.8 | 13.4 | 16.1 | 13.9 | 24.6 | 21.5 | 16.5 | 14.6 | 25.5 | 14.9 | 19.3 | 17.0 |
| PI476939 | 5.9 | 21.1 | 20.1 | 19.0 | 16.9 | 14.8 | 8.7 | 29.0 | 22.6 | 17.5 | 24.5 | 20.1 | 15.4 | 20.4 | 17.9 |
| PI495017B | 16.1 | 21.5 | 20.1 | 20.1 | 16.9 | 13.6 | 19.1 | 24.6 | 21.2 | 20.6 | 19.5 | 18.5 | 17.5 | 20.7 | 19.2 |
| PI506519 | 5.9 | 22.8 | 20.1 | 19.0 | 20.6 | 13.6 | 9.3 | 22.2 | 25.4 | 14.4 | 15.9 | 19.3 | 16.5 | 17.4 | 16.7 |
| PI506937 | 6.6 | 22.0 | 20.1 | 17.9 | 19.8 | 14.8 | 10.7 | 27.8 | 25.1 | 19.6 | 19.5 | 23.4 | 16.2 | 21.0 | 18.7 |
| PI507025 | 8.5 | 21.8 | 20.1 | 14.5 | 19.1 | 12.4 | 8.7 | 30.3 | 30.7 | 14.4 | 22.0 | 21.5 | 15.0 | 21.0 | 18.1 |
| PI507067 | 9.8 | 21.1 | 20.1 | 20.1 | 13.4 | 16.1 | 13.9 | 30.3 | 30.7 | 18.5 | 18.3 | 22.3 | 15.9 | 22.2 | 19.3 |
| PI507073 | 17.0 | 21.3 | 20.1 | 21.3 | 19.3 | 17.3 | 13.7 | 25.5 | 23.7 | 17.5 | 22.0 | 26.1 | 19.1 | 21.3 | 20.5 |
| PI507311 | 14.2 | 21.8 | 20.1 | 22.4 | 19.3 | 16.1 | 19.5 | 24.7 | 28.2 | 20.6 | 26.9 | 21.5 | 18.8 | 23.7 | 21.7 |
| PI507395 | 7.2 | 21.1 | 20.1 | 28.0 | 19.9 | 17.8 | 13.9 | 25.9 | 34.2 | 22.6 | 20.8 | 25.5 | 18.9 | 23.9 | 21.8 |
| PI507407 | 32.7 | 23.8 | 20.1 | 35.9 | 27.0 | 27.1 | 50.8 | 32.1 | 36.7 | 32.9 | 41.7 | 33.1 | 29.8 | 38.7 | 36.6 |
| PI507408 | 22.5 | 22.4 | 20.1 | 34.8 | 31.8 | 25.9 | 46.5 | 41.5 | 39.1 | 32.9 | 44.2 | 33.6 | 27.9 | 40.6 | 36.6 |
| PI507424 | 24.4 | 24.1 | 20.1 | 23.5 | 24.6 | 21.5 | 37.7 | 33.4 | 36.0 | 29.8 | 35.6 | 29.6 | 24.0 | 34.3 | 30.8 |
| PI507435 | 5.9 | 21.8 | 20.1 | 22.4 | 19.0 | 14.8 | 12.0 | NA | 22.6 | 19.6 | 15.9 | 24.2 | 16.8 | 20.2 | 18.6 |
| PI507446 | 7.8 | 21.1 | 20.1 | 23.5 | 19.3 | 16.1 | 10.0 | 30.3 | 32.5 | 17.5 | 20.8 | 18.8 | 17.5 | 21.5 | 19.7 |
| PI507449 | 11.9 | 23.2 | 20.1 | 23.1 | NA | 14.7 | 11.9 | 31.4 | 28.9 | 14.7 | 14.6 | 18.4 | 18.9 | 19.5 | 19.0 |
| PI509079 | 7.2 | 20.8 | 20.1 | 21.3 | 22.9 | 11.2 | 16.5 | 32.7 | 37.0 | 15.4 | 26.9 | 20.1 | 16.5 | 24.6 | 21.1 |
| PI518673 | 8.5 | 21.1 | 20.1 | 17.9 | 17.9 | 12.4 | 12.0 | 30.9 | 22.2 | 17.5 | 24.3 | 22.8 | 15.4 | 21.6 | 18.6 |
| PI532462B | 7.8 | 22.4 | 20.1 | 22.4 | 17.4 | 16.1 | 15.9 | 34.0 | 28.2 | 19.6 | 22.0 | 22.8 | 17.4 | 23.8 | 21.0 |
| PI532466A | 5.9 | 22.4 | 20.1 | 14.5 | 19.3 | 12.4 | 12.0 | 29.6 | 33.9 | 16.5 | 28.2 | 18.2 | 14.9 | 23.0 | 19.2 |
| PI538380 | 7.8 | 21.1 | 20.1 | 16.8 | 20.5 | 11.2 | 16.2 | 26.5 | 25.8 | 12.4 | 25.7 | 18.8 | 15.3 | 20.6 | 18.0 |
| PI548169 | 6.6 | 22.4 | 20.1 | 16.8 | NA | 11.2 | 13.9 | 32.7 | 25.1 | 12.4 | 18.3 | 14.2 | 14.7 | 19.2 | 16.7 |
| PI548250 | 6.6 | 21.1 | 20.1 | 16.8 | 9.8 | 11.2 | 11.3 | 24.6 | 24.0 | 16.5 | 22.0 | 19.3 | 12.8 | 19.5 | 16.0 |
| PI548314 | 10.4 | 22.8 | 20.1 | 20.1 | 20.5 | 18.0 | 21.7 | 24.0 | 21.5 | 21.6 | 28.2 | 26.1 | 18.5 | 24.1 | 21.8 |
| PI548359 | 7.2 | 21.1 | 20.1 | 19.0 | 14.6 | 13.6 | 10.7 | 25.2 | 21.2 | 17.5 | 25.7 | 23.4 | 14.9 | 20.6 | 17.8 |
| PI548427 | 7.8 | 22.4 | 20.1 | 20.1 | 13.4 | 12.4 | 11.3 | 23.4 | 21.5 | 18.5 | 18.3 | 23.4 | 15.3 | 19.4 | 17.2 |
| PI548430 | 8.5 | 22.1 | 20.1 | 24.6 | 15.9 | 14.8 | 11.3 | 24.6 | 23.7 | 19.6 | 20.8 | 19.3 | 17.4 | 19.9 | 18.6 |
| PI548431 | 11.7 | 21.8 | 20.1 | 17.9 | 20.5 | 14.8 | 12.0 | 22.8 | 22.9 | 20.6 | 28.2 | 22.0 | 17.3 | 21.5 | 19.6 |
| PI561303 | 11.0 | 22.0 | 20.1 | 22.4 | 16.9 | 16.1 | 21.1 | 32.7 | 31.4 | 19.6 | 33.1 | 19.6 | 17.8 | 26.4 | 22.8 |
| PI567201D | 8.5 | 23.8 | 20.1 | 13.4 | 15.8 | 13.6 | 20.4 | 34.0 | 28.9 | 14.4 | 14.6 | 25.5 | 15.2 | 22.7 | 19.2 |
| PI567202 | 8.5 | 21.1 | 20.1 | 19.0 | 16.9 | 16.1 | 10.3 | 34.6 | 24.7 | 18.5 | 23.2 | 21.5 | 16.2 | 22.2 | 19.4 |
| PI567272B | 10.4 | 22.1 | 20.1 | 22.4 | 25.1 | 14.4 | 31.2 | 32.1 | 36.7 | 20.6 | 34.3 | 25.5 | 19.0 | 30.2 | 25.7 |
| PI567291 | 9.8 | 25.1 | 20.1 | 23.5 | 16.0 | 14.1 | 17.8 | 27.8 | 21.2 | 23.1 | 18.3 | 28.2 | 18.5 | 22.9 | 21.0 |
| PI567447A | 5.9 | 22.1 | 20.1 | 17.9 | 13.4 | 13.6 | 7.4 | 25.3 | 21.2 | 15.4 | 22.0 | 17.4 | 14.6 | 18.0 | 16.0 |
| PI567482B | 14.9 | 22.8 | 20.1 | 19.0 | 15.8 | 15.6 | 21.1 | 29.0 | 23.3 | 21.6 | 30.6 | 22.0 | 17.7 | 24.9 | 21.8 |
| PI567488A | 18.7 | 22.1 | 20.1 | 23.5 | 21.0 | 13.6 | 26.3 | 24.7 | 31.8 | 13.4 | 26.9 | 26.1 | 19.9 | 24.7 | 22.9 |
| PI567488B | 16.1 | 23.4 | 20.1 | 17.9 | 16.9 | 12.4 | 25.0 | 27.8 | 24.0 | 19.6 | 23.2 | 29.0 | 17.5 | 24.8 | 21.7 |
| PI567488C | 12.9 | 21.8 | 20.1 | 20.1 | 18.6 | 14.8 | 17.8 | 28.4 | 29.3 | 19.6 | 22.0 | 29.0 | 17.6 | 24.3 | 21.5 |
| PI567489B | 11.0 | 22.1 | 20.1 | 20.1 | 18.1 | 14.8 | 16.5 | 24.6 | 30.7 | 18.5 | 26.9 | 26.9 | 17.3 | 24.0 | 21.1 |
| PI567491B | 7.2 | 22.1 | 20.1 | 17.2 | 14.6 | 12.4 | 9.3 | 23.4 | 29.6 | 18.5 | 19.5 | 16.1 | 14.7 | 19.3 | 16.9 |
| PI567527 | 8.5 | 21.8 | 20.1 | 21.3 | 16.9 | 13.6 | 12.6 | 31.5 | 24.7 | 16.5 | 22.0 | 27.7 | 16.4 | 22.4 | 19.7 |
| PI567531 | 9.1 | 22.4 | 20.1 | 25.8 | 21.0 | 13.6 | 21.4 | 29.0 | 31.0 | 20.6 | 28.2 | 21.5 | 18.6 | 25.4 | 22.6 |
| PI567532 | 7.8 | 21.8 | 20.1 | 20.1 | 14.6 | 11.2 | 12.9 | 23.7 | 25.4 | 21.6 | 20.8 | 16.1 | 15.1 | 20.2 | 17.6 |
| PI567548 | 6.6 | 22.1 | 20.1 | 26.9 | 14.6 | 13.6 | 13.3 | 24.0 | 21.2 | 19.6 | 17.1 | 27.4 | 16.9 | 20.4 | 18.7 |
| PI567572B | 7.8 | 21.1 | 20.1 | 22.4 | 15.0 | 14.8 | 8.0 | 30.9 | 26.5 | 17.5 | 23.2 | 17.4 | 16.2 | 20.6 | 18.4 |
| PI567593B | 7.8 | 21.1 | 20.1 | 17.9 | 21.7 | 12.4 | 18.2 | 24.0 | 21.9 | 18.5 | 18.3 | 16.1 | 16.1 | 19.5 | 17.7 |
| PI567616 | 8.5 | 22.1 | 20.1 | 22.4 | 13.7 | 13.6 | 10.7 | 22.8 | 23.7 | 20.0 | 17.1 | 21.5 | 16.3 | 19.2 | 17.6 |
| PI567659 | 11.7 | 21.1 | 20.1 | 24.0 | NA | 14.8 | 16.2 | 24.6 | 22.2 | 14.4 | 18.3 | 20.1 | 18.0 | 19.1 | 18.3 |
| PI567753C | 7.2 | 21.1 | 20.1 | 20.1 | 23.7 | 14.1 | 10.0 | 22.8 | 21.5 | 16.5 | 19.5 | 20.7 | 17.1 | 18.4 | 17.6 |
| PI567762A | 20.6 | 21.1 | 20.1 | 25.8 | 19.8 | 17.3 | 31.5 | 30.3 | 28.6 | 23.7 | 29.4 | 29.6 | 20.8 | 29.1 | 26.0 |
| PI567777 | 13.6 | 21.4 | 20.1 | 16.8 | 16.9 | 13.6 | 23.0 | 25.3 | 21.2 | 20.6 | 28.2 | 22.0 | 16.3 | 23.6 | 20.3 |
| PI574477 | 12.3 | 21.8 | 20.1 | 24.6 | 15.8 | 14.8 | 21.7 | 37.1 | 33.2 | 21.6 | 22.0 | 25.5 | 17.9 | 27.0 | 23.2 |
| PI578494A | 7.8 | 21.8 | 20.1 | 17.9 | 16.2 | 13.6 | 13.9 | 26.5 | 27.9 | 19.6 | 18.3 | 21.2 | 15.4 | 21.2 | 18.4 |
| PI587588A | 9.1 | 21.1 | 20.1 | 25.8 | 18.1 | 12.4 | 14.6 | 27.1 | 24.4 | 21.6 | 22.0 | 21.5 | 17.3 | 22.0 | 19.9 |
| PI58955 | 19.3 | 22.1 | 20.1 | 22.4 | 18.6 | 16.1 | 31.2 | 32.7 | 25.4 | 19.6 | 34.3 | 26.9 | 19.7 | 28.6 | 25.1 |
| PI594160 | 16.2 | 22.4 | 20.1 | 24.6 | 24.1 | 16.1 | 34.5 | 32.1 | 24.7 | 18.5 | 24.5 | 24.7 | 21.0 | 26.7 | 24.7 |
| PI594289 | 6.6 | 21.1 | 20.1 | 22.4 | 20.6 | 14.1 | 19.5 | 30.3 | 30.3 | 16.5 | 23.2 | 18.0 | 16.9 | 22.9 | 20.2 |
| PI594399C | 6.6 | 21.8 | 20.1 | 19.0 | 18.1 | 17.3 | 17.2 | 27.8 | 30.3 | 19.6 | 19.5 | 20.1 | 16.5 | 22.4 | 19.7 |
| PI594410 | 10.4 | 22.1 | 20.1 | 22.4 | 18.6 | 16.1 | 19.1 | 30.9 | 26.8 | 18.5 | 24.5 | 18.8 | 18.0 | 23.2 | 21.0 |
| PI594664 | 8.5 | 21.5 | 20.1 | 15.6 | 14.6 | 13.6 | 13.3 | 24.6 | 21.2 | 19.6 | 15.9 | 12.0 | 14.6 | 17.8 | 15.9 |
| PI597480A | 7.2 | 21.1 | 20.1 | 19.0 | 20.5 | 11.2 | 12.0 | 24.6 | 22.9 | 16.5 | 17.1 | 18.2 | 15.7 | 18.4 | 16.9 |
| PI597485 | 10.4 | 22.4 | 20.1 | 19.0 | 19.3 | 15.6 | 20.4 | 25.3 | 30.7 | 20.6 | 24.5 | 23.6 | 17.4 | 24.2 | 21.3 |
| PI603166 | 7.8 | 22.1 | 20.1 | 19.0 | 15.0 | 13.6 | 15.9 | 27.1 | 24.7 | 18.5 | 22.0 | 20.9 | 15.5 | 21.5 | 18.7 |
| PI603171 | 7.8 | 22.1 | 20.1 | 21.3 | 13.4 | 16.1 | 17.2 | 26.5 | 27.2 | 19.6 | 23.2 | 24.2 | 16.2 | 23.0 | 19.9 |
| PI603418D | 9.1 | 21.8 | 20.1 | 23.5 | 19.3 | 15.3 | 20.4 | 25.9 | 22.9 | 12.4 | 18.3 | 23.6 | 17.9 | 20.3 | 19.2 |
| PI603454 | 7.2 | 21.8 | 20.1 | 16.8 | 15.0 | 11.2 | 16.5 | 29.0 | 27.9 | 19.6 | 23.2 | 20.1 | 14.3 | 22.8 | 18.8 |
| PI603457A | 5.9 | 21.8 | 20.1 | 15.6 | 15.8 | 12.9 | 10.7 | 24.6 | 22.9 | 17.5 | 15.9 | 12.0 | 14.3 | 17.2 | 15.4 |
| PI603458A | 9.1 | 21.1 | 20.1 | 15.6 | 14.6 | 13.6 | 10.0 | 26.5 | 21.5 | 19.6 | 17.1 | 22.8 | 14.6 | 19.6 | 17.0 |
| PI603465C | 9.8 | 21.1 | 20.1 | 20.1 | 16.9 | 16.8 | 17.8 | 27.2 | 27.2 | 19.6 | 18.3 | 18.2 | 16.8 | 21.4 | 19.3 |
| PI603489 | 6.6 | 22.1 | 20.1 | 24.0 | 20.5 | 11.2 | 13.3 | 26.5 | 29.6 | 15.4 | 20.8 | 21.5 | 17.0 | 21.0 | 19.1 |
| PI603490 | 8.5 | 21.5 | 20.1 | 19.0 | 13.8 | 12.4 | 12.6 | 26.5 | 28.6 | 20.6 | 26.9 | 24.2 | 14.9 | 23.3 | 19.4 |
| PI603543B | 5.9 | 21.1 | 20.1 | 17.9 | 13.4 | 11.2 | 8.7 | 27.0 | 24.7 | 19.6 | 18.3 | 16.6 | 13.7 | 19.1 | 16.2 |
| PI603554B | 14.2 | 23.4 | 20.1 | 20.1 | 15.8 | 12.4 | 21.7 | 26.5 | 25.1 | 18.5 | 22.0 | 26.3 | 17.4 | 23.3 | 20.7 |
| PI603555 | 11.0 | 21.8 | 20.1 | 23.5 | 13.4 | 17.3 | 8.7 | 24.6 | 24.7 | 19.6 | 17.1 | 16.1 | 17.4 | 18.4 | 17.8 |
| PI603563B | 6.6 | 22.8 | 20.1 | 16.8 | 15.8 | 12.4 | 12.6 | 25.3 | 24.7 | 21.6 | 17.1 | 20.1 | 14.9 | 20.3 | 17.6 |
| PI603692 | 5.9 | 21.1 | 20.1 | 15.6 | 14.6 | 12.4 | 10.0 | 24.6 | 21.2 | 14.4 | 17.1 | 16.1 | 13.7 | 17.0 | 15.0 |
| PI603909B | 5.9 | 21.5 | 20.1 | 24.6 | 17.4 | 15.3 | 15.5 | 29.0 | 21.5 | 24.7 | 23.2 | 23.4 | 17.0 | 23.2 | 20.5 |
| PI603911C | 7.8 | 20.8 | 20.1 | 16.8 | 19.3 | 14.8 | 18.2 | 24.0 | 25.1 | 18.5 | 18.3 | 16.6 | 15.7 | 20.1 | 17.9 |
| PI603917 | 6.6 | 21.1 | 20.1 | 20.1 | 14.6 | 14.1 | 10.0 | 23.4 | 21.5 | 17.5 | 18.3 | 15.3 | 15.2 | 17.6 | 16.1 |
| PI606435 | 5.9 | 21.1 | 20.1 | 17.9 | 16.9 | 11.2 | 12.6 | 27.8 | 26.5 | 18.5 | 25.7 | 20.7 | 14.5 | 22.0 | 18.4 |
| PI612612A | 10.4 | 23.4 | 20.1 | 20.1 | 17.4 | 16.1 | 22.4 | 27.1 | 24.0 | 17.5 | 24.5 | 25.0 | 17.8 | 23.4 | 21.0 |
| PI632428 | 6.6 | 21.5 | 20.1 | 15.6 | 24.1 | 12.4 | 10.0 | 23.4 | 24.7 | 14.4 | 19.5 | 12.3 | 15.9 | 17.2 | 16.3 |
| PI642055 | 14.9 | 21.1 | 20.1 | 29.1 | 22.9 | 16.1 | 20.1 | 28.4 | 27.2 | 23.7 | 29.4 | 26.9 | 20.9 | 26.2 | 24.3 |
| PI70229 | 19.4 | 21.3 | 20.1 | 21.3 | 18.0 | 16.1 | 24.7 | 27.9 | 34.6 | 19.6 | 28.2 | 24.7 | 19.2 | 26.9 | 23.8 |
| PI80479 | 5.9 | 21.1 | 20.1 | 20.1 | 21.3 | 16.1 | 17.2 | 32.7 | 33.5 | 18.5 | 25.7 | 20.1 | 16.8 | 24.6 | 21.2 |
| PI81030 | 7.8 | 21.1 | 20.1 | 21.3 | 16.6 | 12.4 | 9.3 | 27.8 | 30.3 | 14.4 | 22.0 | 16.1 | 15.7 | 19.8 | 17.6 |
| PI84594 | 7.8 | 23.4 | 20.1 | 23.5 | 14.4 | 14.8 | 19.8 | 26.5 | 22.6 | 19.6 | 23.2 | 25.5 | 17.3 | 22.9 | 20.4 |
| PI84628 | 5.9 | 21.1 | 20.1 | 14.5 | 19.3 | 12.4 | 9.3 | 27.1 | 21.2 | 16.5 | 19.5 | 10.7 | 14.5 | 17.3 | 15.6 |
| PI89074 | 17.4 | 23.1 | 20.1 | 21.3 | 25.9 | 13.6 | 18.5 | 28.4 | 38.4 | 25.7 | 26.9 | 27.4 | 20.5 | 27.8 | 25.1 |
| PI90305 | 6.6 | 22.1 | 20.1 | 17.9 | 19.0 | 9.9 | 10.7 | NA | 29.3 | 15.4 | 25.7 | 13.4 | 15.1 | 20.1 | 17.6 |
| PI92641B | 7.8 | 21.1 | 20.1 | 20.1 | 14.6 | 14.8 | 13.6 | 29.0 | 33.5 | 17.5 | 25.7 | 15.5 | 15.6 | 22.4 | 19.2 |
| PI96280 | 5.9 | 21.1 | 20.1 | 17.9 | 16.9 | 12.9 | 12.6 | 25.9 | 22.6 | 14.4 | 25.7 | 16.1 | 14.8 | 19.4 | 17.0 |
| PI96333 | 7.8 | 21.5 | 20.1 | 23.5 | 19.3 | 16.6 | 24.4 | 30.9 | 30.0 | 22.6 | 26.9 | 22.0 | 17.8 | 26.4 | 22.7 |

| Rank | Ave_IR | Canopy Wilting score^a^ | Ave_DR | Canopy Wilting score |
| --- | --- | --- | --- | --- |
| **Slower wilting genotypes** | | | | |
| 1 | PI407927B | 13 | PI407927B | 15 |
| 2 | PI442006 | 13 | PI603692 | 16 |
| 3 | PI548250 | 13 | PI404199 | 16 |
| 4 | PI408256 | 13 | PI632428 | 16 |
| 5 | PI424546A | 13 | PI603457A | 16 |
| 6 | PI603543B | 14 | PI084628 | 16 |
| 7 | PI603692 | 14 | PI408256 | 16 |
| 8 | PI424399 | 14 | PI506519 | 16 |
| 9 | PI398396 | 14 | PI603917 | 16 |
| 10 | PI398223 | 14 | PI594664 | 17 |
| 11 | PI424488B | 14 | PI567447A | 17 |
| 12 |  |  | PI442006 | 17 |
| 13 |  |  | PI423799A | 17 |
| 14 |  |  | PI243545 | 17 |
| 15 |  |  | PI398223 | 17 |
| 16 |  |  | PI567753C | 17 |
| 17 |  |  | PI597480A | 17 |
| 18 |  |  | PI603555 | 17 |
| **Faster wilting genotypes** | | | | |
| 1 | PI507424 | 26 | PI507424 | 37 |
| 2 | PI507408 | 30 | PI398601 | 39 |
| 3 | PI507407 | 32 | PI507407 | 43 |
| 4 |  |  | PI507408 | 45 |

**Table S3.** Accessions identified as wilting slower or faster than slow-wilting (PI416937 and PI471938) and fast-wilting (A5959 and 08705_16) checks when averaged over irrigated (Ave_IR) and drought (Ave_DR) treatments.

^a^Standard deviation values from analysis of variance for Ave_IR and Ave_DR were 6.8 and 8.9, respectively.

|  | CO18IR | MC18IR | MC19IR | PT18IR | PT19IR | RH18IR | CO18DR | MC18DR | MC19DR | PT18DR | PT19DR | RH18DR | Ave_IR | Ave_DR | AAE |
| --- | --- | --- | --- | --- | --- | --- | --- | --- | --- | --- | --- | --- | --- | --- | --- |
| CO18IR | 1 |  |  |  |  |  |  |  |  |  |  |  |  |  |  |
| MC18IR | 0.24*** | 1 |  |  |  |  |  |  |  |  |  |  |  |  |  |
| MC19IR | 0.14* | 0.17* | 1 |  |  |  |  |  |  |  |  |  |  |  |  |
| PT18IR | 0.43*** | 0.22** | 0.16* | 1 |  |  |  |  |  |  |  |  |  |  |  |
| PT19IR | 0.34*** | 0.08 | 0.08 | 0.39*** | 1 |  |  |  |  |  |  |  |  |  |  |
| RH18IR | 0.45*** | 0.29*** | 0.09 | 0.58*** | 0.46*** | 1 |  |  |  |  |  |  |  |  |  |
| CO18DR | 0.73*** | 0.31*** | 0.13 | 0.48*** | 0.39*** | 0.46*** | 1 |  |  |  |  |  |  |  |  |
| MC18DR | 0.12 | 0.27*** | 0.08 | 0.24*** | 0.26*** | 0.32*** | 0.24*** | 1 |  |  |  |  |  |  |  |
| MC19DR | 0.15* | 0.17* | 0.11 | 0.23*** | 0.39*** | 0.34*** | 0.30*** | 0.50*** | 1 |  |  |  |  |  |  |
| PT18DR | 0.42*** | 0.26*** | 0.15* | 0.55*** | 0.37*** | 0.57*** | 0.44*** | 0.18* | 0.20** | 1 |  |  |  |  |  |
| PT19DR | 0.49*** | 0.16* | 0.12 | 0.40*** | 0.46*** | 0.45*** | 0.57*** | 0.44*** | 0.45*** | 0.42*** | 1 |  |  |  |  |
| RH18DR | 0.50*** | 0.39*** | 0.03 | 0.57*** | 0.43*** | 0.62*** | 0.53*** | 0.28*** | 0.28*** | 0.53*** | 0.45*** | 1 |  |  |  |
| Ave_IR | 0.73*** | 0.41*** | 0.19** | 0.79*** | 0.70*** | 0.79*** | 0.70*** | 0.33*** | 0.37*** | 0.65*** | 0.60*** | 0.72*** | 1 |  |  |
| Ave_DR | 0.63*** | 0.35*** | 0.11 | 0.56*** | 0.54*** | 0.64*** | 0.78*** | 0.60*** | 0.62*** | 0.64*** | 0.80*** | 0.71*** | 0.80*** | 1 |  |
| AAE | 0.63*** | 0.40*** | 0.17* | 0.70*** | 0.63*** | 0.74*** | 0.79*** | 0.53*** | 0.57*** | 0.68*** | 0.76*** | 0.76*** | 0.92*** | 0.97*** | 1 |
| Significant at *P*= < 0.001***, < 0.01**, < 0.05* | | | | | | | |  |  |  |  |  |  |  |  |

**Table S4.** Correlations for canopy wilting (n=206) for Columbia (CO18), Maricopa (MC18&19), Pine Tree (PT18&19), Rohwer (RH18) irrigated (IR) and drought (DR) treatments, averaged over irrigated (Ave_IR) and averaged drought (Ave_DR) treatments, and averaged across all environments (AAE).

| **Top 10 slowest wilting accessions** | | | | | | | | |
| --- | --- | --- | --- | --- | --- | --- | --- | --- |
| Rank | MG 000 | Predicted CW score | MG 00 | Predicted CW score | MG 0 | Predicted CW score | MG I | Predicted CW score |
| 1 | PI507830B | 13 | PI507679A | 11 | PI612761A | 9 | PI79648 | 9 |
| 2 | PI507834 | 14 | PI567226 | 12 | PI612756 | 9 | PI79694 | 9 |
| 3 | PI507840 | 14 | PI639567 | 13 | PI612757 | 9 | PI401418 | 10 |
| 4 | PI507841A | 14 | PI597394 | 13 | PI612755 | 11 | PI326580 | 10 |
| 5 | PI507741B | 14 | PI603426A | 13 | PI612753A | 11 | PI506678 | 10 |
| 6 | PI507766 | 14 | PI507842 | 13 | PI437288 | 11 | PI291309C | 10 |
| 7 | PI578356 | 14 | PI437298 | 13 | PI437286 | 12 | PI291275 | 10 |
| 8 | PI507741A | 14 | PI437532 | 14 | PI189951 | 12 | PI291277 | 10 |
| 9 | PI507767 | 14 | PI437219 | 14 | PI437216 | 12 | PI437658 | 10 |
| 10 | PI507755 | 14 | PI437220 | 14 | PI437251A | 12 | PI326579 | 11 |
| Rank | MG II | Predicted CW score | MG III | Predicted CW score | MG IV | Predicted CW score | MG V | Predicted CW score |
| 1 | PI437791 | 10 | PI81766 | 11 | PI507180 | 11 | PI561373 | 9 |
| 2 | PI438153 | 10 | PI437323 | 12 | PI567371B | 11 | PI561372 | 10 |
| 3 | PI437345 | 11 | PI594616 | 12 | PI567287 | 11 | PI476890 | 10 |
| 4 | PI232987 | 11 | PI594714 | 12 | PI594501A | 11 | PI567316A | 11 |
| 5 | PI65388 | 11 | PI483464A | 12 | PI594615 | 11 | PI594881 | 11 |
| 6 | PI548168 | 11 | PI483464B | 12 | PI567395 | 12 | PI567308 | 11 |
| 7 | PI81770 | 11 | PI606374 | 12 | PI606407 | 12 | PI567310B | 11 |
| 8 | PI135590 | 11 | PI70469-1 | 12 | PI605846F | 12 | PI567355A | 11 |
| 9 | PI437656 | 11 | PI70515 | 12 | PI606436 | 12 | PI567328 | 11 |
| 10 | PI437663 | 11 | PI68806 | 12 | PI632939 | 12 | PI594660D | 11 |
| Rank | MG VI | Predicted CW score | MG VII | Predicted CW score | MG VIII | Predicted CW score | MG IX | Predicted CW score |
| 1 | PI603629A | 9 | PI603524 | 8 | PI567021 | 10 | PI239236 | 10 |
| 2 | PI603629B | 10 | PI594498B | 9 | PI441355 | 10 | PI323563 | 10 |
| 3 | PI567406A | 10 | PI594498A | 11 | PI567108B | 10 | PI459025C | 10 |
| 4 | PI212604 | 10 | PI594495 | 11 | PI548489 | 10 | PI594760B | 11 |
| 5 | PI222397 | 10 | PI603631 | 11 | PI374171 | 11 | PI587911B | 11 |
| 6 | PI269518A | 11 | PI594500D | 11 | PI603626 | 11 | PI307853 | 11 |
| 7 | PI567326A | 11 | PI594502 | 11 | PI603737B | 11 | PI587911C | 11 |
| 8 | PI468130 | 11 | PI594458A | 11 | PI603722 | 11 | PI566973 | 11 |
| 9 | PI615508 | 11 | PI567076 | 11 | PI603737C | 11 | PI497963 | 11 |
| 10 | PI219732 | 11 | PI567068A | 11 | PI594756 | 11 | PI603740B | 11 |
| Rank | MG X | Predicted CW score |  |  |  |  |  |  |
| 1 | PI588000 | 9 |  |  |  |  |  |  |
| 2 | PI597468 | 12 |  |  |  |  |  |  |
| 3 | PI281895B | 12 |  |  |  |  |  |  |
| 4 | PI307868 | 12 |  |  |  |  |  |  |
| 5 | PI307880C | 12 |  |  |  |  |  |  |
| 6 | PI587934 | 12 |  |  |  |  |  |  |
| 7 | PI307880E | 12 |  |  |  |  |  |  |
| 8 | PI578361 | 13 |  |  |  |  |  |  |
| 9 | PI281895A | 13 |  |  |  |  |  |  |
| 10 | PI588046 | 13 |  |  |  |  |  |  |

**Table S5.** Predicted slowest and fastest wilting accessions of maturity group (MG) 000 through X using genomic estimated breeding values (GEBVs) from the USDA Soybean Germplasm Collection (Song et al., 2015). The training set for the prediction of 19,648 accessions was determined using the averaged canopy wilting (CW) scores of 100 new accessions in the present study, 373 accessions reported by Kaler et al. (2017a), and of the 153 accessions reported by the Steketee et al. (2020).

| **Top 10 fastest wilting accessions** | | | | | | | | |
| --- | --- | --- | --- | --- | --- | --- | --- | --- |
| Rank | MG 000 | Predicted CW score | MG 00 | Predicted CW score | MG 0 | Predicted CW score | MG I | Predicted CW score |
| 1 | PI546046 | 24 | PI567174A | 25 | PI361085B | 28 | PI378655 | 29 |
| 2 | PI546049 | 25 | PI458531 | 25 | PI437590 | 28 | PI417295 | 29 |
| 3 | PI546047 | 25 | PI593951 | 26 | PI634898 | 28 | PI189881 | 29 |
| 4 | PI449458A | 25 | PI592916 | 26 | PI417519A | 28 | FC30233 | 29 |
| 5 | PI546050 | 25 | PI567200B | 26 | PI372424 | 28 | PI437612 | 29 |
| 6 | PI548594 | 25 | PI546044 | 26 | PI297532 | 28 | PI548238 | 29 |
| 7 | PI546051 | 25 | PI442023 | 26 | PI437191 | 28 | PI253652C | 29 |
| 8 | PI470929 | 25 | PI597417 | 26 | PI290131 | 29 | PI417519B | 29 |
| 9 | PI546048 | 25 | PI546043 | 27 | PI548621 | 29 | PI248406 | 29 |
| 10 | PI603316 | 26 | PI347561 | 29 | PI438351 | 29 | PI437405 | 29 |
| Rank | MG II | Predicted CW score | MG III | Predicted CW score | MG IV | Predicted CW score | MG V | Predicted CW score |
| 1 | PI547741 | 28 | PI547784 | 27 | PI547749 | 28 | PI170895 | 27 |
| 2 | PI547759 | 28 | PI547682 | 27 | PI591545 | 28 | PI547774 | 27 |
| 3 | PI506930 | 28 | PI88466 | 27 | PI547769 | 28 | PI547775 | 28 |
| 4 | PI547732 | 28 | PI416750 | 27 | PI547786 | 28 | PI381667 | 28 |
| 5 | PI547742 | 29 | PI547689 | 27 | PI547702 | 28 | PI381666 | 28 |
| 6 | PI84954 | 29 | PI253665D | 28 | PI547757 | 28 | PI381668 | 28 |
| 7 | PI90180 | 29 | PI547694 | 28 | PI547748 | 28 | PI381670 | 29 |
| 8 | PI437722 | 29 | PI80480 | 28 | PI547707 | 28 | PI547793 | 29 |
| 9 | PI91167 | 29 | PI91153 | 28 | PI547767 | 29 | PI381664 | 30 |
| 10 | PI393999 | 29 | PI548230 | 28 | PI507467 | 31 | PI438344 | 31 |
| Rank | MG VI | Predicted CW score | MG VII | Predicted CW score | MG VIII | Predicted CW score | MG IX | Predicted CW score |
| 1 | PI201428 | 28 | PI644043 | 24 | PI507486 | 23 | PI423971A | 21 |
| 2 | PI159322 | 28 | PI628890 | 24 | PI423913 | 23 | PI505649A | 21 |
| 3 | PI381679 | 28 | FC31707 | 24 | PI628891 | 23 | PI341241B | 21 |
| 4 | PI221713 | 28 | PI548979 | 25 | PI324068 | 23 | PI341247 | 21 |
| 5 | PI170892 | 28 | PI601984 | 25 | PI548970 | 23 | PI417118 | 21 |
| 6 | PI209908 | 28 | PI438347 | 26 | PI500648 | 23 | PI628946 | 22 |
| 7 | PI170889 | 29 | PI159097 | 27 | PI245007 | 23 | PI628942 | 22 |
| 8 | PI170888 | 31 | PI210352 | 27 | PI572237 | 24 | PI613056 | 23 |
| 9 | PI381684 | 31 | PI381672 | 27 | PI200832 | 24 | PI341242 | 23 |
| 10 | PI381665 | 33 | PI210353 | 28 | PI548697 | 24 | PI594598B | 25 |
| Rank | MG X | Predicted CW score |  |  |  |  |  |  |
| 1 | PI587952 | 16 |  |  |  |  |  |  |
| 2 | PI274454 | 17 |  |  |  |  |  |  |
| 3 | PI594724 | 17 |  |  |  |  |  |  |
| 4 | PI587945 | 17 |  |  |  |  |  |  |
| 5 | PI587939 | 17 |  |  |  |  |  |  |
| 6 | PI587947 | 17 |  |  |  |  |  |  |
| 7 | PI587963 | 17 |  |  |  |  |  |  |
| 8 | PI587938 | 17 |  |  |  |  |  |  |
| 9 | PI587953 | 18 |  |  |  |  |  |  |
| 10 | PI613055 | 22 |  |  |  |  |  |  |

| **Table S6.** List of significant coincident SNPs associated with canopy wilting and their potential candidate genes based on 87 identified SNPs from twelve environments for the irrigated and drought treatments, averaged over irrigated treatments (Ave_IR), average over drought treatments (Ave_DR), and averaged across all environments (AAE). | | | | | | | |
| --- | --- | --- | --- | --- | --- | --- | --- |
| SNP_ID | Distance from SNP | Gene | Start | End | Biological process | Molecular function | Cellular component |
| **Irrigated (IR)** | | | | | | | |
| ss715580748 | Within gene | Glyma01g00940 | 588161 | 598604 | purine nucleotide biosynthetic process, embryo development ending in seed dormancy, seed dormancy process, regulation of cell cycle process, leaf development, cotyledon development | nucleotide binding, aminoacyl-tRNA ligase activity, arginine-tRNA ligase activity, ATP binding | cytoplasm, mitochondrion, chloroplast, chloroplast stroma |
| ss715581576 | 1612 | Glyma02g03080 | 2389389 | 2394897 | glycolysis, metabolic process, oxidation-reduction process | pyruvate dehydrogenase (acetyl-transferring) activity, oxidoreductase activity, acting on the aldehyde or oxo group of donors, disulfide as acceptor | nucleus, mitochondrion, cytosol, intracellular membrane-bounded organelle |
| ss715584835 | 9108 | Glyma03g02580 | 2347725 | 2350697 | transition metal ion transport, photomorphogenesis, skotomorphogenesis, vegetative to reproductive phase transition of meristem, regulation of anion channel activity, brassinosteroid biosynthetic process | AT DNA binding | nucleus |
| ss715589557 | Within gene | Glyma04g10693 | 8916084 | 8937155 | polysaccharide catabolic process, carbohydrate metabolic process, starch metabolic process, starch catabolic process, plant-type cell wall organization, starch biosynthetic process | catalytic activity, hydrolase activity, hydrolyzing O-glycosyl compounds, alpha-amylase activity, isoamylase activity, cation binding | chloroplast, chloroplast starch grain, chloroplast stroma |
| ss715590117 | 30 | Glyma05g02600 | 1983800 | 1986652 | maltose metabolic process; sterol starch, pentacyclic triterpenoid, glucosinolate biosynthetic process; oxidation-reduction process | ferric-chelate reductase activity, iron ion binding, electron carrier activity, oxidoreductase activity, flavin adenine dinucleotide binding | mitochondrion, plasma membrane |
| ss715591559 | 309 | Glyma05g33080 | 37833046 | 37839669 | biological process | molecular function | extracellular region, plasma membrane |
| ss715598790 | 1 | Glyma07g09810 | 8267192 | 8270892 | tryptophan biosynthetic process | molecular function | cell wall, mitochondrion, endoplasmic reticulum, plasmodesma |
| ss715602950 | Within gene | Glyma09g01670 | 1065749 | 1067871 | regulation of transcription, DNA-dependent | sequence-specific DNA binding transcription factor activity | nucleus |
| ss715605115 | 4479 | Glyma09g06300 | 5061787 | 5064418 | chloroplast organization, iron-sulfur cluster assembly | structural molecule activity, iron ion binding, iron-sulfur cluster binding | chloroplast, chloroplast stroma |
| ss715606242 | 921 | Glyma10g04290 | 3254325 | 3257392 | sterol biosynthetic process, plant-type cell wall cellulose metabolic process, cell wall pectin metabolic process | transporter activity | intracellular, nucleus, cytosol, plasma membrane |
| ss715611329 | 6247 | Glyma11g13880 | 9894777 | 9905940 | calcium ion transport, response to desiccation, response to cold, response to water deprivation, response to light stimulus, response to wounding, response to salt stress, jasmonic acid biosynthetic process, response to abscisic acid stimulus, starch biosynthetic process, response to herbivore | protein binding, lipoxygenase activity | cytoplasm, chloroplast |
| ss715612746 | 33370 | Glyma12g34175 | 37316428 | 37322750 | gibberellin metabolic process, oxidation-reduction process | oxidoreductase activity, C-20 gibberellin 2-beta-dioxygenase activity | cytoplasm |
| ss715614917 | 4752 | Glyma13g25740 | 28954588 | 28965263 | photoperiodism, flowering | molecular function | nucleus, mitochondrion |
| ss715617331 | Within gene | Glyma14g11520 | 10091952 | 10094519 | protein phosphorylation | protein serine/threonine kinase activity, ATP binding, transferase activity, transferring phosphorus-containing groups | plasma membrane |
| ss715620301 | Within gene | Glyma15g14740 | 11227645 | 11229017 | biological process | molecular function | extracellular region |
| ss715622635 | Within gene | Glyma15g42190 | 49579044 | 49583448 | - | - | chloroplast |
| ss715627700 | 2110 | Glyma17g36360 | 40331697 | 40333131 | response to wounding, response to mechanical stimulus, response to absence of light, salicylic acid mediated signaling pathway, jasmonic acid mediated signaling pathway, regulation of plant-type hypersensitive response, | molecular function | plasma membrane, chloroplast |
| ss715630654 | 42173 | Glyma18g38560 | 46153747 | 46155733 | regulation of transcription, DNA-dependent, xylem development, cell wall macromolecule metabolic process | DNA binding, sequence-specific DNA binding transcription factor activity, zinc ion binding | nucleus |
| ss715632103 | 6889 | Glyma18g49870 | 59169158 | 59171068 | response to wounding, phenylpropanoid metabolic process, coumarin biosynthetic process, lignin biosynthetic process, positive regulation of flavonoid biosynthetic process, sterol biosynthetic process | luteolin O-methyltransferase activity, quercetin 3-O-methyltransferase activity, myricetin 3'-O-methyltransferase activity, caffeate O-methyltransferase activity | nucleus, cytoplasm, cytosol |
| ss715632312 | 1186 | Glyma18g51820 | 60576030 | 60579116 | mRNA export from nucleus, cell adhesion, post-embryonic morphogenesis, regulation of flower development, polarity specification of adaxial/abaxial axis, leaf morphogenesis, regulation of meristem growth, regulation of root meristem growth, trichome morphogenesis, actin nucleation, root hair cell differentiation, ovule development, cell wall organization | ATP binding, transferase activity, transferring phosphorus-containing groups, receptor serine/threonine kinase binding | plasma membrane |
| ss715632502 | 7577 | Glyma18g53810 | 62064546 | 62072118 | protein phosphorylation, protein N-linked glycosylation, | protein kinase activity, protein serine/threonine kinase activity, ATP binding, transferase activity, transferring phosphorus-containing groups | nucleus |
| ss715634991 | 1645 | Glyma19g32480 | 40215520 | 40218586 | DNA-dependent DNA replication, protein folding, petal formation, sepal formation | heat shock protein binding, unfolded protein binding | cytoplasm |
| ss715635419 | 3388 | Glyma19g37870 | 44959300 | 44961265 | autophagy, fatty acid catabolic process, adaxial/abaxial axis specification, regulation of meristem growth, leaf vascular tissue pattern formation, leaf shaping, negative regulation of sequence-specific DNA binding transcription factor activity | protein binding | nucleus |
| ss715635420 | 3043 | Glyma19g37880 | 44967085 | 44968539 | vesicle-mediated transport | molecular function | chloroplast, membrane |
| ss715635421 | 648 | Glyma19g37880 | 44967085 | 44968539 | vesicle-mediated transport | molecular function | chloroplast, membrane |
| ss715635422 | 5611 | Glyma19g37890 | 44979743 | 44981657 | protein targeting to vacuole, response to sucrose stimulus, regulation of flower development, photoperiodism, flowering, negative regulation of cell aging | transcription cofactor activity, phosphatidylethanolamine binding | nucleus, cytoplasm, vacuole, plasma membrane, vesicle |
| ss715635432 | Within gene | Glyma19g38010 | 45045959 | 45049471 | biological process | - | chloroplast, plastoglobule |
| ss715635433 | 3441 | Glyma19g38040 | 45065689 | 45068369 | N-terminal protein myristoylation, cell redox homeostasis, anther development | electron carrier activity, protein disulfide oxidoreductase activity | nucleus, plasmodesma |
| ss715635435 | 902 | Glyma19g38040 | 45065689 | 45068369 | N-terminal protein myristoylation, cell redox homeostasis, anther development | electron carrier activity, protein disulfide oxidoreductase activity | nucleus, plasmodesma |
| ss715635436 | Within gene | Glyma19g38040 | 45065689 | 45068369 | N-terminal protein myristoylation, cell redox homeostasis, anther development | electron carrier activity, protein disulfide oxidoreductase activity | nucleus, plasmodesma |
| ss715635437 | Within gene | Glyma19g38040 | 45065689 | 45068369 | N-terminal protein myristoylation, cell redox homeostasis, anther development | electron carrier activity, protein disulfide oxidoreductase activity | nucleus, plasmodesma |
| ss715635439 | 3179 | Glyma19g38060 | 45075847 | 45076726 | ethylene biosynthetic process | molecular function | nucleus, plasma membrane |
| ss715635442 | 3935 | Glyma19g38060 | 45075847 | 45076726 | ethylene biosynthetic process | molecular function | nucleus, plasma membrane |
| ss715635447 | Within gene | Glyma19g38085 | 45095328 | 45102492 | biological process | nucleotide binding, RNA binding | cytosol |
| ss715635448 | Within gene | Glyma19g38085 | 45095328 | 45102492 | biological process | nucleotide binding, RNA binding | cytosol |
| ss715639090 | 16516 | Glyma20g00270 | 76450 | 80091 | glucuronoxylan metabolic process, xylan biosynthetic process, phosphatidylinositol phosphorylation | hydrolase activity | nucleus |
| ss715636929 | Within gene | Glyma20g02500 | 2105970 | 2116545 | - | catalytic activity | chloroplast |
| ss715636930 | Within gene | Glyma20g02500 | 2105970 | 2116545 | - | catalytic activity | chloroplast |
| **Drought (DR)** | | | | | | | |
| ss715580188 | Within gene | Glyma01g39110 | 51090472 | 51105666 | - | nucleotide binding | plasma membrane |
| ss715580224 | Within gene | Glyma01g39460 | 51397148 | 51399175 | response to wounding, phenylpropanoid metabolic process, coumarin biosynthetic process, positive regulation of flavonoid biosynthetic process, cysteine biosynthetic process | caffeoyl-CoA O-methyltransferase activity | nucleus, cytosol |
| ss715582745 | Within gene | Glyma02g05550 | 4438431 | 4443610 | protein metabolic process, cellular protein modification process, oxidation-reduction process | peptide-methionine (S)-S-oxide reductase activity | chloroplast |
| ss715582949 | 1783 | Glyma02g05760 | 4613631 | 4618879 | metabolic process, response to mechanical stimulus, calcium-mediated signaling, cellular response to water deprivation, oxidation-reduction process | aldehyde dehydrogenase [NAD(P)+] activity, oxidoreductase activity | cytoplasm, endoplasmic reticulum |
| ss715584310 | 615 | Glyma02g11701 | 9895291 | 9896037 | metabolic process | abscisic acid glucosyltransferase activity | chloroplast |
| ss715580895 | 3047 | Glyma02g11850 | 10067471 | 10072083 | response to red or far red light, positive regulation of cell proliferation, positive regulation of flower development, embryo development ending in seed dormancy, endosperm development | ubiquitin-protein ligase activity, protein binding | nucleus, cytosol |
| ss715592981 | 2783 | Glyma06g16620 | 13002305 | 13008808 | - | chromatin binding, Ran GTPase binding | - |
| ss715593221 | 2803 | Glyma06g17990 | 14313112 | 14318337 | cytokinesis, acetyl-CoA metabolic process, pinocytosis, positive gravitropism, sterol biosynthetic process, brassinosteroid biosynthetic process | 0 | mitochondrion, endoplasmic reticulum |
| ss715594542 | 666 | Glyma06g42750 | 46075330 | 46077639 | response to ethylene stimulus, defense response to fungus, incompatible interaction, leaf senescence | cysteine-type peptidase activity | extracellular region, senescence-associated vacuole |
| ss715595238 | Within gene | Glyma06g47640 | 50038162 | 50043689 | calcium ion transport, iron ion transport, response to wounding, response to bacterium, response to salt stress, phenylpropanoid metabolic process, coumarin biosynthetic process, cellular response to iron ion starvation, response to nitrate, brassinosteroid biosynthetic process, cellular modified amino acid biosynthetic process, cellular response to gibberellin stimulus | hydrolase activity, hydrolyzing O-glycosyl compounds, beta-fructofuranosidase activity, sucrose alpha-glucosidase activity | vacuole |
| ss715597447 | 4488 | Glyma07g31710 | 36683232 | 36683888 | regulation of transcription, DNA-dependent, defense response to fungus | sequence-specific DNA binding transcription factor activity | nucleus |
| ss715606242 | 921 | Glyma10g04290 | 3254325 | 3257392 | sterol biosynthetic process, plant-type cell wall cellulose metabolic process, cell wall pectin metabolic process | transporter activity | intracellular, nucleus, cytosol, plasma membrane |
| ss715611163 | 5386 | Glyma11g11160 | 7940007 | 7943524 | gibberellin metabolic process, oxidation-reduction process | oxidoreductase activity, C-20 gibberellin 2-beta-dioxygenase activity | cytoplasm |
| ss715611278 | Within gene | Glyma11g12720 | 9062945 | 9066126 | transport, negative regulation of defense response, amino acid import, transmembrane transport | transporter activity | plasma membrane |
| ss715610265 | 7039 | Glyma11g34690 | 36473267 | 36476976 | xylem development, cell wall macromolecule metabolic process | Rho guanyl-nucleotide exchange factor activity | nucleus |
| ss715611782 | Within gene | Glyma12g03260 | 2143449 | 2148398 | - | nucleotide binding, RNA binding, zinc ion binding | intracellular |
| ss715612746 | 33370 | Glyma12g34175 | 37316428 | 37322750 | gibberellin metabolic process, oxidation-reduction process | oxidoreductase activity, C-20 gibberellin 2-beta-dioxygenase activity | cytoplasm |
| ss715620072 | 106 | Glyma14g01580 | 912562 | 915836 | N-terminal protein myristoylation, transmembrane transport | - | membrane |
| ss715618057 | 5700 | Glyma14g03560 | 2316858 | 2319297 | response to cold, detection of biotic stimulus, response to blue light, response to high light intensity, salicylic acid biosynthetic process, response to sucrose stimulus, defense response, incompatible interaction, systemic acquired resistance, salicylic acid mediated signaling pathway, jasmonic acid mediated signaling pathway, response to red light, response to far red light, regulation of hydrogen peroxide metabolic process, regulation of plant-type hypersensitive response, photosynthesis, light reaction | calcium ion binding, poly(U) RNA binding | chloroplast, chloroplast thylakoid |
| ss715618585 | 6356 | Glyma14g05390 | 3825605 | 3827583 | salicylic acid biosynthetic process, jasmonic acid mediated signaling pathwaysystemic acquired resistance, salicylic acid mediated signaling pathway, negative regulation of defense response, oxidation-reduction process | oxidoreductase activity | cytoplasm |
| ss715623062 | 1509 | Glyma15g10620 | 7708994 | 7717091 | cell morphogenesis, regulation of transcription, DNA-dependent, tissue development, organ morphogenesis, trichome morphogenesis, xylan biosynthetic process, root hair cell differentiation, hydrogen peroxide biosynthetic process | sequence-specific DNA binding transcription factor activity | nucleus |
| ss715624261 | 1245 | Glyma16g25880 | 30030722 | 30034925 | response to light stimulus | signal transducer activity | plasma membrane |
| ss715628231 | Within gene | Glyma17g10970 | 8247753 | 8249783 | regulation of transcription, DNA-dependent | sequence-specific DNA binding transcription factor activity | nucleus |
| ss715626991 | 1700 | Glyma17g33001 | 36587878 | 36589637 | - | - | - |
| ss715632594 | Within gene | Glyma18g01340 | 701671 | 703255 | leaf senescence | - | mitochondrion |
| ss715632103 | 6889 | Glyma18g49870 | 59169158 | 59171068 | response to wounding, phenylpropanoid metabolic process, coumarin biosynthetic process, lignin biosynthetic process, positive regulation of flavonoid biosynthetic process, sterol biosynthetic process | luteolin O-methyltransferase activity, quercetin 3-O-methyltransferase activity, myricetin 3'-O-methyltransferase activity, caffeate O-methyltransferase activity | nucleus, cytoplasm, cytosol |
| ss715633103 | 1520 | Glyma19g01640 | 1254030 | 1256760 | signal transduction, response to water deprivation, response to wounding, response to ethylene stimulus, response to auxin stimulus, response to abscisic acid stimulus, abscisic acid mediated signaling pathway, response to jasmonic acid stimulus, salicylic acid mediated signaling pathway, jasmonic acid mediated signaling pathway, heat acclimation, regulation of plant-type hypersensitive response, hyperosmotic salinity response | zinc ion binding | nucleus |
| ss715635396 | Within gene | Glyma19g37600 | 44700483 | 44706639 | DNA recombination, response to gamma radiation, regulation of telomere maintenance | zinc ion binding | intracellular, nucleus |
| ss715635416 | 2386 | Glyma19g37850 | 44941217 | 44944989 | cellular response to phosphate starvation, galactolipid biosynthetic process, negative regulation of transcription, DNA-dependent | protein serine/threonine phosphatase activity, hydrolase activity, metal ion binding | extracellular region |
| ss715635458 | 1450 | Glyma19g38211 | 45179582 | 45181898 | regulation of transcription, DNA-dependent, multicellular organismal development, response to chitin | sequence-specific DNA binding transcription factor activity | nucleus |
| ss715638951 | Within gene | Glyma20g39460 | 46731582 | 46756418 | - | - | cytoplasm |
| **Average irrigated (Ave_IR)** | | | | | | | |
| ss715614827 | 481 | Glyma13g25150 | 28423018 | 28424910 | - | - | - |
| ss715614963 | 3756 | Glyma13g25900 | 29134000 | 29134994 | response to ethylene stimulus, response to abscisic acid stimulus, response to gibberellin stimulus, salicylic acid mediated signaling pathway, jasmonic acid mediated signaling pathway, positive regulation of gibberellic acid mediated signaling pathway, seed dormancy process, raffinose family oligosaccharide biosynthetic process, hyperosmotic salinity response, floral organ morphogenesis | protein binding, hydrolase activity | nucleus |
| ss715615031 | Within gene | Glyma13g26380 | 29563650 | 29569057 | defense response, systemic acquired resistance, salicylic acid biosynthetic process, | protein binding, ADP binding | extracellular region |
| ss715617562 | 5043 | Glyma14g02050 | 1212132 | 1214421 | protein transport | P-P-bond-hydrolysis-driven protein transmembrane transporter activity | mitochondrion |
| ss715632103 | 6889 | Glyma18g49870 | 59169158 | 59171068 | response to wounding, phenylpropanoid metabolic process, coumarin biosynthetic process, lignin biosynthetic process, positive regulation of flavonoid biosynthetic process, sterol biosynthetic process | luteolin O-methyltransferase activity, quercetin 3-O-methyltransferase activity, myricetin 3'-O-methyltransferase activity, caffeate O-methyltransferase activity | nucleus, cytoplasm, cytosol |
| ss715635361 | Within gene | Glyma19g37260 | 44462493 | 44468225 | N-terminal protein myristoylation, proteolysis, fatty acid beta-oxidation, protein import into peroxisome matrix | aspartic-type endopeptidase activity | extracellular region |
| **Average drought (Ave_DR)** | | | | | | | |
| ss715580947 | Within gene | Glyma02g01480 | 1064976 | 1073152 | protein phosphorylation | protein serine/threonine kinase activity | plasma membrane |
| ss715588382 | 6258 | Glyma04g37330 | 43690674 | 43693491 | stomatal complex morphogenesis, glucosinolate biosynthetic process | - | chloroplast, thylakoid |
| ss715588984 | Within gene | Glyma04g42970 | 48591142 | 48595154 | - | - | extracellular region, membrane |
| ss715591524 | Within gene | Glyma05g32756 | 37589565 | 37596078 | response to hypoxia, defense response to insect, respiratory burst involved in defense response, regulation of transcription, DNA-dependent, response to cold, detection of external stimulus, detection of biotic stimulus, systemic acquired resistance, salicylic acid biosynthetic process, abscisic acid mediated signaling pathway, jasmonic acid mediated signaling pathway, leaf senescence, regulation of hydrogen peroxide metabolic process, regulation of plant-type hypersensitive response, regulation of defense response | triglyceride lipase activity, protein binding | nucleus |
| ss715598277 | 2148 | Glyma07g06210 | 4923256 | 4928500 | response to osmotic stress, biosynthetic process, response to abscisic acid stimulus, plant-type cell wall biogenesis | transferase activity, transferring glycosyl groups | endoplasmic reticulum membrane |
| ss715599784 | 32218 | Glyma08g21370 | 16213265 | 16218310 | root epidermal cell differentiation, glycolysis | 6-phosphofructokinase activity, ATP binding | cytoplasm, cytosol |
| ss715604779 | Within gene | Glyma09g38640 | 43955759 | 43959448 | glucose catabolic process, cellulose biosynthetic process, golgi vesicle transport | - | Golgi apparatus |
| ss715632103 | 6889 | Glyma18g49870 | 59169158 | 59171068 | response to wounding, phenylpropanoid metabolic process, coumarin biosynthetic process, lignin biosynthetic process, positive regulation of flavonoid biosynthetic process, sterol biosynthetic process | luteolin O-methyltransferase activity, quercetin 3-O-methyltransferase activity, myricetin 3'-O-methyltransferase activity, caffeate O-methyltransferase activity | nucleus, cytoplasm, cytosol |
| **Average across environments (AAE)** | | | | | | | |
| ss715590862 | 23656 | Glyma05g27250 | 33153928 | 33156290 | response to water deprivation, abscisic acid biosynthetic process, seed dormancy process | 9-cis-epoxycarotenoid dioxygenase activity | chloroplast, thylakoid |
| ss715598845 | 6565 | Glyma07g10180 | 8509838 | 8511558 | response to hypoxia, regulation of transcription, DNA-dependent, systemic acquired resistance, salicylic acid mediated signaling pathway, regulation of hydrogen peroxide metabolic process | - | nucleus |
| ss715611329 | 6247 | Glyma11g13880 | 9894777 | 9905940 | calcium ion transport, response to desiccation, response to cold, response to water deprivation, response to light stimulus, response to wounding, response to salt stress, jasmonic acid biosynthetic process, response to abscisic acid stimulus, starch biosynthetic process, response to herbivore | protein binding, lipoxygenase activity | cytoplasm, chloroplast |
| ss715632103 | 6889 | Glyma18g49870 | 59169158 | 59171068 | response to wounding, phenylpropanoid metabolic process, coumarin biosynthetic process, lignin biosynthetic process, positive regulation of flavonoid biosynthetic process, sterol biosynthetic process | luteolin O-methyltransferase activity, quercetin 3-O-methyltransferase activity, myricetin 3'-O-methyltransferase activity, caffeate O-methyltransferase activity | nucleus, cytoplasm, cytosol |

**Table S7.** Significant novel SNPs associated with canopy wilting and their potential candidate genes based on 101 identified SNPs from twelve environments under irrigated and drought treatments, averaged across irrigated treatments (Ave_IR), averaged across drought treatments (Ave_DR), and averaged across all environments (AAE).

| SNP_ID | Distance from SNP | Gene | Start | End | Biological process | Molecular function | Cellular component |
| --- | --- | --- | --- | --- | --- | --- | --- |
| **Irrigated (IR)** | | | | | | | |
| ss715579037 | Within gene | Glyma01g03850 | 3312868 | 3318968 | potassium ion transport, potassium ion transmembrane transport | potassium ion transmembrane transporter activity | plasma membrane, membrane |
| ss715582188 | 1672 | Glyma02g05010 | 4077995 | 4078612 | transition metal ion transport, iron ion transport, cellular response to iron ion starvation, response to nitrate, nitrate transport | manganese ion binding, nutrient reservoir activity | extracellular region, apoplast |
| ss715583267 | 535 | Glyma02g06140 | 4909888 | 4910244 | biological process | molecular function | mitochondrion |
| ss715583177 | Within gene | Glyma02g43530 | 48285052 | 48289281 | carbohydrate metabolic process, lactose catabolic process, N-terminal protein myristoylation, oligosaccharide metabolic process, glycoside catabolic process, glycosylceramide catabolic process | catalytic activity, hydrolase activity, hydrolyzing O-glycosyl compounds, alpha-galactosidase activity | extracellular region, cell wall, cytoplasm, plant-type cell wall |
| ss715589211 | Within gene | Glyma04g08870 | 6979042 | 6980040 | biological process | catalytic activity | Golgi apparatus, membrane |
| ss715599166 | Within gene | Glyma08g14170 | 10303663 | 10312295 | positive regulation of cell proliferation | molecular function | chloroplast, integral to membrane |
| ss715601604 | 1761 | Glyma08g38950 | 38204926 | 38213434 | transmembrane transport | transporter activity | vacuolar membrane, plasma membrane |
| ss715603362 | 22862 | Glyma09g19520 | 24210270 | 24215862 | - | - | - |
| ss715603732 | Within gene | Glyma09g28780 | 35727928 | 35730537 | biological process | molecular function | mitochondrion |
| ss715604821 | Within gene | Glyma09g39441 | 44520921 | 44524577 | photosystem II assembly, myo-inositol hexakisphosphate biosynthetic process | molecular function | chloroplast |
| ss715609311 | 4609 | Glyma11g02480 | 1609996 | 1613274 | response to osmotic stress, salicylic acid biosynthetic process, salicylic acid mediated signaling pathway, nitrate transport, negative regulation of defense response, innate immune response, root hair elongation | - | nucleus |
| ss715613244 | Within gene | Glyma12g08390 | 6150009 | 6152455 | maltose metabolic process, Thylakoid membrane organization, starch biosynthetic process | molecular function | chloroplast |
| ss715612970 | Within gene | Glyma12g36380 | 39462700 | 39466643 | response to abscisic acid stimulus, embryo development ending in seed dormancy, flavonoid biosynthetic process, cell wall modification involved in abscission, seed germination, seed dormancy process, organ senescence, oxidation-reduction process | oxidoreductase activity | cytoplasm |
| ss715616824 | Within gene | Glyma13g05441 | 5761151 | 5775850 | response to hypoxia, amylopectin biosynthetic process, myo-inositol hexakisphosphate biosynthetic process, galactolipid biosynthetic process | starch synthase activity, transferase activity, transferring glycosyl groups | chloroplast |
| ss715614097 | 4011 | Glyma13g18780 | 22437753 | 22442119 | plant-type cell wall biogenesis, secondary cell wall biogenesis, glucuronoxylan metabolic process, cellulose biosynthetic process, cell wall biogenesis, defense response to bacterium, cell wall macromolecule metabolic process, xylan biosynthetic process, defense response to fungus | transferase activity, transferring glycosyl groups, cellulose synthase activity | plasma membrane |
| ss715620897 | 8076 | Glyma15g18340 | 15067545 | 15073577 | protein phosphorylation | protein serine/threonine kinase activity | plasma membrane |
| ss715621830 | 18381 | Glyma15g35460 | 40129555 | 40141953 | proteolysis, metabolic process, negative regulation of catalytic activity | serine-type endopeptidase activity, identical protein binding | extracellular region, cell wall, cytoplasm, plant-type cell wall, apoplast |
| ss715621869 | 9857 | Glyma15g36285 | 41413413 | 41417159 | biological process, vernalization response, anthocyanin accumulation in tissues in response to UV light, carpel development | molecular function | nucleus |
| ss715621873 | 20221 | Glyma15g36320 | 41550002 | 41550681 | - | - | - |
| ss715621877 | 28953 | Glyma15g36445 | 41740774 | 41741492 | - | - | - |
| ss715623751 | 5930 | Glyma16g02730 | 2326209 | 2329930 | respiratory burst involved in defense response, cell communication, response to cold, response to wounding, abscisic acid mediated signaling pathway, systemic acquired resistance, salicylic acid mediated signaling pathway, jasmonic acid mediated signaling pathway, regulation of plant-type hypersensitive response, negative regulation of defense response, hyperosmotic salinity response, negative regulation of programmed cell death, defense response to fungus | 1-phosphatidylinositol 4-kinase activity, phosphotransferase activity, alcohol group as acceptor | cytosol, chloroplast |
| ss715626698 | 3946 | Glyma17g00630 | 301887 | 307625 | regulation of transcription, DNA-dependent | DNA binding, zinc ion binding, methylated histone residue binding | nucleus, cytosol |
| ss715632608 | Within gene | Glyma18g08380 | 7136346 | 7140504 | - | molecular function | nucleus, cytosol |
| ss715633673 | 24395 | Glyma19g24451 | 30045006 | 30045828 | regulation of transcription, DNA-dependent, response to ethylene stimulus, response to auxin stimulus, response to sucrose stimulus, flavonoid biosynthetic process, response to UV-B | DNA binding, sequence-specific DNA binding transcription factor activity | nucleus |
| ss715636922 | - | - | - | - | - | - | - |
| ss715636931 | - | - | - | - | - | - | - |
| ss715636938 | 10577 | Glyma20g15480 | 21304467 | 21306653 | tryptophan catabolic process, indoleacetic acid biosynthetic process, glucosinolate biosynthetic process, oxidation-reduction process | iron ion binding, electron carrier activity, oxidoreductase activity, acting on paired donors, with incorporation or reduction of molecular oxygen, NAD(P)H as one donor, and incorporation of one atom of oxygen | - |
| ss715636958 | 923 | Glyma20g16030 | 22122475 | 22131543 | nucleotide biosynthetic process | - | nucleus |
| ss715637018 | Within gene | Glyma20g16880 | 23675018 | 23700020 | regulation of gene expression by genetic imprinting, negative regulation of flower development, vernalization response, histone methylation | DNA binding, sequence-specific DNA binding transcription factor activity, protein binding, zinc ion binding | intracellular, nucleus |
| ss715637021 | 8070 | Glyma20g16920 | 23762132 | 23763201 | regulation of transcription, DNA-dependent, ethylene mediated signaling pathway | DNA binding, sequence-specific DNA binding transcription factor activity | nucleus |
| ss715637031 | 1205 | Glyma20g16980 | 23943200 | 23945969 | phosphatidylglycerol biosynthetic process, metabolic process, cutin biosynthetic process | transferase activity, transferring acyl groups | mitochondrion |
| ss715637033 | Within gene | Glyma20g17010 | 23971533 | 23979508 | nucleus organization, poly(A)+ mRNA export from nucleus | protein binding | nucleus |
| ss715637037 | 5455 | Glyma20g17020 | 23983441 | 23991384 | protein phosphorylation, N-terminal protein myristoylation, protein autophosphorylation | protein serine/threonine kinase activity, calmodulin-dependent protein kinase activity, calcium ion binding, ATP binding | peroxisome, chloroplast |
| ss715637047 | 22666 | Glyma20g17090 | 24154076 | 24155465 | glucose catabolic process, ubiquitin-dependent protein catabolic process | ubiquitin thiolesterase activity, ubiquitin-specific protease activity | nucleus, cytosol |
| ss715637052 | Within gene | Glyma20g17116 | 24164306 | 24221439 | ubiquitin-dependent protein catabolic process, gravitropism | ubiquitin thiolesterase activity, ubiquitin-specific protease activity | nucleus, cytosol, plasmodesma |
| ss715637062 | 8644 | Glyma20g17545 | 24579701 | 24581897 | water transport, vesicle docking involved in exocytosis, hyperosmotic response, response to temperature stimulus, response to salt stress, lateral root morphogenesis, root development, root hair elongation, auxin transport | protein binding | nucleus, cytosol, plasma membrane, chloroplast |
| ss715637093 | Within gene | Glyma20g18363 | 25744261 | 25746619 | - | - | - |
| **Drought (DR)** | | | | | | | |
| ss715578838 | 7879 | Glyma01g03120 | 2683532 | 2686001 | systemic acquired resistance, salicylic acid biosynthetic process, secondary metabolic process, oxidation-reduction process | oxidoreductase activity | - |
| ss715578860 | 2692 | Glyma01g03200 | 2741980 | 2744444 | oxidation-reduction process | copper ion binding, primary amine oxidase activity, quinone binding | peroxisome, chloroplast |
| ss715578432 | 58156 | Glyma01g09220 | 10985769 | 10988999 | systemic acquired resistance, regulation of defense response | monosaccharide transmembrane transporter activity | nucleus, cytoplasm, vacuole |
| ss715578452 | - | - | - | - | - | - | - |
| ss715586000 | Within gene | Glyma03g33360 | 40950549 | 40955184 | maltose metabolic process, histidine biosynthetic process, starch biosynthetic process | magnesium ion binding, ATP phosphoribosyltransferase activity | cytoplasm, chloroplast |
| ss715586264 | 5948 | Glyma03g37080 | 43844395 | 43846757 | folic acid-containing compound biosynthetic process, cysteine biosynthetic process, cellulose biosynthetic process, oxidation-reduction process | nucleotide binding, catalytic activity, methylenetetrahydrofolate dehydrogenase (NADP+) activity | cytoplasm, cytosol, chloroplast |
| ss715586270 | 1893 | Glyma03g37340 | 43989037 | 43990984 | translation, pyrimidine ribonucleotide biosynthetic process | structural constituent of ribosome, protein binding | intracellular, cytoplasm, vacuole, ribosome, chloroplast |
| ss715586272 | 2617 | Glyma03g37391 | 44013723 | 44016484 | response to cold, response to brassinosteroid stimulus, proline transport, cell wall modification | enzyme inhibitor activity, pectinesterase activity | extracellular region, cell wall |
| ss715589774 | Within gene | Glyma05g01560 | 1066172 | 1069154 | RNA methylation, protein targeting to mitochondrion, ribosome assembly | RNA binding | nucleus, cytosol |
| ss715598333 | 14575 | Glyma07g06700 | 5364136 | 5368108 | proximal/distal pattern formation, response to red light, floral organ abscission, flower morphogenesis | protein homodimerization activity | nucleus, cytoplasm |
| ss715599966 | 4914 | Glyma08g23240 | 17723740 | 17728797 | RNA-directed DNA methylation, | DNA binding, RNA binding, protein binding | cytoplasm, plasmodesma |
| ss715601844 | 8717 | Glyma08g40870 | 40722899 | 40727270 | transmembrane receptor protein tyrosine kinase signaling pathway | protein tyrosine kinase activity, ATP binding | plasma membrane |
| ss715601899 | 1581 | Glyma08g41120 | 41164809 | 41167327 | oxidation-reduction process | oxidoreductase activity | - |
| ss715601932 | Within gene | Glyma08g41570 | 41532326 | 41538080 | response to cold, response to water deprivation, response to abscisic acid stimulus, oxidation-reduction process | primary amine oxidase activity, polyamine oxidase activity | - |
| ss715603337 | Within gene | Glyma09g18490 | 22887169 | 22929537 | - | nucleic acid binding, RNA binding, ATP binding | nucleus |
| ss715604653 | 3111 | Glyma09g37400 | 42977614 | 42980092 | N-terminal protein myristoylation, cell redox homeostasis | electron carrier activity, protein disulfide oxidoreductase activity | nucleus, plasma membrane |
| ss715604845 | 1381 | Glyma09g39770 | 44784198 | 44797955 | potassium ion transport | potassium ion transmembrane transporter activity | chloroplast, plastid |
| ss715605772 | 30912 | Glyma10g15900 | 18621819 | 18627960 | response to flooding, oxidation-reduction process | oxidoreductase activity, nucleotide binding | cytoplasm, cytosol |
| ss715607829 | 7850 | Glyma10g40550 | 47935403 | 47938787 | regulation of plant-type hypersensitive response, defense response by callose deposition, negative regulation of programmed cell death, response to chitin | catalytic activity, protein serine/threonine phosphatase activity | - |
| ss715607984 | 558 | Glyma10g41811 | 48808516 | 48810799 | cytokinin biosynthetic process, response to fungus, protein phosphorylation | transmembrane receptor protein serine/threonine kinase activity, ATP binding | plasma membrane |
| ss715609728 | - | - | - | - | - | - | - |
| ss715611451 | 7557 | Glyma12g13980 | 12626430 | 12627935 | - | O-methyltransferase activity, protein dimerization activity | nucleus, cytosol |
| ss715612366 | 313 | Glyma12g30480 | 34100228 | 34102139 | lipid metabolic process | lipase activity, hydrolase activity, acting on ester bonds | extracellular region |
| ss715617261 | 488 | Glyma13g01120 | 850859 | 852473 | carbohydrate metabolic process, cellular glucan metabolic process | hydrolase activity, hydrolyzing O-glycosyl compounds, xyloglucan:xyloglucosyl transferase activity | cell wall, Golgi apparatus |
| ss715616860 | 2738 | Glyma13g05660 | 5988891 | 5991154 | - | - | nucleus |
| ss715616861 | 3823 | Glyma13g05660 | 5988891 | 5991154 | - | - | nucleus |
| ss715614911 | Within gene | Glyma13g25660 | 28877110 | 28895581 | photoperiodism, flowering | methyltransferase activity | nucleus |
| ss715619422 | 2977 | Glyma14g39190 | 48307505 | 48309299 | response to molecule of fungal origin | - | cytoplasm |
| ss715619486 | 427 | Glyma14g39740 | 48837023 | 48842461 | - | - | nucleus |
| ss715622900 | 5754 | Glyma15g08860 | 6264258 | 6266252 | regulation of transcription, DNA-dependent | sequence-specific DNA binding transcription factor activity | nucleus |
| ss715625245 | 285 | Glyma16g06555 | 5866738 | 5867189 | small GTPase mediated signal transduction | copper ion binding, GTP binding, | intracellular, Golgi apparatus, cytosol |
| ss715623895 | 13982 | Glyma16g23680 | 27535039 | 27538848 | response to other organism | - | extracellular region |
| ss715623938 | - | - | - | - | - | - | - |
| ss715626698 | 3946 | Glyma17g00630 | 301887 | 307625 | regulation of transcription, DNA-dependent | DNA binding, zinc ion binding, methylated histone residue binding | nucleus, cytosol |
| ss715626021 | Within gene | Glyma17g16540 | 13248862 | 13253091 | response to aluminum ion, stomatal movement | anion transmembrane transporter activity | endomembrane system |
| ss715626252 | 10243 | Glyma17g18320 | 15604190 | 15606296 | oxidation-reduction process | oxidoreductase activity | cellular component |
| ss715630859 | 1419 | Glyma18g39960 | 48315715 | 48319166 | metabolic process | catalytic activity, hydrolase activity | - |
| ss715631739 | 2667 | Glyma18g46350 | 56119982 | 56122096 | defense response, metabolic process, response to sucrose stimulus, flavonoid biosynthetic process, response to UV-B, oxidation-reduction process, response to karrikin | alcohol dehydrogenase (NADP+) activity, nucleotide binding, oxidoreductase activity | cytoplasm, cytosol, plasma membrane |
| ss715632215 | 4596 | Glyma18g50790 | 59805076 | 59809455 | brassinosteroid biosynthetic process, oxidation-reduction process | monooxygenase activity, iron ion binding, electron carrier activity | extracellular region |
| ss715633191 | Within gene | Glyma19g01880 | 1499557 | 1505085 | oligopeptide transport, response to nematode | amino acid transmembrane transporter activity | plasma membrane |
| ss715636101 | 1029 | Glyma19g04976 | 5206814 | 5225736 | protein N-linked glycosylation | - | mitochondrion, plasma membrane |
| ss715633673 | 24395 | Glyma19g24451 | 30045006 | 30045828 | regulation of transcription, DNA-dependent, response to ethylene stimulus, response to auxin stimulus, response to sucrose stimulus, flavonoid biosynthetic process, response to UV-B | DNA binding, sequence-specific DNA binding transcription factor activity | nucleus |
| ss715634898 | Within gene | Glyma19g31910 | 39678756 | 39691436 | chromatin silencing, microtubule-based movement, histone H3-K9 methylation | microtubule motor activity, ATP binding | cytoplasm |
| **Average irrigated (Ave_IR)** | | | | | | | |
| ss715579037 | Within gene | Glyma01g03850 | 3312868 | 3318968 | potassium ion transport, potassium ion transmembrane transport | potassium ion transmembrane transporter activity | plasma membrane, membrane |
| ss715583262 | Within gene | Glyma02g44530 | 49077298 | 49085242 | trichome morphogenesis, root hair cell differentiation, hydrogen peroxide biosynthetic process, galactolipid biosynthetic process | - | mitochondrion, chloroplast |
| ss715586436 | 9706 | Glyma03g39650 | 45654154 | 45658506 | regulation of transcription, DNA-dependent, systemic acquired resistance, salicylic acid mediated signaling pathway, jasmonic acid mediated signaling pathway, leaf morphogenesis, regulation of hydrogen peroxide metabolic process, regulation of plant-type hypersensitive response, negative regulation of defense response | sequence-specific DNA binding transcription factor activity, zinc ion binding | nucleus |
| ss715587848 | 7971 | Glyma04g32336 | 36916833 | 36918458 | - | - | - |
| ss715597242 | 9850 | Glyma07g29430 | 34289062 | 34290312 | - | copper ion binding, electron carrier activity | extracellular region |
| ss715614457 | Within gene | Glyma13g22790 | 26276973 | 26281330 | protein phosphorylation, N-terminal protein myristoylation | protein serine/threonine kinase activity, ATP binding | plasma membrane |
| ss715625320 | Within gene | Glyma16g07215 | 6480148 | 6483785 | signal transduction | kinase activity | plasma membrane |
| ss715626698 | 3946 | Glyma17g00630 | 301887 | 307625 | regulation of transcription, DNA-dependent | DNA binding, zinc ion binding, methylated histone residue binding | nucleus, cytosol |
| ss715633673 | 24395 | Glyma19g24451 | 30045006 | 30045828 | regulation of transcription, DNA-dependent, response to ethylene stimulus, response to auxin stimulus, response to sucrose stimulus, flavonoid biosynthetic process, response to UV-B | DNA binding, sequence-specific DNA binding transcription factor activity | nucleus |
| **Average drought (Ave_DR)** | | | | | | | |
| ss715585976 | 2918 | Glyma03g33220 | 40870013 | 40871970 | root morphogenesis | - | chloroplast |
| ss715597294 | 7445 | Glyma07g30040 | 35111248 | 35123164 | response to temperature stimulus, systemic acquired resistance, salicylic acid mediated signaling pathway, jasmonic acid mediated signaling pathway, negative regulation of flower development, leaf morphogenesis, regulation of plant-type hypersensitive response, negative regulation of defense response, callose deposition in cell wall | sequence-specific DNA binding transcription factor activity | nucleus |
| ss715601736 | 17995 | Glyma08g40330 | 40030654 | 40032399 | response to cytokinin stimulus, response to red light, red light signaling pathway, regulation of circadian rhythm | phosphorelay response regulator activity | nucleus, cytoplasm |
| ss715603468 | 24710 | Glyma09g03900 | 2824460 | 2827541 | regulation of transcription, DNA-dependent, response to nematode, response to auxin stimulus | sequence-specific DNA binding transcription factor activity | nucleus |
| ss715631039 | 9898 | Glyma18g40761 | 49442788 | 49445447 | response to cold, response to water deprivation, response to salt stress, oxidation-reduction process | alcohol dehydrogenase (NADP+) activity, steroid dehydrogenase activity, oxidoreductase activity | cytoplasm, chloroplast |
| **Average across environments (AAE)** | | | | | | | |
| ss715579037 | Within gene | Glyma01g03850 | 3312868 | 3318968 | potassium ion transport, potassium ion transmembrane transport | potassium ion transmembrane transporter activity | plasma membrane, membrane |
| ss715585976 | 2918 | Glyma03g33220 | 40870013 | 40871970 | root morphogenesis | - | chloroplast |
| ss715597294 | 7445 | Glyma07g30040 | 35111248 | 35123164 | response to temperature stimulus, systemic acquired resistance, salicylic acid mediated signaling pathway, jasmonic acid mediated signaling pathway, negative regulation of flower development, leaf morphogenesis, regulation of plant-type hypersensitive response, negative regulation of defense response, callose deposition in cell wall | sequence-specific DNA binding transcription factor activity | nucleus |
| ss715604906 | 17991 | Glyma09g05810 | 4560192 | 4564352 | response to hypoxia, mRNA processing | nucleic acid binding, ATP-dependent helicase activity | nucleus |
| ss715603638 | 3414 | Glyma09g26510 | 32991144 | 32992955 | respiratory burst involved in defense response, response to chitin, intracellular signal transduction | sequence-specific DNA binding transcription factor activity | nucleus |
| ss715613260 | Within gene | Glyma12g08520 | 6266324 | 6269199 | cell wall macromolecule catabolic process, cell wall biogenesis | hydrolase activity, hydrolyzing O-glycosyl compounds, xyloglucan:xyloglucosyl transferase activity | cell wall |
| ss715633673 | 24395 | Glyma19g24451 | 30045006 | 30045828 | regulation of transcription, DNA-dependent, response to ethylene stimulus, response to auxin stimulus, response to sucrose stimulus, flavonoid biosynthetic process, response to UV-B | DNA binding, sequence-specific DNA binding transcription factor activity | nucleus |
